# Supplementary material for: A Systematic Review and Meta-Analysis on the Effect of Nature Exposure Dose on Adults with Mental Illness
Source: Behav Sci (Basel). 2025 Jan 31;15(2):153. doi: 10.3390/bs15020153 (PMC11851813; doi:10.3390/bs15020153)
Supplement: Supplementary file 1 [file behavsci-15-00153-s001.zip › Supplementary A for dosage-Search-Strategies-PRISMA-S.pdf]

Supplementary A: Search Strategies and PRISMA-S Checklist

Search Strategies

All database search results retrieved on June 19-24, 2020.

Ovid (MEDLINE)

1 Mental Health/ (37,907)

2 mental*.ti. or ((mental* or psychological* or psychic) adj2 (health or care or cares or condition* or factor* or help or hygiene* or service* or state or states or status* or "well-being*" or "well being*" or wellbeing* or wellness*)).ti,ab,kw. (271,454)

3 mental disorders/ or anxiety disorders/ or agoraphobia/ or anxiety, separation/ or neurocirculatory asthenia/ or neurotic disorders/ or obsessive-compulsive disorder/ or hoarding disorder/ or panic disorder/ or phobic disorders/ or phobia, social/ or "bipolar and related disorders"/ or bipolar disorder/ or "disruptive, impulse control, and conduct disorders"/ or firesetting behavior/ or gambling/ or trichotillomania/ or dissociative disorders/ or dissociative identity disorder/ or elimination disorders/ or encopresis/ or enuresis/ or diurnal enuresis/ or nocturnal enuresis/ or "feeding and eating disorders"/ or anorexia nervosa/ or avoidant restrictive food intake disorder/ or binge-eating disorder/ or bulimia nervosa/ or diabulimia/ or food addiction/ or night eating syndrome/ or pica/ or relative energy deficiency in sport/ or female athlete triad syndrome/ or rumination syndrome/ or mood disorders/ or depressive disorder/ or depression, postpartum/ or depressive disorder, major/ or depressive disorder, treatment-resistant/ or dysthymic disorder/ or premenstrual dysphoric disorder/ or seasonal affective disorder/ or cyclothymic disorder/ or motor disorders/ or neurocognitive disorders/ or amnesia/ or alcoholic korsakoff syndrome/ or amnesia, anterograde/ or amnesia, retrograde/ or amnesia, transient global/ or cognition disorders/ or auditory perceptual disorders/ or huntington disease/ or cognitive dysfunction/ or postoperative cognitive complications/ or consciousness disorders/ or delirium/ or emergence delirium/ or dementia/ or aids dementia complex/ or alzheimer disease/ or aphasia, primary progressive/ or primary progressive nonfluent aphasia/ or creutzfeldt-jakob syndrome/ or dementia, vascular/ or dementia, multi-infarct/ or diffuse neurofibrillary tangles with calcification/ or frontotemporal lobar degeneration/ or frontotemporal dementia/ or "pick disease of the brain"/ or kluver-bucy syndrome/ or lewy body disease/ or dyslexia, acquired/ or alexia, pure/ or neurodevelopmental disorders/ or "attention deficit and disruptive behavior disorders"/ or attention deficit disorder with hyperactivity/ or conduct disorder/ or autism spectrum disorder/ or communication disorders/ or social communication disorder/ or speech sound disorder/ or developmental disabilities/ or intellectual disability/ or learning disabilities/ or dyscalculia/ or dyslexia/ or specific learning disorder/ or motor skills disorders/ or mutism/ or reactive attachment disorder/ or stereotypic movement disorder/ or tic disorders/ or tourette syndrome/ or paraphilic disorders/ or exhibitionism/ or fetishism, psychiatric/ or masochism/ or pedophilia/ or sadism/ or transvestism/ or voyeurism/ or personality disorders/ or antisocial personality disorder/ or borderline personality disorder/ or compulsive personality disorder/ or dependent personality disorder/ or histrionic

personality disorder/ or hysteria/ or paranoid personality disorder/ or passive-aggressive personality disorder/ or schizoid personality disorder/ or schizotypal personality disorder/ or "schizophrenia spectrum and other psychotic disorders"/ or affective disorders, psychotic/ or capgras syndrome/ or delusional parasitosis/ or morgellons disease/ or paranoid disorders/ or psychotic disorders/ or psychoses, substance-induced/ or psychoses, alcoholic/ or schizophrenia/ or schizophrenia, catatonic/ or schizophrenia, disorganized/ or schizophrenia, paranoid/ or shared paranoid disorder/ or sexual dysfunctions, psychological/ or dyspareunia/ or erectile dysfunction/ or gender dysphoria/ or premature ejaculation/ or "sexual and gender disorders"/ or vaginismus/ or sleep wake disorders/ or dyssomnias/ or sleep deprivation/ or sleep disorders, circadian rhythm/ or jet lag syndrome/ or sleep disorders, intrinsic/ or "disorders of excessive somnolence"/ or idiopathic hypersomnia/ or kleine-levin syndrome/ or narcolepsy/ or cataplexy/ or restless legs syndrome/ or "sleep initiation and maintenance disorders"/ or parasomnias/ or nocturnal paroxysmal dystonia/ or rem sleep parasomnias/ or rem sleep behavior disorder/ or sleep paralysis/ or sleep arousal disorders/ or night terrors/ or somnambulism/ or sleep bruxism/ or sleep-wake transition disorders/ or somatoform disorders/ or body dysmorphic disorders/ or body integrity identity disorder/ or conversion disorder/ or globus sensation/ or factitious disorders/ or munchausen syndrome/ or munchausen syndrome by proxy/ or hypochondriasis/ or neurasthenia/ or substance-related disorders/ or alcohol-related disorders/ or alcohol amnestic disorder/ or alcohol withdrawal delirium/ or alcoholic intoxication/ or alcoholism/ or binge drinking/ or wernicke encephalopathy/ or amphetamine-related disorders/ or cocaine-related disorders/ or inhalant abuse/ or marijuana abuse/ or "marijuana use"/ or narcotic-related disorders/ or opioid-induced constipation/ or opioid-related disorders/ or heroin dependence/ or morphine dependence/ or opium dependence/ or phencyclidine abuse/ or substance abuse, intravenous/ or substance abuse, oral/ or substance withdrawal syndrome/ or "tobacco use disorder"/ or "trauma and stressor related disorders"/ or adjustment disorders/ or stress disorders, traumatic/ or combat disorders/ or psychological trauma/ or historical trauma/ or stress disorders, post-traumatic/ or stress disorders, traumatic, acute/ or stress, psychological/ or burnout, psychological/ or burnout, professional/ or occupational stress/ (1,306,573)

4 ((mental adj3 (disease* or disorder* or disturb* or illness* or symptom*)) or ((attachment* or adjustment* or behavior* or behaviour* or cogniti* or conduct* or emotional* or mood or neurodevelopment* or neurocogniti* or neuropsychiatric* or personalit* or psychiatric* or psychic* or psychologic* or "substance abuse*" or "substance misuse*" or "substance use*" or stress) adj2 (disease* or disorder* or disturb* or illness* or symptom*)) or ((mental* or emotion*) adj2 (abnormal* or change* or confusion* or defect* or derangement* or disease* or disorder* or disturb* or fatigue* or ill or illness* or impair* or instabilit* or insufficianc* or lability or symptom*)) or (anxiet* or "attentional fatigue" or "behavioral disturb*" or "behavioural disturb*" or bipolar* or "cogniti* impairment*" or dementia* or depress* or insanit* or mood or "oppositional defiance" or pathopsychol* or "posttraumatic stress" or "post-traumatic stress" or psychopatholog* or psychophysiolog* or schizophrenia or schizoaffecti*) or (stress* adj2 (chronic or cope or copes or coping or daily or decrease or decreasing or family or "family related" or home or "home related" or interpersonal or internal or job or "job related" or life or maternal or mental or management or parental or perceived or physical* or physiological* or profession* or psycholog* or psychophysiolog* or psychophysical* or occupation* or reduction or response or relief or school or "school related" or social or workplace or "workplace related" or work or "work related"))).ti,ab. [mental illness from protocol] (1,168,666)

5 (agoraphobia* or amnesia* or anorexi* or aphasia* or bulimi* or "binge drinking" or "binge eating" or diabulimi* or delirium* or dyslexia* or "historical trauma*" or "intellectual disabilit*" or "learning disabilit*" or mania or manic or neuroses or neurosis or psychoses or psychosis or "relative energy deficienc*" or workoholi* or ((Alzheimer* or "attention deficit*" or "auditory perceptual*" or communication* or compulsive or conduct or consciousness or "creutzfeldt-jakob" or cyclothymic or dissociative or disruptive or drinking or dysthymic or eating or feeding or "female athlete triad" or food or hoarding or huntington* or "kluver-bucy" or learning or "lewy body" or "motor skill*" or neurotic* or obsessive or panic or paranoi* or phobia* or phobic* or psychotic* or rumination* or "seasonal affect*") adj2 (disease* or disorder* or disturb* or illness* or symptom*)) or ((alcohol* or amphetamine* or cannabis or cocaine* or drinking* or drug* or food or gambling or heroin* or inhalant or marijuana* or methamphetamine* or morphine* or narcotic* or opioid* or opium* or opiate* or smoking* or substance* or tobacco*) adj2 (addicit* or abuse* or dependen* or misuse or misuses or syndrome* or withdrawal*))).ti,ab. [other mental illnesses] (535,046)

6 or/1-5 [mental health or illness] (2,176,968)

7 (relaxation therapy/ or exercise therapy/) and (nature* or outdoor*).ti,ab,kw. (562)

8 Nature/ (853)

9 (((nature* or outdoor*) adj2 (activit* or aesthetic* or based or beauty or connect* or dose or doses or dosage* or engagement* or environment* or exercise* or experience* or exposure* or immers* or intervention* or play or rehab* or relatedness or relax* or restor* or therap* or time or "time spent" or usage)) or ("activity space*" or biophilia or "blue care" or "blue exercise*" or "blue green" or "blue prescription" or "blue space*" or ecotherap* or "environment* aesthetic*" or "green care" or "green blue" or "green exercise*" or greenery or greenness or "green prescription" or "green space*" or greenspace* or "greenway*" or greenway* or friluftsliv or "life outdoor*" or "natural environment*" or "natural landscape*" or "natural outdoor*" or "outdoor life" or "outdoor nature" or "outdoor place*" or "outdoor recreation*" or "outdoor space*" or "public open space*" or "therapeutic landscape*")).ti,ab,kw. (31,932)

10 Horticultural Therapy/ or Recreation Therapy/ (180)

11 (((forest or forests) adj3 (bath or baths or bathe or bathing* or hike or hikes or hiking* or sit or sits or sitting* or trek or treks or trekking* or therap* or walk or walks or walking*)) or ((adventure* or blue or "green blue" or forest* or green or "green blue" or horticultur* or leisure or "leisure activit*" or mountain* or nature* or outdoor* or recreation* or wilderness*) adj2 (camp or camps or camping or class or classes or course or courses or curricul* or education* or experience* or group or groups or program* or retreat or retreats or school or schools or teaching or therap*)) or (garden or gardens* or gardening* or "kneipp therap*" or "ocean therap*" or "outward bound" or "national outdoor leadership school*" or "shinrin-yoku" or "therapeutic camp" or "therapeutic camps" or "wellness retreat*") or ((farm or farming or "farm-based") adj2 (care or caring))).ti,ab,kw. [specific interventions] (19,134)

12 (((nature* or outdoor* or countryside) adj2 (ambulation* or backpacking* or bicycling* or biking* or canoeing* or camping* or caving* or climbing* or cycling* or "dog sledding*" or exercis* or fishing* or gardening* or hike or hikes or hiking* or horticulture or "horse packing*" or hunting* or kayaking* or jog or jogging* or "leisure activit*" or "mindful walk*" or mountaineer* or packrafting* or rafting* or

recreat* or run or runs or running* or "rock climbing*" or sailing* or skiing* or snorkeling* or snowboarding* or snowshoeing* or sport or sports or surfing* or swimming* or trekking* or walk or walks or walking* or walkway*)) or ("recreational hik*" or "recreational trek*" or "recreational walk*")).ti,ab. [outdoor rec activities] (1,876)

13 or/7-12 [immersive nature] (51,603)

14 6 and 13 [mental + nature] (4,876)

15 (mice or mouse or rat or rats or rodent*).ti. [rodents] (1,354,466)

16 14 not 15 [results without rodents] (4,815)

17 ("23828947" or "25509055" or "20626473").ui. [exemplars] (3)

18 16 or 17 [results include exemplars] (4,815)

19 limit 18 to yr="1990 -Current" [date filter applied] (4,460)

20 remove duplicates from 19 [final results] (4,437)

Medline (Ovid) Legend
Field codes: / = Medical Subject Heading (MeSH); ti = article title; ab = abstract; kw = keyword heading (author keywords); ui = unique identifier
Proximity operator: adj#
Truncation: *

Embase (Elsevier, embase.com)

Num.	Query	Results
#17	#16 AND (1990:py OR 1991:py OR 1992:py OR 1993:py OR 1994:py OR 1995:py OR 1996:py OR 1997:py OR 1998:py OR 1999:py OR 2000:py OR 2001:py OR 2002:py OR 2003:py OR 2004:py OR 2005:py OR 2006:py OR 2007:py OR 2008:py OR 2009:py OR 2010:py OR 2011:py OR 2012:py OR 2013:py OR 2014:py OR 2015:py OR 2016:py OR 2017:py OR 2018:py OR 2019:py OR 2020:py)	7,899
#16	#14 NOT #15	8,322
#15	mice:ti OR mouse:ti OR rat:ti OR rats:ti OR rodent*:ti	1,620,632
#14	#6 AND #13	8,815
#13	#7 OR #8 OR #9 OR #10 OR #11 OR #12	85,219
#12	(((nature* OR outdoor* OR countryside) NEAR/2 (ambulation* OR backpacking* OR bicycling* OR biking* OR canoeing* OR camping* OR caving* OR climbing* OR cycling* OR 'dog sledding*' OR exercis* OR fishing* OR gardening* OR hike OR hikes OR hiking* OR horticulture OR 'horse packing*' OR hunting* OR kayaking* OR jog OR jogging* OR 'leisure activit*' OR 'mindful walk*' OR mountaineer* OR packrafting* OR rafting* OR recreat* OR run OR runs OR running* OR 'rock climbing*' OR sailing* OR skiing* OR snorkeling* OR snowboarding* OR snowshoeing* OR sport OR sports OR surfing* OR swimming* OR trekking* OR walk OR walks OR walking* OR walkway*)):ti,ab) OR 'recreational hik*':ti,ab OR 'recreational trek*':ti,ab OR 'recreational walk*':ti,ab	2,374

#11	(((forest OR forests) NEAR/3 (bath OR baths OR bathe OR bathing* OR hike OR hikes OR hiking* OR sit OR sits OR sitting* OR trek OR treks OR trekking* OR therap* OR walk OR walks OR walking*)):ti,ab,kw) OR (((adventure* OR blue OR 'green blue' OR forest* OR green OR 'green blue' OR horticultur* OR leisure OR 'leisure activit*' OR mountain* OR nature* OR outdoor* OR recreation* OR wilderness*) NEAR/2 (camp OR camps OR camping OR class OR classes OR course OR courses OR curricul* OR education* OR experience* OR group OR groups OR program* OR retreat OR retreats OR school OR schools OR teaching OR therap*)):ti,ab,kw) OR garden:ti,ab,kw OR gardens*:ti,ab,kw OR gardening*:ti,ab,kw OR 'kneipp therap*':ti,ab,kw OR 'ocean therap*':ti,ab,kw OR 'outward bound':ti,ab,kw OR 'national outdoor leadership school*':ti,ab,kw OR 'shinrin-yoku':ti,ab,kw OR 'therapeutic camp':ti,ab,kw OR 'therapeutic camps':ti,ab,kw OR 'wellness retreat*':ti,ab,kw OR (((farm OR farming OR 'farm-based') NEAR/2 (care OR caring)):ti,ab,kw)	47,704
#10	'horticultural therapy'/mj OR 'recreational therapy'/mj	218
#9	(((nature* OR outdoor*) NEAR/2 (activit* OR aesthetic* OR based OR beauty OR connect* OR dose OR doses OR dosage* OR engagement* OR environment* OR exercise* OR experience* OR exposure* OR immers* OR intervention* OR play OR rehab* OR relatedness OR relax* OR restor* OR therap* OR time OR 'time spent' OR usage)):ti,ab,kw) OR 'activity space*':ti,ab,kw OR biophilia:ti,ab,kw OR 'blue care':ti,ab,kw OR 'blue exercise*':ti,ab,kw OR 'blue green':ti,ab,kw OR 'blue prescription':ti,ab,kw OR 'blue space*':ti,ab,kw OR ecotherap*:ti,ab,kw OR 'environment* aesthetic*':ti,ab,kw OR 'green care':ti,ab,kw OR 'green blue':ti,ab,kw OR 'green exercise*':ti,ab,kw OR greenery:ti,ab,kw OR greenness:ti,ab,kw OR 'green prescription':ti,ab,kw OR 'green space*':ti,ab,kw OR greenspace*:ti,ab,kw OR 'greenway*':ti,ab,kw OR greenway*:ti,ab,kw OR friluftsliv:ti,ab,kw OR 'life outdoor*':ti,ab,kw OR 'natural environment*':ti,ab,kw OR 'natural landscape*':ti,ab,kw OR 'natural outdoor*':ti,ab,kw OR 'outdoor life':ti,ab,kw OR 'outdoor nature':ti,ab,kw OR 'outdoor place*':ti,ab,kw OR 'outdoor recreation*':ti,ab,kw OR 'outdoor space*':ti,ab,kw OR 'public open space*':ti,ab,kw OR 'therapeutic landscape*':ti,ab,kw	37,778
#8	'natural science'/mj	1,274
#7	('relaxation training'/mj OR 'kinesiotherapy'/mj) AND (nature*:ti,ab,kw OR outdoor*:ti,ab,kw)	171
#6	#1 OR #2 OR #3 OR #4 OR #5	2,655,379
#5	agoraphobia*:ti,ab OR amnesia*:ti,ab OR anorexi*:ti,ab OR aphasia*:ti,ab OR bulimi*:ti,ab OR 'binge drinking':ti,ab OR 'binge eating':ti,ab OR diabulimi*:ti,ab OR delirium*:ti,ab OR dyslexia*:ti,ab OR 'historical trauma*':ti,ab OR 'intellectual disabilit*':ti,ab OR 'learning disabilit*':ti,ab OR mania:ti,ab OR manic:ti,ab OR neuroses:ti,ab OR neurosis:ti,ab OR psychoses:ti,ab OR psychosis:ti,ab OR 'relative energy deficienc*':ti,ab OR workoholi*:ti,ab OR (((alzheimer* OR 'attention deficit*' OR 'auditory perceptual*' OR communication* OR compulsive OR conduct OR consciousness OR 'creutzfeldt-jakob' OR cyclothymic OR dissociative OR disruptive OR drinking OR dysthymic OR eating OR feeding OR 'female athlete triad' OR food OR hoarding OR huntington* OR 'kluver-bucy' OR learning OR 'lewy body' OR 'motor skill*' OR neurotic* OR obsessive OR panic OR paranoi* OR phobia* OR phobic* OR psychotic* OR rumination* OR 'seasonal	739,555

	affect*') NEAR/2 (disease* OR disorder* OR disturb* OR illness* OR symptom*)):ti,ab) OR (((alcohol* OR amphetamine* OR cannabis OR cocaine* OR drinking* OR drug* OR food OR gambling OR heroin* OR inhalant OR marijuana* OR methamphetamine* OR morphine* OR narcotic* OR opioid* OR opium* OR opiate* OR smoking* OR substance* OR tobacco*) NEAR/2 (addicit* OR abuse* OR dependen* OR misuse OR misuses OR syndrome* OR withdrawal*)):ti,ab)	
#4	((mental NEAR/3 (disease* OR disorder* OR disturb* OR illness* OR symptom*)):ti,ab) OR (((attachment* OR adjustment* OR behavior* OR behaviour* OR cogniti* OR conduct* OR emotional* OR mood OR neurodevelopment* OR neurocogniti* OR neuropsychiatric* OR personalit* OR psychiatric* OR psychic* OR psychologic* OR 'substance abuse*' OR 'substance misuse*' OR 'substance use*' OR stress) NEAR/2 (disease* OR disorder* OR disturb* OR illness* OR symptom*)):ti,ab) OR (((mental* OR emotion*) NEAR/2 (abnormal* OR change* OR confusion* OR defect* OR derangement* OR disease* OR disorder* OR disturb* OR fatigue* OR ill OR illness* OR impair* OR instabilit* OR insufficianc* OR lability OR symptom*)):ti,ab) OR anxiet*:ti,ab OR 'attentional fatigue':ti,ab OR 'behavioral disturb*':ti,ab OR 'behavioural disturb*':ti,ab OR bipolar*:ti,ab OR 'cogniti* impairment*':ti,ab OR dementia*:ti,ab OR depress*:ti,ab OR insanit*:ti,ab OR mood:ti,ab OR 'oppositional defiance':ti,ab OR pathopsychol*:ti,ab OR 'posttraumatic stress':ti,ab OR 'post-traumatic stress':ti,ab OR psychopatholog*:ti,ab OR psychophysiolog*:ti,ab OR schizophrenia:ti,ab OR schizoaffecti*:ti,ab OR ((stress* NEAR/2 (chronic OR cope OR copes OR coping OR daily OR decrease OR decreasing OR family OR 'family related' OR home OR 'home related' OR interpersonal OR internal OR job OR 'job related' OR life OR maternal OR mental OR management OR parental OR perceived OR physical* OR physiological* OR profession* OR psycholog* OR psychophysiolog* OR psychophysical* OR occupation* OR reduction OR response OR relief OR school OR 'school related' OR social OR workplace OR 'workplace related' OR work OR 'work related')):ti,ab)	1,582,681
#3	'mental disease'/mj OR 'anxiety disorder'/mj OR 'agoraphobia'/mj OR 'separation anxiety'/mj OR 'cardiac anxiety'/mj OR 'neurosis'/mj OR 'obsessive compulsive disorder'/mj OR 'hoarding disorder'/mj OR 'panic'/mj OR 'phobia'/mj OR 'social phobia'/mj OR 'bipolar disorder'/mj OR 'impulse control disorder'/mj OR 'arson'/mj OR 'gambling'/mj OR 'trichotillomania'/mj OR 'dissociative disorder'/mj OR 'multiple personality'/mj OR 'incontinence'/mj OR 'feces incontinence'/mj OR 'enuresis'/mj OR 'diurnal enuresis'/mj OR 'nocturnal enuresis'/mj OR 'eating disorder'/mj OR 'anorexia nervosa'/mj OR 'avoidant restrictive food intake disorder'/mj OR 'binge eating disorder'/mj OR 'bulimia'/mj OR 'food addiction'/mj OR 'pica'/mj OR 'relative energy deficiency in sport'/mj OR 'female athlete triad'/mj OR 'rumination syndrome'/mj OR 'mood disorder'/mj OR 'depression'/mj OR 'postnatal depression'/mj OR 'major depression'/mj OR 'treatment resistant depression'/mj OR 'dysthymia'/mj OR 'premenstrual dysphoric disorder'/mj OR 'seasonal affective disorder'/mj OR 'cyclothymia'/mj OR 'disorders of higher cerebral function'/mj OR 'amnesia'/mj OR 'anterograde amnesia'/mj OR 'retrograde amnesia'/mj OR 'transient global amnesia'/mj OR 'cognitive defect'/mj OR 'perception deafness'/mj OR 'huntington chorea'/mj OR 'postoperative cognitive dysfunction'/mj OR 'consciousness disorder'/mj OR 'delirium'/mj OR 'emergence agitation'/mj OR 'dementia'/mj OR 'hiv associated	1,351,794

	dementia'/mj OR 'alzheimer disease'/mj OR 'primary progressive aphasia'/mj OR 'progressive nonfluent aphasia'/mj OR 'creutzfeldt jakob disease'/mj OR 'multiinfarct dementia'/mj OR 'diffuse neurofibrillary tangles with calcification'/mj OR 'frontotemporal dementia'/mj OR 'pick presenile dementia'/mj OR 'kluver bucy syndrome'/mj OR 'diffuse lewy body disease'/mj OR 'alexia'/mj OR 'alexia without agraphia'/mj OR 'attention deficit disorder'/mj OR 'conduct disorder'/mj OR 'autism'/mj OR 'communication disorder'/mj OR 'speech sound disorder'/mj OR 'developmental disorder'/mj OR 'intellectual impairment'/mj OR 'learning disorder'/mj OR 'dyscalculia'/mj OR 'dyslexia'/mj OR 'psychomotor disorder'/mj OR 'mutism'/mj OR 'psychosocial disorder'/mj OR 'motor dysfunction'/mj OR 'tic'/mj OR 'gilles de la tourette syndrome'/mj OR 'paraphilic disorder'/mj OR 'exhibitionism disorder'/mj OR 'fetishism disorder'/mj OR 'masochism'/mj OR 'pedophilia'/mj OR 'sadism'/mj OR 'cross-dressing'/mj OR 'voyeurism'/mj OR 'personality disorder'/mj OR 'antisocial personality disorder'/mj OR 'borderline state'/mj OR 'compulsive personality disorder'/mj OR 'dependent personality disorder'/mj OR 'histrionic personality disorder'/mj OR 'hysteria'/mj OR 'paranoid personality disorder'/mj OR 'passive aggressive personality disorder'/mj OR 'schizoidism'/mj OR 'schizotypal personality disorder'/mj OR 'affective psychosis'/mj OR 'capgras syndrome'/mj OR 'delusional parasitosis'/mj OR 'morgellons disease'/mj OR 'paranoid psychosis'/mj OR 'psychosis'/mj OR 'alcohol psychosis'/mj OR 'schizophrenia'/mj OR 'catatonic schizophrenia'/mj OR 'hebephrenia'/mj OR 'paranoid schizophrenia'/mj OR 'shared psychotic disorder'/mj OR 'psychosexual disorder'/mj OR 'dyspareunia'/mj OR 'erectile dysfunction'/mj OR 'gender dysphoria'/mj OR 'premature ejaculation'/mj OR 'sexual dysfunction'/mj OR 'vaginism'/mj OR 'sleep disorder'/mj OR 'sleep deprivation'/mj OR 'circadian rhythm sleep disorder'/mj OR 'jet lag'/mj OR 'somnolence'/mj OR 'hypersomnia'/mj OR 'narcolepsy'/mj OR 'cataplexy'/mj OR 'restless legs syndrome'/mj OR 'insomnia'/mj OR 'parasomnia'/mj OR 'sleep arousal disorder'/mj OR 'nightmare'/mj OR 'sleep walking'/mj OR 'sleep bruxism'/mj OR 'somatoform disorder'/mj OR 'body dysmorphic disorder'/mj OR 'conversion disorder'/mj OR 'globus hystericus'/mj OR 'factitious disease'/mj OR 'munchausen syndrome'/mj OR 'munchausen syndrome by proxy'/mj OR 'hypochondriasis'/mj OR 'neurasthenia'/mj OR 'drug dependence'/mj OR 'alcoholism'/mj OR 'korsakoff psychosis'/mj OR 'alcoholic delirium'/mj OR 'alcohol intoxication'/mj OR 'binge drinking'/mj OR 'wernicke encephalopathy'/mj OR 'amphetamine dependence'/mj OR 'cocaine dependence'/mj OR 'inhalant abuse'/mj OR 'cannabis addiction'/mj OR 'drug induced disease'/mj OR 'opioid induced constipation'/mj OR 'heroin dependence'/mj OR 'morphine addiction'/mj OR 'opiate addiction'/mj OR 'phencyclidine abuse'/mj OR 'substance abuse'/mj OR 'withdrawal syndrome'/mj OR 'tobacco dependence'/mj OR 'posttraumatic stress disorder'/mj OR 'adjustment disorder'/mj OR 'psychotrauma'/mj OR 'historical trauma'/mj OR 'mental stress'/mj OR 'burnout'/exp OR 'professional burnout'/mj OR 'job stress'/mj	
#2	mental*:ti OR (((mental* OR psychological* OR psychic) NEAR/2 (health OR care OR cares OR condition* OR factor* OR help OR hygiene* OR service* OR state OR states OR status* OR 'well-being*' OR 'well being*' OR wellbeing* OR wellness*)):ti,ab)	350,710

#1	'mental health'/mj OR 'psychological well-being'/mj	45,468

Embase Legend
Field codes: ti = article title; ab = abstract; kw = keyword; mj = focused (descriptor) index term
Proximity operator: NEAR/#
Truncation: *

PsycInfo (Ebscohost)

Interface - EBSCOhost Research Databases
Search Screen - Advanced Search
Database - APA PsycInfo
Expanders - Apply related words; Apply equivalent subjects
Search modes - Boolean/Phrase

#	Query
S17	S14 NOT S15<br>Limiters - Publication Year: 1990-2020<br>**Final Results: 1,880**
S16	S14 NOT S15
S15	TI (mice or mouse or rat or rats or rodent*)
S14	S12 AND S13
S13	S6 OR S7 OR S8 OR S9 OR S10 OR S11
S12	S1 OR S2 OR S3 OR S4 OR S5
S11	TI ((((nature* or outdoor* or countryside) N2 (ambulation* or backpacking* or bicycling* or biking* or canoeing* or camping* or caving* or climbing* or cycling* or "dog sledding*" or exercis* or fishing* or gardening* or hike or hikes or hiking* or horticulture or "horse packing*" or hunting* or kayaking* or jog or jogging* or "leisure activit*" or "mindful walk*" or mountaineer* or packrafting* or rafting* or recreat* or run or runs or running* or "rock climbing*" or sailing* or skiing* or snorkeling* or snowboarding* or snowshoeing* or sport or sports or surfing* or swimming* or trekking* or walk or walks or walking* or walkway*)) or ("recreational hik*" or "recreational trek*" or "recreational walk*"))) OR AB ((((nature* or outdoor* or countryside) N2 (ambulation* or backpacking* or bicycling* or biking* or canoeing* or camping* or caving* or climbing* or cycling* or "dog sledding*" or exercis* or fishing* or gardening* or hike or hikes or hiking* or horticulture or "horse packing*" or hunting* or kayaking* or jog or jogging* or "leisure activit*" or "mindful walk*" or mountaineer* or packrafting* or rafting* or recreat* or run or runs or running* or "rock climbing*" or sailing* or skiing* or snorkeling* or snowboarding* or snowshoeing* or sport or sports or surfing* or swimming* or trekking* or walk or walks or walking* or walkway*)) or ("recreational hik*" or "recreational trek*" or "recreational walk*")))

S10	TI ((((forest or forests) N3 (bath or baths or bathe or bathing* or hike or hikes or hiking* or sit or sits or sitting* or trek or treks or trekking* or therap* or walk or walks or walking*)) or ((adventure* or blue or "green blue" or forest* or green or "green blue" or horticultur* or leisure or "leisure activit*" or mountain* or nature* or outdoor* or recreation* or wilderness*) N2 (camp or camps or camping or class or classes or course or courses or curricul* or education* or experience* or group or groups or program* or retreat or retreats or school or schools or teaching or therap*)) or (garden or gardens* or gardening* or "kneipp therap*" or "ocean therap*" or "outward bound" or "national outdoor leadership school*" or "shinrin-yoku" or "therapeutic camp" or "therapeutic camps" or "wellness retreat*") or ((farm or farming or "farm-based") N2 (care or caring)))) OR AB ((((forest or forests) N3 (bath or baths or bathe or bathing* or hike or hikes or hiking* or sit or sits or sitting* or trek or treks or trekking* or therap* or walk or walks or walking*)) or ((adventure* or blue or "green blue" or forest* or green or "green blue" or horticultur* or leisure or "leisure activit*" or mountain* or nature* or outdoor* or recreation* or wilderness*) N2 (camp or camps or camping or class or classes or course or courses or curricul* or education* or experience* or group or groups or program* or retreat or retreats or school or schools or teaching or therap*)) or (garden or gardens* or gardening* or "kneipp therap*" or "ocean therap*" or "outward bound" or "national outdoor leadership school*" or "shinrin-yoku" or "therapeutic camp" or "therapeutic camps" or "wellness retreat*") or ((farm or farming or "farm-based") N2 (care or caring))))
S9	DE "Horticulture Therapy" OR DE "Recreation Therapy" OR DE "Adventure Therapy" OR DE "Wilderness Experience"
S8	TI ((((nature* or outdoor*) N2 (activit* or aesthetic* or based or beauty or connect* or dose or doses or dosage* or engagement* or environment* or exercise* or experience* or exposure* or immers* or intervention* or play or rehab* or relatedness or relax* or restor* or therap* or time or "time spent" or usage)) or ("activity space*" or biophilia or "blue care" or "blue exercise*" or "blue green" or "blue prescription" or "blue space*" or ecotherap* or "environment* aesthetic*" or "green care" or "green blue" or "green exercise*" or greenery or greenness or "green prescription" or "green space*" or greenspace* or "greenway*" or greenway* or friluftsliv or "life outdoor*" or "natural environment*" or "natural landscape*" or "natural outdoor*" or "outdoor life" or "outdoor nature" or "outdoor place*" or "outdoor recreation*" or "outdoor space*" or "public open space*" or "therapeutic landscape*"))) OR AB ((((nature* or outdoor*) N2 (activit* or aesthetic* or based or beauty or connect* or dose or doses or dosage* or engagement* or environment* or exercise* or experience* or exposure* or immers* or intervention* or play or rehab* or relatedness or relax* or restor* or therap* or time or "time spent" or usage)) or ("activity space*" or biophilia or "blue care" or "blue exercise*" or "blue green" or "blue prescription" or "blue space*" or ecotherap* or "environment* aesthetic*" or "green care" or "green blue" or "green exercise*" or greenery or greenness or "green prescription" or "green space*" or greenspace* or "greenway*" or greenway* or friluftsliv or "life outdoor*" or "natural environment*" or "natural landscape*" or "natural outdoor*" or "outdoor life" or "outdoor nature" or "outdoor place*" or "outdoor recreation*" or "outdoor space*" or "public open space*" or "therapeutic landscape*")))

S7	DE "Nature (Environment)"
S6	(DE "Relaxation Therapy" OR DE "Movement Therapy") AND (TI (nature* or outdoor*) OR AB (nature* or outdoor*))
S5	TI (agoraphobia* or amnesia* or anorexi* or aphasia* or bulimi* or "binge drinking" or "binge eating" or diabulimi* or delirium* or dyslexia* or "historical trauma*" or "intellectual disabilit*" or "learning disabilit*" or mania or manic or neuroses or neurosis or psychoses or psychosis or "relative energy deficienc*" or workoholi* or ((Alzheimer* or "attention deficit*" or "auditory perceptual*" or communication* or compulsive or conduct or consciousness or "creutzfeldt-jakob" or cyclothymic or dissociative or disruptive or drinking or dysthymic or eating or feeding or "female athlete triad" or food or hoarding or huntington* or "kluver-bucy" or learning or "lewy body" or "motor skill*" or neurotic* or obsessive or panic or paranoi* or phobia* or phobic* or psychotic* or rumination* or "seasonal affect*") N2 (disease* or disorder* or disturb* or illness* or symptom*)) or ((alcohol* or amphetamine* or cannabis or cocaine* or drinking* or drug* or food or gambling or heroin* or inhalant or marijuana* or methamphetamine* or morphine* or narcotic* or opioid* or opium* or opiate* or smoking* or substance* or tobacco*) N2 (addicit* or abuse* or dependen* or misuse or misuses or syndrome* or withdrawal*)))) OR AB (agoraphobia* or amnesia* or anorexi* or aphasia* or bulimi* or "binge drinking" or "binge eating" or diabulimi* or delirium* or dyslexia* or "historical trauma*" or "intellectual disabilit*" or "learning disabilit*" or mania or manic or neuroses or neurosis or psychoses or psychosis or "relative energy deficienc*" or workoholi* or ((Alzheimer* or "attention deficit*" or "auditory perceptual*" or communication* or compulsive or conduct or consciousness or "creutzfeldt-jakob" or cyclothymic or dissociative or disruptive or drinking or dysthymic or eating or feeding or "female athlete triad" or food or hoarding or huntington* or "kluver-bucy" or learning or "lewy body" or "motor skill*" or neurotic* or obsessive or panic or paranoi* or phobia* or phobic* or psychotic* or rumination* or "seasonal affect*") N2 (disease* or disorder* or disturb* or illness* or symptom*)) or ((alcohol* or amphetamine* or cannabis or cocaine* or drinking* or drug* or food or gambling or heroin* or inhalant or marijuana* or methamphetamine* or morphine* or narcotic* or opioid* or opium* or opiate* or smoking* or substance* or tobacco*) N2 (addicit* or abuse* or dependen* or misuse or misuses or syndrome* or withdrawal*))))
S4	TI (((mental N3 (disease* or disorder* or disturb* or illness* or symptom*)) or ((attachment* or adjustment* or behavior* or behaviour* or cogniti* or conduct* or emotional* or mood or neurodevelopment* or neurocogniti* or neuropsychiatric* or personalit* or psychiatric* or psychic* or psychologic* or "substance abuse*" or "substance misuse*" or "substance use*" or stress) N2 (disease* or disorder* or disturb* or illness* or symptom*)) or ((mental* or emotion*) N2 (abnormal* or change* or confusion* or defect* or derangement* or disease* or disorder* or disturb* or fatigue* or ill or illness* or impair* or instabilit* or insufficianc* or lability or symptom*)) or (anxiet* or "attentional fatigue" or "behavioral disturb*" or "behavioural disturb*" or bipolar* or "cogniti* impairment*" or dementia* or depress* or insanit* or mood or "oppositional defiance" or pathopsychol* or "posttraumatic stress" or "post-traumatic stress" or psychopatholog* or psychophysiolog* or schizophrenia or schizoaffecti*) or (stress* N2 (chronic or cope or copes or coping or daily or decrease or decreasing or family or "family related" or home

	or "home related" or interpersonal or internal or job or "job related" or life or maternal or mental or management or parental or perceived or physical* or physiological* or profession* or psycholog* or psychophysiolog* or psychophysical* or occupation* or reduction or response or relief or school or "school related" or social or workplace or "workplace related" or work or "work related")))) OR AB (((mental N3 (disease* or disorder* or disturb* or illness* or symptom*)) or ((attachment* or adjustment* or behavior* or behaviour* or cogniti* or conduct* or emotional* or mood or neurodevelopment* or neurocogniti* or neuropsychiatric* or personalit* or psychiatric* or psychic* or psychologic* or "substance abuse*" or "substance misuse*" or "substance use*" or stress) N2 (disease* or disorder* or disturb* or illness* or symptom*)) or ((mental* or emotion*) N2 (abnormal* or change* or confusion* or defect* or derangement* or disease* or disorder* or disturb* or fatigue* or ill or illness* or impair* or instabilit* or insufficianc* or lability or symptom*)) or (anxiet* or "attentional fatigue" or "behavioral disturb*" or "behavioural disturb*" or bipolar* or "cogniti* impairment*" or dementia* or depress* or insanit* or mood or "oppositional defiance" or pathopsychol* or "posttraumatic stress" or "post-traumatic stress" or psychopatholog* or psychophysiolog* or schizophrenia or schizoaffecti*) or (stress* N2 (chronic or cope or copes or coping or daily or decrease or decreasing or family or "family related" or home or "home related" or interpersonal or internal or job or "job related" or life or maternal or mental or management or parental or perceived or physical* or physiological* or profession* or psycholog* or psychophysiolog* or psychophysical* or occupation* or reduction or response or relief or school or "school related" or social or workplace or "workplace related" or work or "work related"))))
S3	DE "Mental Disorders" OR DE "Anxiety Disorders" OR DE "Agoraphobia" OR DE "Separation Anxiety" OR DE "Asthenia" OR DE "Obsessive Compulsive Disorder" OR DE "Hoarding Disorder" OR DE "Panic Disorder" OR DE "Phobias" OR DE "Social Phobia" OR DE "Bipolar Disorder" OR DE "Impulse Control Disorders" OR DE "Disruptive Behavior Disorders" OR DE "Conduct Disorder" OR DE "Arson" OR DE "Gambling Disorder" OR DE "Trichotillomania" OR DE "Dissociative Disorders" OR DE "Dissociative Identity Disorder" OR DE "Fecal Incontinence" OR DE "Human Biological Rhythms" OR DE "Urinary Incontinence" OR DE "Feeding Disorders" OR DE "Bulimia" OR DE "Food Addiction" OR DE "Binge Eating Disorder" OR DE "Binge Eating" OR DE "Pica" OR DE "Eating Disorders" OR DE "Anorexia Nervosa" OR DE "Rumination (Cognitive Process)" OR DE "Postpartum Depression" OR DE "Treatment Resistant Depression" OR DE "Seasonal Affective Disorder" OR DE "Affective Disorders" OR DE "Dysthymic Disorder" OR DE "Premenstrual Dysphoric Disorder" OR DE "Major Depression" OR DE "Cyclothymic Disorder" OR DE "Nervous System Disorders" OR DE "Neurocognitive Disorders" OR DE "Amnesia" OR DE "Anterograde Amnesia" OR DE "Global Amnesia" OR DE "Retrograde Amnesia" OR DE "Korsakoffs Psychosis" OR DE "Huntingtons Disease" OR DE "Consciousness Disorders" OR DE "Delirium" OR DE "Dementia" OR DE "AIDS Dementia Complex" OR DE "Alzheimer's Disease" OR DE "Cognitive Impairment" OR DE "Aphasia" OR DE "Creutzfeldt Jakob Syndrome" OR DE "Kluver Bucy Syndrome" OR DE "Picks Disease" OR DE "Dementia with Lewy Bodies" OR DE "Vascular Dementia" OR DE "Dementia" OR DE "Attention Deficit Disorder with Hyperactivity" OR DE "Attention Deficit Disorder" OR DE "Autism Spectrum Disorders" OR DE "Speech Disorders" OR DE "Communication Disorders" OR DE "Developmental Disabilities" OR DE "Acalculia" OR DE "Dyslexia" OR DE "Alexia" OR DE "Learning Disabilities" OR DE "Neurodevelopmental Disorders" OR DE "Learning Disorders" OR DE "Mutism" OR DE "Tics" OR

	DE "Exhibitionism" OR DE "Fetishism" OR DE "Masochism" OR DE "Pedophilia" OR DE "Sadism" OR DE "Transvestism" OR DE "Voyeurism" OR DE "Obsessive Compulsive Personality Disorder" OR DE "Personality Disorders" OR DE "Schizoid Personality Disorder" OR DE "Dependent Personality Disorder" OR DE "Borderline Personality Disorder" OR DE "Hysteria" OR DE "Histrionic Personality Disorder" OR DE "Antisocial Personality Disorder" OR DE "Passive Aggressive Personality Disorder" OR DE "Schizotypal Personality Disorder" OR DE "Capgras Syndrome" OR DE "Tourette Syndrome" OR DE "Paranoia (Psychosis)" OR DE "Paranoid Personality Disorder" OR DE "Attachment Disorders" OR DE "Schizophrenia" OR DE "Alcoholic Psychosis" OR DE "Psychosis" OR DE "Toxic Psychoses" OR DE "Schizophrenia" OR DE "Schizophrenia (Disorganized Type)" OR DE "Catatonic Schizophrenia" OR DE "Paranoid Schizophrenia" OR DE "Sexual Function Disturbances" OR DE "Erectile Dysfunction" OR DE "Premature Ejaculation" OR DE "Vaginismus" OR DE "Hypersomnia" OR DE "Kleine Levin Syndrome" OR DE "Nightmares" OR DE "Sleepwalking" OR DE "Sleep Deprivation" OR DE "Parasomnias" OR DE "Bruxism" OR DE "Body Dysmorphic Disorder" OR DE "Gender Dysphoria" OR DE "Conversion Disorder" OR DE "Dyspareunia" OR DE "Factitious Disorders" OR DE "Munchausen Syndrome" OR DE "Munchausen Syndrome by Proxy" OR DE "Hypochondriasis" OR DE "Neurasthenia" OR DE "Binge Drinking" OR DE "Alcohol Intoxication" OR DE "Marijuana Usage" OR DE "Folie A Deux" OR DE "Heroin Addiction" OR DE "Morphine Dependence" OR DE "Alcoholism" OR DE "Inhalant Abuse" OR DE "Drug Abuse" OR DE "Drug Withdrawal" OR DE "Alcohol Withdrawal" OR DE "Wernicke's Syndrome" OR DE "Restless Leg Syndrome" OR DE "Tobacco Use Disorder" OR DE "Opioid Use Disorder" OR DE "Alcohol Use Disorder" OR DE "Cannabis Use Disorder" OR DE "Substance Related and Addictive Disorders" OR DE "Substance Use Disorder" OR DE "Adjustment Disorders" OR DE "Somatoform Disorders" OR DE "Sleep Wake Disorders" OR DE "Post-Traumatic Stress" OR DE "Posttraumatic Stress Disorder" OR DE "Stress and Trauma Related Disorders" OR DE "Acute Stress Disorder" OR DE "Occupational Stress" OR DE "Stress"
S2	TI (mental* or ((mental* or psychological* or psychic) N2 (health or care or cares or condition* or factor* or help or hygiene* or service* or state or states or status* or "well-being*" or "well being*" or wellbeing* or wellness*))) OR AB (((mental* or psychological* or psychic) N2 (health or care or cares or condition* or factor* or help or hygiene* or service* or state or states or status* or "well-being*" or "well being*" or wellbeing* or wellness*)))
S1	DE "Mental Health"

<u>PsycInfo legend</u>
Field codes: TI = title; AB= abstract; DE = Descriptor term
Proximity operator: N#
Truncation: *

CINAHL Complete (Ebscohost)

Interface - EBSCOhost Research Databases
Database - CINAHL Complete
Search Screen - Advanced Search

Expanders - Apply equivalent subjects
Search modes - Boolean/Phrase

#	Query
S17	S14 NOT S15<br>Limiters - Published Date: 19900101-20201231<br>**Final Results: 2,113**
S16	S14 NOT S15
S15	TI (mice or mouse or rat or rats or rodent*)
S14	S6 AND S13
S13	S7 OR S8 OR S9 OR S10 OR S11 OR S12
S12	TI ((((nature* or outdoor* or countryside) N2 (ambulation* or backpacking* or bicycling* or biking* or canoeing* or camping* or caving* or climbing* or cycling* or "dog sledding*" or exercis* or fishing* or gardening* or hike or hikes or hiking* or horticulture or "horse packing*" or hunting* or kayaking* or jog or jogging* or "leisure activit*" or "mindful walk*" or mountaineer* or packrafting* or rafting* or recreat* or run or runs or running* or "rock climbing*" or sailing* or skiing* or snorkeling* or snowboarding* or snowshoeing* or sport or sports or surfing* or swimming* or trekking* or walk or walks or walking* or walkway*)) or ("recreational hik*" or "recreational trek*" or "recreational walk*"))) OR AB ((((nature* or outdoor* or countryside) N2 (ambulation* or backpacking* or bicycling* or biking* or canoeing* or camping* or caving* or climbing* or cycling* or "dog sledding*" or exercis* or fishing* or gardening* or hike or hikes or hiking* or horticulture or "horse packing*" or hunting* or kayaking* or jog or jogging* or "leisure activit*" or "mindful walk*" or mountaineer* or packrafting* or rafting* or recreat* or run or runs or running* or "rock climbing*" or sailing* or skiing* or snorkeling* or snowboarding* or snowshoeing* or sport or sports or surfing* or swimming* or trekking* or walk or walks or walking* or walkway*)) or ("recreational hik*" or "recreational trek*" or "recreational walk*")))
S11	TI ((((forest or forests) N3 (bath or baths or bathe or bathing* or hike or hikes or hiking* or sit or sits or sitting* or trek or treks or trekking* or therap* or walk or walks or walking*)) or ((adventure* or blue or "green blue" or forest* or green or "green blue" or horticultur* or leisure or "leisure activit*" or mountain* or nature* or outdoor* or recreation* or wilderness*) N2 (camp or camps or camping or class or classes or course or courses or curricul* or education* or experience* or group or groups or program* or retreat or retreats or school or schools or teaching or therap*)) or (garden or gardens* or gardening* or "kneipp therap*" or "ocean therap*" or "outward bound" or "national outdoor leadership school*" or "shinrin-yoku" or "therapeutic camp" or "therapeutic camps" or "wellness retreat*") or ((farm or farming or "farm-based") N2 (care or caring)))) OR AB ((((forest or forests) N3 (bath or baths or bathe or bathing* or hike or hikes or hiking* or sit or sits or sitting* or trek or treks or trekking* or therap* or walk or walks or walking*)) or ((adventure* or blue or "green blue" or forest* or green or "green blue" or

	horticultur* or leisure or "leisure activit*" or mountain* or nature* or outdoor* or recreation* or wilderness*) N2 (camp or camps or camping or class or classes or course or courses or curricul* or education* or experience* or group or groups or program* or retreat or retreats or school or schools or teaching or therap*)) or (garden or gardens* or gardening* or "kneipp therap*" or "ocean therap*" or "outward bound" or "national outdoor leadership school*" or "shinrin-yoku" or "therapeutic camp" or "therapeutic camps" or "wellness retreat*") or ((farm or farming or "farm-based") N2 (care or caring))))
S10	(MH "Recreational Therapy") OR (MH "Wilderness Experience")
S9	TI ((((nature* or outdoor*) N2 (activit* or aesthetic* or based or beauty or connect* or dose or doses or dosage* or engagement* or environment* or exercise* or experience* or exposure* or immers* or intervention* or play or rehab* or relatedness or relax* or restor* or therap* or time or "time spent" or usage)) or ("activity space*" or biophilia or "blue care" or "blue exercise*" or "blue green" or "blue prescription" or "blue space*" or ecotherap* or "environment* aesthetic*" or "green care" or "green blue" or "green exercise*" or greenery or greenness or "green prescription" or "green space*" or greenspace* or "greenway*" or greenway* or friluftsliv or "life outdoor*" or "natural environment*" or "natural landscape*" or "natural outdoor*" or "outdoor life" or "outdoor nature" or "outdoor place*" or "outdoor recreation*" or "outdoor space*" or "public open space*" or "therapeutic landscape*"))) OR AB ((((nature* or outdoor*) N2 (activit* or aesthetic* or based or beauty or connect* or dose or doses or dosage* or engagement* or environment* or exercise* or experience* or exposure* or immers* or intervention* or play or rehab* or relatedness or relax* or restor* or therap* or time or "time spent" or usage)) or ("activity space*" or biophilia or "blue care" or "blue exercise*" or "blue green" or "blue prescription" or "blue space*" or ecotherap* or "environment* aesthetic*" or "green care" or "green blue" or "green exercise*" or greenery or greenness or "green prescription" or "green space*" or greenspace* or "greenway*" or greenway* or friluftsliv or "life outdoor*" or "natural environment*" or "natural landscape*" or "natural outdoor*" or "outdoor life" or "outdoor nature" or "outdoor place*" or "outdoor recreation*" or "outdoor space*" or "public open space*" or "therapeutic landscape*")))
S8	(MH "Natural Environment")
S7	(MH "Therapeutic Exercise") AND (TI (nature* or outdoor*) OR AB (nature* or outdoor*))
S6	S1 OR S2 OR S3 OR S4 OR S5
S5	TI (agoraphobia* or amnesia* or anorexi* or aphasia* or bulimi* or "binge drinking" or "binge eating" or diabulimi* or delirium* or dyslexia* or "historical trauma*" or "intellectual disabilit*" or "learning disabilit*" or mania or manic or neuroses or neurosis or psychoses or psychosis or "relative energy deficienc*" or workoholi* or ((Alzheimer* or "attention deficit*" or "auditory perceptual*" or communication* or compulsive or conduct or consciousness or "creutzfeldt-jakob" or cyclothymic or dissociative or disruptive or drinking or dysthymic or eating or feeding or "female athlete triad" or food or hoarding or huntington* or "kluver-bucy" or learning or "lewy

	body" or "motor skill*" or neurotic* or obsessive or panic or paranoi* or phobia* or phobic* or psychotic* or rumination* or "seasonal affect*") N2 (disease* or disorder* or disturb* or illness* or symptom*)) or ((alcohol* or amphetamine* or cannabis or cocaine* or drinking* or drug* or food or gambling or heroin* or inhalant or marijuana* or methamphetamine* or morphine* or narcotic* or opioid* or opium* or opiate* or smoking* or substance* or tobacco*) N2 (addicit* or abuse* or dependen* or misuse or misuses or syndrome* or withdrawal*)))) OR AB (agoraphobia* or amnesia* or anorexi* or aphasia* or bulimi* or "binge drinking" or "binge eating" or diabulimi* or delirium* or dyslexia* or "historical trauma*" or "intellectual disabilit*" or "learning disabilit*" or mania or manic or neuroses or neurosis or psychoses or psychosis or "relative energy deficienc*" or workoholi* or ((Alzheimer* or "attention deficit*" or "auditory perceptual*" or communication* or compulsive or conduct or consciousness or "creutzfeldt-jakob" or cyclothymic or dissociative or disruptive or drinking or dysthymic or eating or feeding or "female athlete triad" or food or hoarding or huntington* or "kluver-bucy" or learning or "lewy body" or "motor skill*" or neurotic* or obsessive or panic or paranoi* or phobia* or phobic* or psychotic* or rumination* or "seasonal affect*") N2 (disease* or disorder* or disturb* or illness* or symptom*)) or ((alcohol* or amphetamine* or cannabis or cocaine* or drinking* or drug* or food or gambling or heroin* or inhalant or marijuana* or methamphetamine* or morphine* or narcotic* or opioid* or opium* or opiate* or smoking* or substance* or tobacco*) N2 (addicit* or abuse* or dependen* or misuse or misuses or syndrome* or withdrawal*))))
S4	TI (((mental N3 (disease* or disorder* or disturb* or illness* or symptom*)) or ((attachment* or adjustment* or behavior* or behaviour* or cogniti* or conduct* or emotional* or mood or neurodevelopment* or neurocogniti* or neuropsychiatric* or personalit* or psychiatric* or psychic* or psychologic* or "substance abuse*" or "substance misuse*" or "substance use*" or stress) N2 (disease* or disorder* or disturb* or illness* or symptom*)) or ((mental* or emotion*) N2 (abnormal* or change* or confusion* or defect* or derangement* or disease* or disorder* or disturb* or fatigue* or ill or illness* or impair* or instabilit* or insufficianc* or lability or symptom*)) or (anxiet* or "attentional fatigue" or "behavioral disturb*" or "behavioural disturb*" or bipolar* or "cogniti* impairment*" or dementia* or depress* or insanit* or mood or "oppositional defiance" or pathopsychol* or "posttraumatic stress" or "post-traumatic stress" or psychopatholog* or psychophysiolog* or schizophrenia or schizoaffecti*) or (stress* N2 (chronic or cope or copes or coping or daily or decrease or decreasing or family or "family related" or home or "home related" or interpersonal or internal or job or "job related" or life or maternal or mental or management or parental or perceived or physical* or physiological* or profession* or psycholog* or psychophysiolog* or psychophysical* or occupation* or reduction or response or relief or school or "school related" or social or workplace or "workplace related" or work or "work related")))) OR AB (((mental N3 (disease* or disorder* or disturb* or illness* or symptom*)) or ((attachment* or adjustment* or behavior* or behaviour* or cogniti* or conduct* or emotional* or mood or neurodevelopment* or neurocogniti* or neuropsychiatric* or personalit* or psychiatric* or psychic* or psychologic* or "substance abuse*" or "substance misuse*" or "substance use*" or stress) N2 (disease* or disorder* or disturb* or illness* or symptom*)) or ((mental* or emotion*) N2 (abnormal* or change* or confusion* or defect* or derangement* or disease* or disorder* or disturb* or fatigue* or ill or illness* or impair* or instabilit* or insufficianc* or lability or symptom*)) or (anxiet* or "attentional fatigue" or "behavioral disturb*"

	or "behavioural disturb*" or bipolar* or "cogniti* impairment*" or dementia* or depress* or insanit* or mood or "oppositional defiance" or pathopsychol* or "posttraumatic stress" or "post-traumatic stress" or psychopatholog* or psychophysiolog* or schizophrenia or schizoaffecti*) or (stress* N2 (chronic or cope or copes or coping or daily or decrease or decreasing or family or "family related" or home or "home related" or interpersonal or internal or job or "job related" or life or maternal or mental or management or parental or perceived or physical* or physiological* or profession* or psycholog* or psychophysiolog* or psychophysical* or occupation* or reduction or response or relief or school or "school related" or social or workplace or "workplace related" or work or "work related"))))
S3	(MH "Mental Disorders") OR (MH "Adjustment Disorders") OR (MH "Depression, Reactive") OR (MH "Intellectual Disability") OR (MH "Mental Disorders, Chronic") OR (MH "Attention Deficit Hyperactivity Disorder") OR (MH "Autistic Disorder") OR (MH "Communicative Disorders") OR (MH "Learning Disorders") OR (MH "Dyscalculia") OR (MH "Dyslexia") OR (MH "Dyslexia, Deep (Acquired)") OR (MH "Dyslexia, Pure (Acquired)") OR (MH "Motor Skills Disorders") OR (MH "Mutism") OR (MH "Reactive Attachment Disorder") OR (MH "Separation Anxiety") OR (MH "Social Anxiety Disorders") OR (MH "Neurotic Disorders") OR (MH "Affective Disorders") OR (MH "Anxiety Disorders") OR (MH "Dissociative Disorders") OR (MH "Factitious Disorders") OR (MH "Somatoform Disorders") OR (MH "Depression") OR (MH "Premenstrual Dysphoric Disorder") OR (MH "Seasonal Affective Disorder") OR (MH "Dysthymic Disorder") OR (MH "Depression, Postpartum") OR (MH "Obsessive-Compulsive Disorder") OR (MH "Panic Disorder") OR (MH "Phobic Disorders") OR (MH "Agoraphobia") OR (MH "Body Dysmorphic Disorder") OR (MH "Obsessive Hoarding") OR (MH "Sexual Addiction") OR (MH "Trichotillomania") OR (MH "Stress Disorders, Post-Traumatic") OR (MH "Multiple-Personality Disorder") OR (MH "Munchausen Syndrome") OR (MH "Munchausen Syndrome By Proxy") OR (MH "Hypochondriasis") OR (MH "Dementia") OR (MH "AIDS Dementia Complex") OR (MH "Lewy Body Disease") OR (MH "Pick Disease of the Brain") OR (MH "Alzheimer's Disease") OR (MH "Creutzfeldt-Jakob Syndrome") OR (MH "Dementia, Multi-Infarct") OR (MH "Dementia, Senile") OR (MH "Dementia, Presenile") OR (MH "Delusions, Parasitosis") OR (MH "Delirium") OR (MH "Psychoses, Substance-Induced") OR (MH "Psychoses, Alcoholic") OR (MH "Alcohol Withdrawal Delirium") OR (MH "Substance Withdrawal Syndrome") OR (MH "Alcohol Withdrawal Syndrome") OR (MH "Personality Disorders") OR (MH "Antisocial Personality Disorder") OR (MH "Borderline Personality Disorder") OR (MH "Compulsive Personality Disorder") OR (MH "Dependent Personality Disorder") OR (MH "Histrionic Personality Disorder") OR (MH "Passive-Aggressive Personality Disorder") OR (MH "Schizotypal Personality Disorder") OR (MH "Gambling") OR (MH "Pyromania") OR (MH "Impulse Control Disorders") OR (MH "Psychotic Disorders") OR (MH "Affective Disorders, Psychotic") OR (MH "Capgras Syndrome") OR (MH "Paranoid Disorders") OR (MH "Postpartum Psychosis") OR (MH "Schizoaffective Disorder") OR (MH "Schizophrenia") OR (MH "Bipolar Disorder") OR (MH "Cyclothymic Disorder") OR (MH "Gender Dysphoria") OR (MH "Paraphilias") OR (MH "Premature Ejaculation") OR (MH "Transvestism") OR (MH "Voyeurism") OR (MH "Sexual and Gender Disorders") OR (MH "Psychosexual Disorders") OR (MH "Dyspareunia") OR (MH "Substance Use Disorders") OR (MH "Substance Abuse") OR (MH "Substance Dependence") OR (MH "Alcohol-Related Disorders") OR (MH "Inhalant Abuse") OR (MH "Substance Abuse, Intravenous") OR (MH "Alcohol Abuse") OR (MH "Alcoholism") OR (MH "Wernicke's Encephalopathy") OR (MH "Alcoholic

	Intoxication") OR (MH "Alcohol Amnestic Disorder") OR (MH "Korsakoff Syndrome") OR (MH "Smoking") OR (MH "Sleep Disorders, Circadian Rhythm") OR (MH "Sleep Disorders") OR (MH "Dyssomnias") OR (MH "Parasomnias") OR (MH "Sleep Disorders, Intrinsic") OR (MH "Restless Legs") OR (MH "Sleep Arousal Disorders") OR (MH "Sleep-Wake Transition Disorders") OR (MH "Sleep Deprivation") OR (MH "Night Terrors") OR (MH "Somnambulism") OR (MH "Communicative Disorders") OR (MH "Aphasia") OR (MH "Dyslexia") OR (MH "Mutism") OR (MH "Compulsive Behavior") OR (MH "Eating Disorders") OR (MH "Enuresis") OR (MH "Anorexia") OR (MH "Anorexia Nervosa") OR (MH "Binge Eating Disorder") OR (MH "Bulimia Nervosa") OR (MH "Female Athlete Triad") OR (MH "Night Eating Syndrome") OR (MH "Pica") OR (MH "Enuresis, Nocturnal") OR (MH "Hysteria") OR (MH "Tic") OR (MH "Tourette Syndrome") OR (MH "Psychomotor Disorders") OR (MH "Stress") OR (MH "Stress, Psychological") OR (MH "Stress, Occupational") OR (MH "Burnout, Professional") OR (MH "Amnesia") OR (MH "Cognition Disorders") OR (MH "Consciousness Disorders") OR (MH "Delirium") OR (MH "Dementia") OR (MH "Depersonalization") OR (MH "AIDS Dementia Complex") OR (MH "Alzheimer's Disease") OR (MH "Creutzfeldt-Jakob Syndrome") OR (MH "Dementia, Vascular") OR (MH "Frontotemporal Lobar Degeneration") OR (MH "Lewy Body Disease") OR (MH "Creutzfeldt-Jakob Disease, Variant") OR (MH "Dementia, Multi-Infarct") OR (MH "Frontotemporal Dementia") OR (MH "Pick Disease of the Brain") OR (MH "Delirium, Dementia, Amnestic, Cognitive Disorders") OR (MH "Depression") OR (MH "Binge Drinking") OR (MH "Alcoholism") OR (MH "Alcoholic Intoxication") OR (MH "Korsakoff Syndrome") OR (MH "Alcohol Amnestic Disorder") OR (MH "Alcohol-Related Disorders") OR (MH "Substance Dependence")
S2	TI (mental* or ((mental* or psychological* or psychic) N2 (health or care or cares or condition* or factor* or help or hygiene* or service* or state or states or status* or "well-being*" or "well being*" or wellbeing* or wellness*))) OR AB (((mental* or psychological* or psychic) N2 (health or care or cares or condition* or factor* or help or hygiene* or service* or state or states or status* or "well-being*" or "well being*" or wellbeing* or wellness*)))
S1	(MH "Mental Health")

<u>CINAHL legend</u>
Field codes: TI = title; AB= abstract; MH = CINAHL Exact Subject Headings
Proximity operator: N#
Truncation: *

SportDiscus (Ebscohost)

Interface - EBSCOhost Research Databases
Search Screen - Advanced Search
Database - SPORTDiscus with Full Text
Expanders - Apply related words; Apply equivalent subjects
Search modes - Boolean/Phrase

S16	S13 NOT S14<br>Limiters - Published Date: 19900101-20201231<br>**Final Results: 348**
S15	S13 NOT S14
S14	TI (mice or mouse or rat or rats or rodent*)
S13	S6 AND S12
S12	S7 OR S8 OR S9 OR S10 OR S11
S11	TI ((((nature* or outdoor* or countryside) N2 (ambulation* or backpacking* or bicycling* or biking* or canoeing* or camping* or caving* or climbing* or cycling* or "dog sledding*" or exercis* or fishing* or gardening* or hike or hikes or hiking* or horticulture or "horse packing*" or hunting* or kayaking* or jog or jogging* or "leisure activit*" or "mindful walk*" or mountaineer* or packrafting* or rafting* or recreat* or run or runs or running* or "rock climbing*" or sailing* or skiing* or snorkeling* or snowboarding* or snowshoeing* or sport or sports or surfing* or swimming* or trekking* or walk or walks or walking* or walkway*)) or ("recreational hik*" or "recreational trek*" or "recreational walk*"))) OR AB ((((nature* or outdoor* or countryside) N2 (ambulation* or backpacking* or bicycling* or biking* or canoeing* or camping* or caving* or climbing* or cycling* or "dog sledding*" or exercis* or fishing* or gardening* or hike or hikes or hiking* or horticulture or "horse packing*" or hunting* or kayaking* or jog or jogging* or "leisure activit*" or "mindful walk*" or mountaineer* or packrafting* or rafting* or recreat* or run or runs or running* or "rock climbing*" or sailing* or skiing* or snorkeling* or snowboarding* or snowshoeing* or sport or sports or surfing* or swimming* or trekking* or walk or walks or walking* or walkway*)) or ("recreational hik*" or "recreational trek*" or "recreational walk*")))
S10	TI ((((forest or forests) N3 (bath or baths or bathe or bathing* or hike or hikes or hiking* or sit or sits or sitting* or trek or treks or trekking* or therap* or walk or walks or walking*)) or ((adventure* or blue or "green blue" or forest* or green or "green blue" or horticultur* or leisure or "leisure activit*" or mountain* or nature* or outdoor* or recreation* or wilderness*) N2 (camp or camps or camping or class or classes or course or courses or curricul* or education* or experience* or group or groups or program* or retreat or retreats or school or schools or teaching or therap*)) or (garden or gardens* or gardening* or "kneipp therap*" or "ocean therap*" or "outward bound" or "national outdoor leadership school*" or "shinrin-yoku" or "therapeutic camp" or "therapeutic camps" or "wellness retreat*") or ((farm or farming or "farm-based") N2 (care or caring)))) OR AB ((((forest or forests) N3 (bath or baths or bathe or bathing* or hike or hikes or hiking* or sit or sits or sitting* or trek or treks or trekking* or therap* or walk or walks or walking*)) or ((adventure* or blue or "green blue" or forest* or green or "green blue" or horticultur* or leisure or "leisure activit*" or mountain* or nature* or outdoor* or recreation* or wilderness*) N2 (camp or camps or camping or class or classes or course or courses or curricul* or education* or experience* or group or groups or program* or retreat or retreats or school or schools or teaching or therap*)) or (garden or gardens* or gardening* or "kneipp therap*" or "ocean therap*" or "outward bound" or "national outdoor leadership school*" or "shinrin-yoku"

	or "therapeutic camp" or "therapeutic camps" or "wellness retreat*") or ((farm or farming or "farm-based") N2 (care or caring))))
S9	DE "RECREATIONAL therapy"
S8	TI ((((nature* or outdoor*) N2 (activit* or aesthetic* or based or beauty or connect* or dose or doses or dosage* or engagement* or environment* or exercise* or experience* or exposure* or immers* or intervention* or play or rehab* or relatedness or relax* or restor* or therap* or time or "time spent" or usage)) or ("activity space*" or biophilia or "blue care" or "blue exercise*" or "blue green" or "blue prescription" or "blue space*" or ecotherap* or "environment* aesthetic*" or "green care" or "green blue" or "green exercise*" or greenery or greenness or "green prescription" or "green space*" or greenspace* or "greenway*" or greenway* or friluftsliv or "life outdoor*" or "natural environment*" or "natural landscape*" or "natural outdoor*" or "outdoor life" or "outdoor nature" or "outdoor place*" or "outdoor recreation*" or "outdoor space*" or "public open space*" or "therapeutic landscape*"))) OR AB ((((nature* or outdoor*) N2 (activit* or aesthetic* or based or beauty or connect* or dose or doses or dosage* or engagement* or environment* or exercise* or experience* or exposure* or immers* or intervention* or play or rehab* or relatedness or relax* or restor* or therap* or time or "time spent" or usage)) or ("activity space*" or biophilia or "blue care" or "blue exercise*" or "blue green" or "blue prescription" or "blue space*" or ecotherap* or "environment* aesthetic*" or "green care" or "green blue" or "green exercise*" or greenery or greenness or "green prescription" or "green space*" or greenspace* or "greenway*" or greenway* or friluftsliv or "life outdoor*" or "natural environment*" or "natural landscape*" or "natural outdoor*" or "outdoor life" or "outdoor nature" or "outdoor place*" or "outdoor recreation*" or "outdoor space*" or "public open space*" or "therapeutic landscape*")))
S7	("RELAXATION therapy" OR DE "EXERCISE therapy) AND (TI (nature* OR outdoor*) OR AB (nature* OR outdoor*))
S6	S1 OR S2 OR 23 OR S4 OR S5
S5	TI (agoraphobia* or amnesia* or anorexi* or aphasia* or bulimi* or "binge drinking" or "binge eating" or diabulimi* or delirium* or dyslexia* or "historical trauma*" or "intellectual disabilit*" or "learning disabilit*" or mania or manic or neuroses or neurosis or psychoses or psychosis or "relative energy deficienc*" or workoholi* or ((Alzheimer* or "attention deficit*" or "auditory perceptual*" or communication* or compulsive or conduct or consciousness or "creutzfeldt-jakob" or cyclothymic or dissociative or disruptive or drinking or dysthymic or eating or feeding or "female athlete triad" or food or hoarding or huntington* or "kluver-bucy" or learning or "lewy body" or "motor skill*" or neurotic* or obsessive or panic or paranoi* or phobia* or phobic* or psychotic* or rumination* or "seasonal affect*") N2 (disease* or disorder* or disturb* or illness* or symptom*)) or ((alcohol* or amphetamine* or cannabis or cocaine* or drinking* or drug* or food or gambling or heroin* or inhalant or marijuana* or methamphetamine* or morphine* or narcotic* or opioid* or opium* or opiate* or smoking* or substance* or tobacco*) N2 (addicit* or abuse* or dependen* or misuse or misuses or syndrome* or withdrawal*)))) OR AB (agoraphobia*

	or amnesia* or anorexi* or aphasia* or bulimi* or "binge drinking" or "binge eating" or diabulimi* or delirium* or dyslexia* or "historical trauma*" or "intellectual disabilit*" or "learning disabilit*" or mania or manic or neuroses or neurosis or psychoses or psychosis or "relative energy deficienc*" or workoholi* or ((Alzheimer* or "attention deficit*" or "auditory perceptual*" or communication* or compulsive or conduct or consciousness or "creutzfeldt-jakob" or cyclothymic or dissociative or disruptive or drinking or dysthymic or eating or feeding or "female athlete triad" or food or hoarding or huntington* or "kluver-bucy" or learning or "lewy body" or "motor skill*" or neurotic* or obsessive or panic or paranoi* or phobia* or phobic* or psychotic* or rumination* or "seasonal affect*") N2 (disease* or disorder* or disturb* or illness* or symptom*)) or ((alcohol* or amphetamine* or cannabis or cocaine* or drinking* or drug* or food or gambling or heroin* or inhalant or marijuana* or methamphetamine* or morphine* or narcotic* or opioid* or opium* or opiate* or smoking* or substance* or tobacco*) N2 (addicit* or abuse* or dependen* or misuse or misuses or syndrome* or withdrawal*))))
S4	TI (((mental N3 (disease* or disorder* or disturb* or illness* or symptom*)) or ((attachment* or adjustment* or behavior* or behaviour* or cogniti* or conduct* or emotional* or mood or neurodevelopment* or neurocogniti* or neuropsychiatric* or personalit* or psychiatric* or psychic* or psychologic* or "substance abuse*" or "substance misuse*" or "substance use*" or stress) N2 (disease* or disorder* or disturb* or illness* or symptom*)) or ((mental* or emotion*) N2 (abnormal* or change* or confusion* or defect* or derangement* or disease* or disorder* or disturb* or fatigue* or ill or illness* or impair* or instabilit* or insufficianc* or lability or symptom*)) or (anxiet* or "attentional fatigue" or "behavioral disturb*" or "behavioural disturb*" or bipolar* or "cogniti* impairment*" or dementia* or depress* or insanit* or mood or "oppositional defiance" or pathopsychol* or "posttraumatic stress" or "post-traumatic stress" or psychopatholog* or psychophysiolog* or schizophrenia or schizoaffecti*) or (stress* N2 (chronic or cope or copes or coping or daily or decrease or decreasing or family or "family related" or home or "home related" or interpersonal or internal or job or "job related" or life or maternal or mental or management or parental or perceived or physical* or physiological* or profession* or psycholog* or psychophysiolog* or psychophysical* or occupation* or reduction or response or relief or school or "school related" or social or workplace or "workplace related" or work or "work related")))) OR AB (((mental N3 (disease* or disorder* or disturb* or illness* or symptom*)) or ((attachment* or adjustment* or behavior* or behaviour* or cogniti* or conduct* or emotional* or mood or neurodevelopment* or neurocogniti* or neuropsychiatric* or personalit* or psychiatric* or psychic* or psychologic* or "substance abuse*" or "substance misuse*" or "substance use*" or stress) N2 (disease* or disorder* or disturb* or illness* or symptom*)) or ((mental* or emotion*) N2 (abnormal* or change* or confusion* or defect* or derangement* or disease* or disorder* or disturb* or fatigue* or ill or illness* or impair* or instabilit* or insufficianc* or lability or symptom*)) or (anxiet* or "attentional fatigue" or "behavioral disturb*" or "behavioural disturb*" or bipolar* or "cogniti* impairment*" or dementia* or depress* or insanit* or mood or "oppositional defiance" or pathopsychol* or "posttraumatic stress" or "post-traumatic stress" or psychopatholog* or psychophysiolog* or schizophrenia or schizoaffecti*) or (stress* N2 (chronic or cope or copes or coping or daily or decrease or decreasing or family or "family related" or home or "home related" or interpersonal or internal or job or "job related" or life or maternal or mental or management or parental or perceived or physical* or physiological*

	or profession* or psycholog* or psychophysiolog* or psychophysical* or occupation* or reduction or response or relief or school or "school related" or social or workplace or "workplace related" or work or "work related"))))
S3	DE "MENTAL illness" OR DE "ANXIETY" OR DE "ASTHENIA" OR DE "NEUROSES" OR DE "PHOBIAS" OR DE "COMPULSIVE gambling" OR DE "FEMALE athlete triad (Syndrome)" OR DE "COMPULSIVE behavior" OR DE "BULIMIA" OR DE "ANOREXIA nervosa" OR DE "MENTAL depression" OR DE "EATING disorders" OR DE "AFFECTIVE disorders" OR DE "DEMENTIA" OR DE "PRESENILE dementia" OR DE "ALZHEIMER'S disease" OR DE "SCHIZOPHRENIA" OR DE "PICK'S disease of the brain" OR DE "MENTAL disabilities" OR DE "DYSLEXIA" OR DE "DEVELOPMENTAL disabilities" OR DE "COGNITION disorders" OR DE "COMMUNICATIVE disorders" OR DE "ATTENTION-deficit hyperactivity disorder" OR DE "MOVEMENT disorders" OR DE "JET lag" OR DE "BODY dysmorphic disorder" OR DE "INHALANT abuse" OR DE "TOBACCO use" OR DE "SUBSTANCE abuse" OR DE "AMPHETAMINE abuse" OR DE "COCAINE abuse" OR DE "HEROIN abuse" OR DE "INTRAVENOUS drug abuse" OR DE "MARIJUANA abuse" OR DE "PHENCYCLIDINE abuse" OR DE "ALCOHOLISM" OR DE "DRUG abuse" OR DE "OPIOID abuse" OR DE "AFFECTIVE disorders" OR DE "SLEEP disorders" OR DE "BURNOUT (Psychology)" OR DE "SEXUAL psychology" OR DE "POST-traumatic stress disorder" OR DE "PSYCHOLOGICAL stress"
S2	TI (mental* or ((mental* or psychological* or psychic) N2 (health or care or cares or condition* or factor* or help or hygiene* or service* or state or states or status* or "well-being*" or "well being*" or wellbeing* or wellness*))) OR AB (((mental* or psychological* or psychic) N2 (health or care or cares or condition* or factor* or help or hygiene* or service* or state or states or status* or "well-being*" or "well being*" or wellbeing* or wellness*)))
S1	DE "MENTAL health"

<u>SportDiscus legend</u>
Field codes: TI = title; AB= abstract; DE = Descriptor term
Proximity operator: N#
Truncation: *

Sociological Abstracts (ProQuest)

((((MAINSUBJECT.EXACT.EXPLODE("Mental Health") OR (MAINSUBJECT.EXACT.EXPLODE("Personality Disorders") OR MAINSUBJECT.EXACT.EXPLODE("Language Disorders") OR MAINSUBJECT.EXACT.EXPLODE("Alzheimer's Disease") OR MAINSUBJECT.EXACT.EXPLODE("Deviant Behavior") OR MAINSUBJECT.EXACT.EXPLODE("Mental Illness") OR MAINSUBJECT.EXACT.EXPLODE("Depersonalization") OR MAINSUBJECT.EXACT.EXPLODE("Drinking Behavior") OR MAINSUBJECT.EXACT.EXPLODE("Attention Deficit Disorder") OR MAINSUBJECT.EXACT.EXPLODE("Eating Disorders") OR MAINSUBJECT.EXACT.EXPLODE("Alcoholism") OR MAINSUBJECT.EXACT.EXPLODE("Stress")) OR TITLE(((mental* OR psychological* OR psychic) NEAR/2 (health OR care OR cares OR condition OR conditions OR factor OR factors OR help OR hygiene OR hygienes OR service OR services OR state OR states OR status OR statuses OR "well-being" OR "well

being" OR wellbeing OR wellness OR disease OR diseases OR disorder OR disorders OR disturb OR disturbance OR disturbances OR illness OR illnesses OR symptom OR symptoms))) OR ABSTRACT(((mental* OR psychological* OR psychic) NEAR/2 (health OR care OR cares OR condition OR conditions OR factor OR factors OR help OR hygiene OR hygienes OR service OR services OR state OR states OR status OR statuses OR "well-being" OR "well being" OR wellbeing OR wellness OR disease OR diseases OR disorder OR disorders OR disturb OR disturbance OR disturbances OR illness OR illnesses OR symptom OR symptoms)))) OR (TITLE(((attachment OR adjustment OR behavior OR behavior OR behavioral OR behaviour OR behaviours OR behavioural OR cognitive OR cognition OR compulsive OR conduct OR consciousness OR "creutzfeldt-jakob" OR cyclothymic OR dissociative OR disruptive OR drinking OR dysthymic OR eating OR emotional OR feeding OR food OR hoarding OR huntington OR "Huntington′s" OR huntingtons OR "kluver-bucy" OR learning OR "lewy body" OR "motor skill" OR "motor skills" OR mood OR neurodevelopment OR neurodevelopmental OR neurocognition OR neurocognitive OR neuropsychiatric OR obsessive OR panic OR personality OR personalities OR psychiatric OR psychic* OR psychologic* OR stress) NEAR/2 (disease OR diseases OR disorder OR disorders OR disturb OR disturbance OR disturbances OR illness OR illnesses OR symptom OR symptoms))) OR TITLE(((mental OR mentally OR emotion OR emotions OR emotional) NEAR/2 (abnormal OR change OR changes OR confusion OR confusions OR defect OR defects OR defective OR derangement OR derangements OR disease OR diseases OR disorder OR disorders OR disturb OR disturbance OR disturbances OR fatigue OR fatigues OR ill OR illness OR illnesses OR impair OR impairs OR impairment OR instability OR instabilities OR insufficiency OR insufficient OR insufficiencies OR lability OR symptom OR symptoms))) OR TITLE(((stress OR stresses OR stressful) NEAR/2 (chronic OR cope OR copes OR coping OR daily OR decrease OR decreasing OR family OR "family related" OR home OR "home related" OR interpersonal OR internal OR job OR "job related" OR life OR maternal OR mental OR management OR parental OR perceived OR physical* OR physiological* OR profession OR professions OR professional OR psycholog* OR psychophysiolog* OR psychophysical* OR occupation* OR reduction OR response OR relief OR school OR "school related" OR social OR workplace OR "workplace related" OR work OR "work related"))) OR TITLE((Alzheimer* OR "attention deficit*" OR agoraphobia OR amnesia OR anorexia OR anorexic OR aphasia OR aphasias OR anxiety OR anxieties OR "attentional fatigue" OR "behavioral disturbance" OR "behavioral disturbances" OR "behavioural disturbance" OR "behavioural disturbances" OR bipolar OR bulimia OR bulimic OR "binge drinking" OR "binge eating" OR "cognition impairment" OR "cognition impairments" OR "cognitive impairment" OR "cognitive impairments" OR diabulimia OR diabulimic OR dementia OR depress OR depression OR depressive OR delirium OR deliriums OR dyslexia OR "female athlete triad syndrome" OR "historical trauma" OR "historical traumas" OR insanity OR insanities OR "intellectual disability" OR "intellectual disabilities" OR "learning disability" OR "learning disabilities" OR mania OR manic OR mood OR neurotic OR neuroses OR neurosis OR "obsessive compulsive" OR "obsessive-compulsive" OR "oppositional defiance" OR pathopsychol* OR "posttraumatic stress" OR "post-traumatic stress" OR psychopatholog* OR psychophysiolog* OR paranoia OR paranoid OR phobia OR phobias OR phobic OR phobics OR psychotic OR psychoses OR psychosis OR "relative energy deficiency" OR "relative energy deficiencies" OR schizophrenia OR schizoaffecti* OR "seasonal affect*" OR workoholi*)) OR TITLE(((alcohol OR alcoholic OR amphetamine OR cannabis OR cocaine OR drinking OR drug OR food OR gambling OR heroin OR inhalant OR marijuana OR methamphetamine OR morphine OR narcotic OR opioid OR opioids OR opium OR opiate OR opiates OR smoking OR substance OR substances OR tobacco) NEAR/2 (addiction OR addictions OR abuse OR abuses OR dependent OR dependency OR dependencies OR misuse OR misuses OR syndrome OR

syndromes OR withdrawal OR withdrawals))))) AND ((MAINSUBJECT.EXACT("Natural Environment") OR TITLE(((nature OR nature-based OR outdoor OR outdoors) NEAR/2 (based OR connect* OR dose OR doses OR dosage* OR exercise* OR experience* OR exposure* OR immers* OR intervention* OR rehab* OR relatedness OR relax* OR restor* OR therap* OR "time spent")) OR "green care" OR "green exercise" OR "green space" OR greenspace OR greenspaces OR "green prescription" OR "blue care" OR "blue exercise" OR "blue prescription" OR ecotherapy OR ecotherapies OR friluftsliv OR "life outdoor" OR "natural environment" OR "natural environments" OR "natural landscape" OR "natural landscapes" OR "natural outdoor" OR "natural outdoors" OR "outdoor life" OR "outdoor nature" OR "outdoor place" OR "outdoor places" OR "outdoor recreation" OR "outdoor space" OR "outdoor spaces" OR "public open space" OR "public open spaces" OR "therapeutic landscape" OR "therapeutic landscapes" OR "shinrin-yoku" OR garden OR gardens OR gardening OR "outward bound") OR ABSTRACT(((nature OR nature-based OR outdoor OR outdoors) NEAR/2 (based OR connect* OR dose OR doses OR dosage* OR exercise* OR experience* OR exposure* OR immers* OR intervention* OR rehab* OR relatedness OR relax* OR restor* OR therap* OR "time spent")) OR "green care" OR "green exercise" OR "green space" OR greenspace OR greenspaces OR "green prescription" OR "blue care" OR "blue exercise" OR "blue prescription" OR ecotherapy OR ecotherapies OR friluftsliv OR "life outdoor" OR "natural environment" OR "natural environments" OR "natural landscape" OR "natural landscapes" OR "natural outdoor" OR "natural outdoors" OR "outdoor life" OR "outdoor nature" OR "outdoor place" OR "outdoor places" OR "outdoor recreation" OR "outdoor space" OR "outdoor spaces" OR "public open space" OR "public open spaces" OR "therapeutic landscape" OR "therapeutic landscapes" OR "shinrin-yoku" OR garden OR gardens OR gardening OR "outward bound")) OR (TITLE(((forest OR forests) NEAR/3 (bath OR baths OR bathe OR bathing OR bathings OR hike OR hikes OR hiking OR sit OR sits OR sitting OR trek OR treks OR trekking OR therap* OR walk OR walks OR walking)) OR ((adventure OR forest OR forests OR horticultur* OR leisure OR "leisure activity" OR "leisure activities" OR mountain OR nature OR nature-based OR outdoor OR outdoors OR recreation OR recreational OR wilderness OR wildernesses) NEAR/2 (camp OR camps OR camping OR class OR classes OR course OR courses OR curricula OR curriculum OR education OR educational OR experience OR experiences OR group OR groups OR program* OR retreat OR retreats OR school OR schools OR teaching OR therap*)) OR ((farm OR farming OR "farm-based") NEAR/2 (care OR caring))) OR ABSTRACT(((forest OR forests) NEAR/3 (bath OR baths OR bathe OR bathing OR bathings OR hike OR hikes OR hiking OR sit OR sits OR sitting OR trek OR treks OR trekking OR therap* OR walk OR walks OR walking)) OR ((adventure OR forest OR forests OR horticultur* OR leisure OR "leisure activity" OR "leisure activities" OR mountain OR nature OR nature-based OR outdoor OR outdoors OR recreation OR recreational OR wilderness OR wildernesses) NEAR/2 (camp OR camps OR camping OR class OR classes OR course OR courses OR curricula OR curriculum OR education OR educational OR experience OR experiences OR group OR groups OR program* OR retreat OR retreats OR school OR schools OR teaching OR therap*)) OR ((farm OR farming OR "farm-based") NEAR/2 (care OR caring))))) NOT TITLE(mice OR mouse OR rat OR rats OR rodent*)) AND pd(19900101-20201231) **721 results**

<u>Sociological Abstracts Legend</u>

Field codes: TITLE = Document Title; ABSTRACT = Abstract; MAINSUBJECT.EXACT.EXPLODE = Subject Heading (exact & narrower terms); MAINSUBJECT.EXACT.EXPLODE = Subject Heading (exact term); pd = Publication Date

Proximity operator: NEAR/#

Truncation: *

Cochrane Library (wiley.com)

ID	Search	Hits
#1	MeSH descriptor: [Mental Health] this term only	1416
#2	(mental* or ((mental* or psychological* or psychic) NEAR/2 (health or care or cares or condition* or factor* or help or hygiene* or service* or state or states or status* or "well-being*" or "well being*" or wellbeing* or wellness*))):ti OR (((mental* or psychological* or psychic) NEAR/2 (health or care or cares or condition* or factor* or help or hygiene* or service* or state or states or status* or "well-being*" or "well being*" or wellbeing* or wellness*))):ab	31084
#3	MeSH descriptor: [Mental Disorders] explode all trees	71219
#4	MeSH descriptor: [Stress, Psychological] explode all trees	5907
#5	(((mental NEAR/3 (disease* or disorder* or disturb* or illness* or symptom*)) or ((attachment* or adjustment* or behavior* or behaviour* or cogniti* or conduct* or emotional* or mood or neurodevelopment* or neurocogniti* or neuropsychiatric* or personalit* or psychiatric* or psychic* or psychologic* or "substance abuse*" or "substance misuse*" or "substance use*" or stress) NEAR/2 (disease* or disorder* or disturb* or illness* or symptom*)) or ((mental* or emotion*) NEAR/2 (abnormal* or change* or confusion* or defect* or derangement* or disease* or disorder* or disturb* or fatigue* or ill or illness* or impair* or instabilit* or insufficianc* or lability or symptom*)) or (anxiet* or "attentional fatigue" or "behavioral disturb*" or "behavioural disturb*" or bipolar* or "cogniti* impairment*" or dementia* or depress* or insanit* or mood or "oppositional defiance" or pathopsychol* or "posttraumatic stress" or "post-traumatic stress" or psychopatholog* or psychophysiolog* or schizophrenia or schizoaffecti*) or (stress* NEAR/2 (chronic or cope or copes or coping or daily or decrease or decreasing or family or "family related" or home or "home related" or interpersonal or internal or job or "job related" or life or maternal or mental or management or parental or perceived or physical* or physiological* or profession* or psycholog* or psychophysiolog* or psychophysical* or occupation* or reduction or response or relief or school or "school related" or social or workplace or "workplace related" or work or "work related")))):ti OR (((mental NEAR/3 (disease* or disorder* or disturb* or illness* or symptom*)) or ((attachment* or adjustment* or behavior* or behaviour* or cogniti* or conduct* or emotional* or mood or neurodevelopment* or neurocogniti* or neuropsychiatric* or personalit* or psychiatric* or psychic* or psychologic* or "substance abuse*" or "substance misuse*" or "substance use*" or stress) NEAR/2 (disease* or disorder* or disturb* or illness* or symptom*)) or ((mental* or emotion*) NEAR/2 (abnormal* or change* or confusion* or defect* or derangement* or disease* or disorder* or disturb* or fatigue* or ill or illness* or impair* or instabilit* or insufficianc* or lability or symptom*)) or (anxiet* or "attentional fatigue" or "behavioral disturb*" or "behavioural disturb*" or bipolar* or "cogniti* impairment*" or dementia* or depress* or insanit* or mood or "oppositional defiance" or pathopsychol* or "posttraumatic stress" or "post-traumatic stress" or psychopatholog* or psychophysiolog* or schizophrenia or schizoaffecti*) or (stress* NEAR/2 (chronic or cope or copes or coping or daily or decrease or decreasing or family or "family related" or home or "home related" or	155265

	interpersonal or internal or job or "job related" or life or maternal or mental or management or parental or perceived or physical* or physiological* or profession* or psycholog* or psychophysiolog* or psychophysical* or occupation* or reduction or response or relief or school or "school related" or social or workplace or "workplace related" or work or "work related")))):ab	
#6	((agoraphobia* or amnesia* or anorexi* or aphasia* or bulimi* or "binge drinking" or "binge eating" or diabulimi* or delirium* or dyslexia* or "historical trauma*" or "intellectual disabilit*" or "learning disabilit*" or mania or manic or neuroses or neurosis or psychoses or psychosis or "relative energy deficienc*" or workoholi* or ((Alzheimer* or "attention deficit*" or "auditory perceptual*" or communication* or compulsive or conduct or consciousness or "creutzfeldt-jakob" or cyclothymic or dissociative or disruptive or drinking or dysthymic or eating or feeding or "female athlete triad" or food or hoarding or huntington* or "kluver-bucy" or learning or "lewy body" or "motor skill*" or neurotic* or obsessive or panic or paranoi* or phobia* or phobic* or psychotic* or rumination* or "seasonal affect*") NEAR/2 (disease* or disorder* or disturb* or illness* or symptom*)) or ((alcohol* or amphetamine* or cannabis or cocaine* or drinking* or drug* or food or gambling or heroin* or inhalant or marijuana* or methamphetamine* or morphine* or narcotic* or opioid* or opium* or opiate* or smoking* or substance* or tobacco*) NEAR/2 (addicit* or abuse* or dependen* or misuse or misuses or syndrome* or withdrawal*)))):ti OR ((agoraphobia* or amnesia* or anorexi* or aphasia* or bulimi* or "binge drinking" or "binge eating" or diabulimi* or delirium* or dyslexia* or "historical trauma*" or "intellectual disabilit*" or "learning disabilit*" or mania or manic or neuroses or neurosis or psychoses or psychosis or "relative energy deficienc*" or workoholi* or ((Alzheimer* or "attention deficit*" or "auditory perceptual*" or communication* or compulsive or conduct or consciousness or "creutzfeldt-jakob" or cyclothymic or dissociative or disruptive or drinking or dysthymic or eating or feeding or "female athlete triad" or food or hoarding or huntington* or "kluver-bucy" or learning or "lewy body" or "motor skill*" or neurotic* or obsessive or panic or paranoi* or phobia* or phobic* or psychotic* or rumination* or "seasonal affect*") NEAR/2 (disease* or disorder* or disturb* or illness* or symptom*)) or ((alcohol* or amphetamine* or cannabis or cocaine* or drinking* or drug* or food or gambling or heroin* or inhalant or marijuana* or methamphetamine* or morphine* or narcotic* or opioid* or opium* or opiate* or smoking* or substance* or tobacco*) NEAR/2 (addicit* or abuse* or dependen* or misuse or misuses or syndrome* or withdrawal*)))):ab	56008
#7	#1 OR #2 OR #3 OR #4 OR #5 OR #6	219467
#8	MeSH descriptor: [Relaxation Therapy] this term only	1330
#9	MeSH descriptor: [Exercise Therapy] this term only	9727
#10	(nature* or outdoor*):ti OR (nature* or outdoor*):ab	14334
#11	(#8 OR #9) AND #10	125
#12	MeSH descriptor: [Nature] this term only	19
#13	((((nature* or outdoor*) NEAR/2 (activit* or aesthetic* or based or beauty or connect* or dose or doses or dosage* or engagement* or environment* or exercise* or experience* or exposure* or immers* or intervention* or play or rehab* or relatedness or relax* or restor* or therap* or time or "time spent" or usage)) or	1372

	("activity space*" or biophilia or "blue care" or "blue exercise*" or "blue green" or "blue prescription" or "blue space*" or ecotherap* or "environment* aesthetic*" or "green care" or "green blue" or "green exercise*" or greenery or greenness or "green prescription" or "green space*" or greenspace* or "greenway*" or greenway* or friluftsliv or "life outdoor*" or "natural environment*" or "natural landscape*" or "natural outdoor*" or "outdoor life" or "outdoor nature" or "outdoor place*" or "outdoor recreation*" or "outdoor space*" or "public open space*" or "therapeutic landscape*"))):ti OR ((((nature* or outdoor*) NEAR/2 (activit* or aesthetic* or based or beauty or connect* or dose or doses or dosage* or engagement* or environment* or exercise* or experience* or exposure* or immers* or intervention* or play or rehab* or relatedness or relax* or restor* or therap* or time or "time spent" or usage)) or ("activity space*" or biophilia or "blue care" or "blue exercise*" or "blue green" or "blue prescription" or "blue space*" or ecotherap* or "environment* aesthetic*" or "green care" or "green blue" or "green exercise*" or greenery or greenness or "green prescription" or "green space*" or greenspace* or "greenway*" or greenway* or friluftsliv or "life outdoor*" or "natural environment*" or "natural landscape*" or "natural outdoor*" or "outdoor life" or "outdoor nature" or "outdoor place*" or "outdoor recreation*" or "outdoor space*" or "public open space*" or "therapeutic landscape*"))):ab	
#14	MeSH descriptor: [Horticultural Therapy] this term only	16
#15	MeSH descriptor: [Recreation Therapy] this term only	18
#16	((((forest or forests) NEAR/3 (bath or baths or bathe or bathing* or hike or hikes or hiking* or sit or sits or sitting* or trek or treks or trekking* or therap* or walk or walks or walking*)) or ((adventure* or blue or "green blue" or forest* or green or "green blue" or horticultur* or leisure or "leisure activit*" or mountain* or nature* or outdoor* or recreation* or wilderness*) NEAR/2 (camp or camps or camping or class or classes or course or courses or curricul* or education* or experience* or group or groups or program* or retreat or retreats or school or schools or teaching or therap*)) or (garden or gardens* or gardening* or "kneipp therap*" or "ocean therap*" or "outward bound" or "national outdoor leadership school*" or "shinrin-yoku" or "therapeutic camp" or "therapeutic camps" or "wellness retreat*") or ((farm or farming or "farm-based") NEAR/2 (care or caring)))):ti OR ((((forest or forests) NEAR/3 (bath or baths or bathe or bathing* or hike or hikes or hiking* or sit or sits or sitting* or trek or treks or trekking* or therap* or walk or walks or walking*)) or ((adventure* or blue or "green blue" or forest* or green or "green blue" or horticultur* or leisure or "leisure activit*" or mountain* or nature* or outdoor* or recreation* or wilderness*) NEAR/2 (camp or camps or camping or class or classes or course or courses or curricul* or education* or experience* or group or groups or program* or retreat or retreats or school or schools or teaching or therap*)) or (garden or gardens* or gardening* or "kneipp therap*" or "ocean therap*" or "outward bound" or "national outdoor leadership school*" or "shinrin-yoku" or "therapeutic camp" or "therapeutic camps" or "wellness retreat*") or ((farm or farming or "farm-based") NEAR/2 (care or caring)))):ab	1560
#17	((((nature* or outdoor* or countryside) NEAR/2 (ambulation* or backpacking* or bicycling* or biking* or canoeing* or camping* or caving* or climbing* or cycling* or "dog sledding*" or exercis* or fishing* or gardening* or hike or hikes or hiking* or horticulture or "horse packing*" or hunting* or kayaking* or jog or jogging* or	279

	"leisure activit*" or "mindful walk*" or mountaineer* or packrafting* or rafting* or recreat* or run or runs or running* or "rock climbing*" or sailing* or skiing* or snorkeling* or snowboarding* or snowshoeing* or sport or sports or surfing* or swimming* or trekking* or walk or walks or walking* or walkway*)) or ("recreational hik*" or "recreational trek*" or "recreational walk*"))):ti OR ((((nature* or outdoor* or countryside) NEAR/2 (ambulation* or backpacking* or bicycling* or biking* or canoeing* or camping* or caving* or climbing* or cycling* or "dog sledding*" or exercis* or fishing* or gardening* or hike or hikes or hiking* or horticulture or "horse packing*" or hunting* or kayaking* or jog or jogging* or "leisure activit*" or "mindful walk*" or mountaineer* or packrafting* or rafting* or recreat* or run or runs or running* or "rock climbing*" or sailing* or skiing* or snorkeling* or snowboarding* or snowshoeing* or sport or sports or surfing* or swimming* or trekking* or walk or walks or walking* or walkway*)) or ("recreational hik*" or "recreational trek*" or "recreational walk*"))):ab	
#18	#11 OR #12 OR #13 OR #14 OR #15 OR #16 OR #17	2963
#19	#7 AND #18	945
#20	(mice or mouse or rat or rats or rodent*):ti	735
#21	#19 NOT #20	944
#22	#21 with Cochrane Library publication date Between Jan 1990 and Dec 2020	944*

*Specially, 22 reviews and 924 trials

<u>Cochrane Library Legend</u>
Field codes: ti = Title; ab = Abstract; kw = Keyword; MeSH descriptor = Medical Subject Heading
Proximity operator: NEAR/#
Truncation: *

Dissertations & Theses Global (ProQuest)

((((TITLE(((mental* OR psychological* OR psychic) NEAR/2 (health OR care OR cares OR condition OR conditions OR factor OR factors OR help OR hygiene OR hygienes OR service OR services OR state OR states OR status OR statuses OR "well-being" OR "well being" OR wellbeing OR wellness OR disease OR diseases OR disorder OR disorders OR disturb OR disturbance OR disturbances OR illness OR illnesses OR symptom OR symptoms))) OR ABSTRACT(((mental* OR psychological* OR psychic) NEAR/2 (health OR care OR cares OR condition OR conditions OR factor OR factors OR help OR hygiene OR hygienes OR service OR services OR state OR states OR status OR statuses OR "well-being" OR "well being" OR wellbeing OR wellness OR disease OR diseases OR disorder OR disorders OR disturb OR disturbance OR disturbances OR illness OR illnesses OR symptom OR symptoms)))) OR TITLE(((attachment OR adjustment OR behavior OR behavior OR behavioral OR behaviour OR behaviours OR behavioural OR cognitive OR cognition OR compulsive OR conduct OR consciousness OR "creutzfeldt-jakob" OR cyclothymic OR dissociative OR disruptive OR drinking OR dysthymic OR eating OR emotional OR feeding OR food OR hoarding OR huntington OR "Huntington's" OR huntingtons OR "kluver-bucy" OR learning OR "lewy body" OR "motor skill" OR "motor skills" OR mood OR neurodevelopment OR neurodevelopmental OR neurocognition OR neurocognitive OR neuropsychiatric OR obsessive OR panic OR personality OR personalities OR psychiatric OR psychic* OR psychologic* OR stress) NEAR/2 (disease OR diseases OR disorder OR disorders OR disturb OR disturbance OR disturbances OR illness OR illnesses

OR symptom OR symptoms))) OR TITLE(((mental OR mentally OR emotion OR emotions OR emotional) NEAR/2 (abnormal OR change OR changes OR confusion OR confusions OR defect OR defects OR defective OR derangement OR derangements OR disease OR diseases OR disorder OR disorders OR disturb OR disturbance OR disturbances OR fatigue OR fatigues OR ill OR illness OR illnesses OR impair OR impairs OR impairment OR instability OR instabilities OR insufficiency OR insufficient OR insufficiencies OR lability OR symptom OR symptoms))) OR TITLE(((stress OR stresses OR stressful) NEAR/2 (chronic OR cope OR copes OR coping OR daily OR decrease OR decreasing OR family OR "family related" OR home OR "home related" OR interpersonal OR internal OR job OR "job related" OR life OR maternal OR mental OR management OR parental OR perceived OR physical* OR physiological* OR profession OR professions OR professional OR psycholog* OR psychophysiolog* OR psychophysical* OR occupation* OR reduction OR response OR relief OR school OR "school related" OR social OR workplace OR "workplace related" OR work OR "work related"))) OR TITLE((Alzheimer* OR "attention deficit*" OR agoraphobia OR amnesia OR anorexia OR anorexic OR aphasia OR aphasias OR anxiety OR anxieties OR "attentional fatigue" OR "behavioral disturbance" OR "behavioral disturbances" OR "behavioural disturbance" OR "behavioural disturbances" OR bipolar OR bulimia OR bulimic OR "binge drinking" OR "binge eating" OR "cognition impairment" OR "cognition impairments" OR "cognitive impairment" OR "cognitive impairments" OR diabulimia OR diabulimic OR dementia OR depress OR depression OR depressive OR delirium OR deliriums OR dyslexia OR "female athlete triad syndrome" OR "historical trauma" OR "historical traumas" OR insanity OR insanities OR "intellectual disability" OR "intellectual disabilities" OR "learning disability" OR "learning disabilities" OR mania OR manic OR mood OR neurotic OR neuroses OR neurosis OR "obsessive compulsive" OR "obsessive-compulsive" OR "oppositional defiance" OR pathopsychol* OR "posttraumatic stress" OR "post-traumatic stress" OR psychopatholog* OR psychophysiolog* OR paranoia OR paranoid OR phobia OR phobias OR phobic OR phobics OR psychotic OR psychoses OR psychosis OR "relative energy deficiency" OR "relative energy deficiencies" OR schizophrenia OR schizoaffecti* OR "seasonal affect*" OR workoholi*)) OR TITLE(((alcohol OR alcoholic OR amphetamine OR cannabis OR cocaine OR drinking OR drug OR food OR gambling OR heroin OR inhalant OR marijuana OR methamphetamine OR morphine OR narcotic OR opioid OR opioids OR opium OR opiate OR opiates OR smoking OR substance OR substances OR tobacco) NEAR/2 (addiction OR addictions OR abuse OR abuses OR dependent OR dependency OR dependencies OR misuse OR misuses OR syndrome OR syndromes OR withdrawal OR withdrawals)))) AND ((TITLE(((nature OR nature-based OR outdoor OR outdoors) NEAR/2 (based OR connect* OR dose OR doses OR dosage* OR exercise* OR experience* OR exposure* OR immers* OR intervention* OR rehab* OR relatedness OR relax* OR restor* OR therap* OR "time spent"))) OR ABSTRACT(((nature OR nature-based OR outdoor OR outdoors) NEAR/2 (based OR connect* OR dose OR doses OR dosage* OR exercise* OR experience* OR exposure* OR immers* OR intervention* OR rehab* OR relatedness OR relax* OR restor* OR therap* OR "time spent")))) OR (TITLE("green care" OR "green exercise" OR "green space" OR greenspace OR greenspaces OR "green prescription" OR "blue care" OR "blue exercise" OR "blue prescription" OR ecotherapy OR ecotherapies OR friluftsliv OR "life outdoor" OR "natural environment" OR "natural environments" OR "natural landscape" OR "natural landscapes" OR "natural outdoor" OR "natural outdoors" OR "outdoor life" OR "outdoor nature" OR "outdoor place" OR "outdoor places" OR "outdoor recreation" OR "outdoor space" OR "outdoor spaces" OR "public open space" OR "public open spaces" OR "therapeutic landscape" OR "therapeutic landscapes" OR "shinrin-yoku" OR garden OR gardens OR gardening OR "outward bound") OR ABSTRACT("green care" OR "green exercise" OR "green space" OR greenspace OR greenspaces OR "green prescription" OR "blue care" OR "blue exercise" OR

"blue prescription" OR ecotherapy OR ecotherapies OR friluftsliv OR "life outdoor" OR "natural environment" OR "natural environments" OR "natural landscape" OR "natural landscapes" OR "natural outdoor" OR "natural outdoors" OR "outdoor life" OR "outdoor nature" OR "outdoor place" OR "outdoor places" OR "outdoor recreation" OR "outdoor space" OR "outdoor spaces" OR "public open space" OR "public open spaces" OR "therapeutic landscape" OR "therapeutic landscapes" OR "shinrin-yoku" OR garden OR gardens OR gardening OR "outward bound")) OR (TITLE(((forest OR forests) NEAR/3 (bath OR baths OR bathe OR bathing OR bathings OR hike OR hikes OR hiking OR sit OR sits OR sitting OR trek OR treks OR trekking OR therap* OR walk OR walks OR walking))) OR ABSTRACT(((forest OR forests) NEAR/3 (bath OR baths OR bathe OR bathing OR bathings OR hike OR hikes OR hiking OR sit OR sits OR sitting OR trek OR treks OR trekking OR therap* OR walk OR walks OR walking)))) OR (TITLE(((adventure OR forest OR forests OR horticultur* OR leisure OR "leisure activity" OR "leisure activities" OR mountain OR nature OR nature-based OR outdoor OR outdoors OR recreation OR recreational OR wilderness OR wildernesses) NEAR/2 (camp OR camps OR camping OR class OR classes OR course OR courses OR curricula OR curriculum OR education OR educational OR experience OR experiences OR group OR groups OR program* OR retreat OR retreats OR school OR schools OR teaching OR therap*))) OR ABSTRACT(((adventure OR forest OR forests OR horticultur* OR leisure OR "leisure activity" OR "leisure activities" OR mountain OR nature OR nature-based OR outdoor OR outdoors OR recreation OR recreational OR wilderness OR wildernesses) NEAR/2 (camp OR camps OR camping OR class OR classes OR course OR courses OR curricula OR curriculum OR education OR educational OR experience OR experiences OR group OR groups OR program* OR retreat OR retreats OR school OR schools OR teaching OR therap*)))) OR (TITLE(((farm OR farming OR "farm-based") NEAR/2 (care OR caring))) OR ABSTRACT(((farm OR farming OR "farm-based") NEAR/2 (care OR caring)))))) NOT TITLE(mice OR mouse OR rat OR rats OR rodent*)) AND pd(19900101-20201231) **1,043 results**

Dissertations & Theses Global Legend
Field codes: TITLE = Document Title; ABSTRACT = Abstract; pd = Publication Date
Proximity operator: NEAR/#
Truncation: *

Scopus (Elsevier)

(((TITLE-ABS (((mental* OR psychological* OR psychic) W/2 (health OR care OR cares OR condition OR conditions OR factor OR factors OR help OR hygiene OR hygienes OR service OR services OR state OR states OR status OR statuses OR "well-being" OR "well being" OR wellbeing OR wellness OR disease OR diseases OR disorder OR disorders OR disturb OR disturbance OR disturbances OR illness OR illnesses OR symptom OR symptoms)))) OR (TITLE (((attachment OR adjustment OR behavior OR behavior OR behavioral OR behaviour OR behaviours OR behavioural OR cognitive OR cognition OR compulsive OR conduct OR consciousness OR "creutzfeldt-jakob" OR cyclothymic OR dissociative OR disruptive OR drinking OR dysthymic OR eating OR emotional OR feeding OR food OR hoarding OR huntington OR "Huntington's" OR huntingtons OR "kluver-bucy" OR learning OR "lewy body" OR "motor skill" OR "motor skills" OR mood OR neurodevelopment OR neurodevelopmental OR neurocognition OR neurocognitive OR neuropsychiatric OR obsessive OR panic OR personality OR personalities OR psychiatric OR psychic* OR psychologic* OR stress) W/2 (disease OR diseases OR disorder OR disorders OR disturb OR disturbance OR disturbances OR illness OR illnesses OR symptom OR symptoms))) OR TITLE (((mental OR mentally OR emotion OR emotions OR emotional) W/2 (abnormal OR

change OR changes OR confusion OR confusions OR defect OR defects OR defective OR derangement OR derangements OR disease OR diseases OR disorder OR disorders OR disturb OR disturbance OR disturbances OR fatigue OR fatigues OR ill OR illness OR illnesses OR impair OR impairs OR impairment OR instability OR instabilities OR insufficiency OR insufficient OR insufficiencies OR lability OR symptom OR symptoms))) OR TITLE (((stress OR stresses OR stressful) W/2 (chronic OR cope OR copes OR coping OR daily OR decrease OR decreasing OR family OR "family related" OR home OR "home related" OR interpersonal OR internal OR job OR "job related" OR life OR maternal OR mental OR management OR parental OR perceived OR physical* OR physiological* OR profession OR professions OR professional OR psycholog* OR psychophysiolog* OR psychophysical* OR occupation* OR reduction OR response OR relief OR school OR "school related" OR social OR workplace OR "workplace related" OR work OR "work related"))) OR TITLE ((alzheimer* OR "attention deficit*" OR agoraphobia OR amnesia OR anorexia OR anorexic OR aphasia OR aphasias OR anxiety OR anxieties OR "attentional fatigue" OR "behavioral disturbance" OR "behavioral disturbances" OR "behavioural disturbance" OR "behavioural disturbances" OR bipolar OR bulimia OR bulimic OR "binge drinking" OR "binge eating" OR "cognition impairment" OR "cognition impairments" OR "cognitive impairment" OR "cognitive impairments" OR diabulimia OR diabulimic OR dementia OR depress OR depression OR depressive OR delirium OR deliriums OR dyslexia OR "female athlete triad syndrome" OR "historical trauma" OR "historical traumas" OR insanity OR insanities OR "intellectual disability" OR "intellectual disabilities" OR "learning disability" OR "learning disabilities" OR mania OR manic OR mood OR neurotic OR neuroses OR neurosis OR "obsessive compulsive" OR "obsessive-compulsive" OR "oppositional defiance" OR pathopsychol* OR "posttraumatic stress" OR "post-traumatic stress" OR psychopatholog* OR psychophysiolog* OR paranoia OR paranoid OR phobia OR phobias OR phobic OR phobics OR psychotic OR psychoses OR psychosis OR "relative energy deficiency" OR "relative energy deficiencies" OR schizophrenia OR schizoaffecti* OR "seasonal affect*" OR workoholi*)) OR TITLE (((alcohol OR alcoholic OR amphetamine OR cannabis OR cocaine OR drinking OR drug OR food OR gambling OR heroin OR inhalant OR marijuana OR methamphetamine OR morphine OR narcotic OR opioid OR opioids OR opium OR opiate OR opiates OR smoking OR substance OR substances OR tobacco) W/2 (addiction OR addictions OR abuse OR abuses OR dependent OR dependency OR dependencies OR misuse OR misuses OR syndrome OR syndromes OR withdrawal OR withdrawals))))) AND ((TITLE-ABS ((nature OR nature-based OR outdoor OR outdoors) W/2 (based OR connect* OR dose OR doses OR dosage* OR exercise* OR experience* OR exposure* OR immers* OR intervention* OR rehab* OR relatedness OR relax* OR restor* OR therap* OR "time spent"))) OR (TITLE ("green care" OR "green exercise" OR "green space" OR greenspace OR greenspaces OR "green prescription" OR "blue care" OR "blue exercise" OR "blue prescription" OR ecotherapy OR ecotherapies OR friluftsliv OR "life outdoor" OR "natural environment" OR "natural environments" OR "natural landscape" OR "natural landscapes" OR "natural outdoor" OR "natural outdoors" OR "outdoor life" OR "outdoor nature" OR "outdoor place" OR "outdoor places" OR "outdoor recreation" OR "outdoor space" OR "outdoor spaces" OR "public open space" OR "public open spaces" OR "therapeutic landscape" OR "therapeutic landscapes" OR "shinrin-yoku" OR garden OR gardens OR gardening OR "outward bound" OR ((forest OR forests) W/3 (bath OR baths OR bathe OR bathing OR bathings OR hike OR hikes OR hiking OR sit OR sits OR sitting OR trek OR treks OR trekking OR therap* OR walk OR walks OR walking)) OR ((adventure OR forest OR forests OR horticultur* OR leisure

OR "leisure activity" OR "leisure activities" OR mountain OR nature OR nature-based OR outdoor OR outdoors OR recreation OR recreational OR wilderness OR wildernesses) W/2 (camp OR camps OR camping OR class OR classes OR course OR courses OR curricula OR curriculum OR education OR educational OR experience OR experiences OR group OR groups OR program* OR retreat OR retreats OR school OR schools OR teaching OR therap*)) OR ((farm OR farming OR "farm-based") W/2 (care OR caring)))))) AND NOT (TITLE (mice OR mouse OR rat OR rats OR rodent*)) AND (LIMIT-TO (PUBYEAR , 2020) OR LIMIT-TO (PUBYEAR , 2019) OR LIMIT-TO (PUBYEAR , 2018) OR LIMIT-TO (PUBYEAR , 2017) OR LIMIT-TO (PUBYEAR , 2016) OR LIMIT-TO (PUBYEAR , 2015) OR LIMIT-TO (PUBYEAR , 2014) OR LIMIT-TO (PUBYEAR , 2013) OR LIMIT-TO (PUBYEAR , 2012) OR LIMIT-TO (PUBYEAR , 2011) OR LIMIT-TO (PUBYEAR , 2010) OR LIMIT-TO (PUBYEAR , 2009) OR LIMIT-TO (PUBYEAR , 2008) OR LIMIT-TO (PUBYEAR , 2007) OR LIMIT-TO (PUBYEAR , 2006) OR LIMIT-TO (PUBYEAR , 2005) OR LIMIT-TO (PUBYEAR , 2004) OR LIMIT-TO (PUBYEAR , 2003) OR LIMIT-TO (PUBYEAR , 2002) OR LIMIT-TO (PUBYEAR , 2001) OR LIMIT-TO (PUBYEAR , 2000) OR LIMIT-TO (PUBYEAR , 1999) OR LIMIT-TO (PUBYEAR , 1998) OR LIMIT-TO (PUBYEAR , 1997) OR LIMIT-TO (PUBYEAR , 1996) OR LIMIT-TO (PUBYEAR , 1995) OR LIMIT-TO (PUBYEAR , 1994) OR LIMIT-TO (PUBYEAR , 1993) OR LIMIT-TO (PUBYEAR , 1992) OR LIMIT-TO (PUBYEAR , 1991) OR LIMIT-TO (PUBYEAR , 1990)) **2,310 results**

Scopus Legend
Field codes: TITLE-ABS-KEY = Document Title, Abstract, Keywords
Proximity operator: W/
Truncation: *

PRISMA-S Checklist

Section/topic	#	Checklist item	Location(s) Reported
**INFORMATION SOURCES AND METHODS**			
Database name	1	Name each individual database searched, stating the platform for each.	Methods, Appendix A
Multi-database searching	2	If databases were searched simultaneously on a single platform, state the name of the platform, listing all of the databases searched.	n/a
Study registries	3	List any study registries searched.	n/a
Online resources and browsing	4	Describe any online or print source purposefully searched or browsed (e.g., tables of contents, print conference proceedings, web sites), and how this was done.	n/a
Citation searching	5	Indicate whether cited references or citing references were examined, and describe any methods used for locating cited/citing references (e.g., browsing reference lists, using a citation index, setting up email alerts for references citing included studies).	Methods
Contacts	6	Indicate whether additional studies or data were sought by contacting authors, experts, manufacturers, or others.	Methods
Other methods	7	Describe any additional information sources or search methods used.	n/a
**SEARCH STRATEGIES**			
Full search strategies	8	Include the search strategies for each database and information source, copied and pasted exactly as run.	Appendix A
Limits and restrictions	9	Specify that no limits were used, or describe any limits or restrictions applied to a search (e.g., date or time period, language, study design) and provide justification for their use.	Methods, Appendix A

Search filters	10	Indicate whether published search filters were used (as originally designed or modified), and if so, cite the filter(s) used.	n/a
Prior work	11	Indicate when search strategies from other literature reviews were adapted or reused for a substantive part or all of the search, citing the previous review(s).	n/a
Updates	12	Report the methods used to update the search(es) (e.g., rerunning searches, email alerts).	n/a
Dates of searches	13	For each search strategy, provide the date when the last search occurred.	Appendix A
**PEER REVIEW**			
Peer review	14	Describe any search peer review process.	Methods
**MANAGING RECORDS**			
Total Records	15	Document the total number of records identified from each database and other information sources.	Results, Figure 1, Appendix A
Deduplication	16	Describe the processes and any software used to deduplicate records from multiple database searches and other information sources.	Methods

PRISMA-S: An Extension to the PRISMA Statement for Reporting Literature Searches in Systematic Reviews

Rethlefsen ML, Kirtley S, Waffenschmidt S, Ayala AP, Moher D, Page MJ, Koffel JB, PRISMA-S Group.

Last updated February 27, 2020.
