# Supplementary material for: A Systematic Review and Meta-Analysis on the Effect of Nature Exposure Dose on Adults with Mental Illness
Source: Behav Sci (Basel). 2025 Jan 31;15(2):153. doi: 10.3390/bs15020153 (PMC11851813; doi:10.3390/bs15020153)
Supplement: Supplementary file 1 [file behavsci-15-00153-s001.zip › Supplementary B for dosage - List of Studies with MI.pdf]

Supplementary B. List of studies included in the meta-analysis in which the study participants had diagnosed mental illness.

Study Author (Year)	Study Setting	Study Activity	Study Measures	Study Sample Size (control group; treatment group)	Mean Age of Study Sample	Percent Male/Female	Activity Dose (in minutes)	Interval or Continuous Nature Exposure	Control Group (yes or no)	Study Sample Mental Illness Type	Study Measures Category
Antonioli (2010)	Ocean and green space	Outdoor nature program, animal care program	Hamilton Depression Rating Scale, Beck Depression Inventory, Zung Self-Rating Anxiety Scale	Treatment Group 1: Ctrl Gr: 0; Tx Gr: 12, 12, 13, 13, 12, 13	39.50, 39.50, 41.00, 41.00, 39.50, 41.00	Male %: 13.30 Female %: 86.70 Male %: 13.30 Female %: 86.70 Male %: 6.70 Female %: 93.30 Male %: 6.70 Female %: 93.30 Male %: 13.30 Female %: 86.70 Male %: 6.70 Female %: 93.30	600.00	interval	no	Depression	Depression, Anxiety
Barton (2009)	Multiple natural environments	walking	Profile of Mood States	Ctr Gr: 15.00 Tx Gr:24.00	53.00	Male %: Female %: 56.42	270.00	interval	yes	A range of mental disorders	Mood
Barton et al (2012)	Urban park	green therapy	Rosenberg Self-Esteem Scale, Profile of Mood States	Ctr Gr: 24.00 Tx Gr:15.00	51.70	Male %: Female %: 56.41	45.00	continuous	yes	A range of mental disorders	Quality of Life
Bennett et al (2017)	river	Fly fishing, fly tying, camping	Posttraumatic Stress Disorder Checklist, Military Version, Patient Health Questionnaire-9, Perceived Stress Scale, Walter Reed Functional Impairment Scale, Basic Needs Satisfaction in Life Scale, Leisure Satisfaction Scale	Ctr Gr: N/A Tx Gr: 40.00	35.0	Male %: Female %: 20	2880.00	continuous	no	PTSD	Self-Regulation, General Health, Mental Health. Stress, Depression, Quality of Life
Berman et al (2012)	Urban park	walking	Backward Digit Span, Positive and Negative Affect Scales	Ctr Gr: 19.00 Tx Gr: 19.00	26.00	Male %: Female %: 60.00	52.50	continuous	yes	depression	Affect

Bettmann et al (2017)	mountains	Wilderness therapy	Outcome Questionnaire-45, Adult Attachment Scale, Psychological Separation Index	Ctr Gr: N/A Tx Gr: 8.00, 157.00	20.20	Male %: Female %: 35.30	91440.00	continuous	no	A range of mental disorders	Mental Health, Social Functioning,
Bettmann et al (2019)	wilderness	Outdoor adventure program	Outcome Questionnaire-45, Personal Growth Initiative Scale-II, Inventory of Positive Psychological Attitudes, Balanced Index of Psychological Mindedness, Emotion Regulation Questionnaire, Attitudes Toward Seeking Professional Psychological Help Scale	Ctr Gr: N/A Tx Gr: 77.00	34.40	Male %: Female %: 22.10	8640.0	continuous	no	A range of mental disorders	Self-Regulation, Mental Health, Cognition, Quality of Life
Bielinis et al (2019)	mountains, suburban forest	Camping, peer outdoor suppose therapy, CBT, skill building, Forest Therapy	Profile of Mood States	Ctr Gr: n/a Tx Gr: 20.00 Ctr Gr: n/a Tx Gr: 27.00 Ctr Gr: n/a Tx Gr: 23.00	43.30, 44.66 39.74	Male %: 100.00 Female %: 0.00 Male %: Female %: 66.67 Male %: Female %: 39.13	5760.00 105.00	continuous	no	A range of mental disorders	Anxiety, Mood
Bird (2015)	mountains	Camping, peer outdoor support therapy, CBT, skill building	Depression Anxiety and Stres Scales-21, General Perceived Self Efficacy Scale, Life Satisfaction Questionnaire, Positive and Negative Interactions Scale	Ctr Gr: n/a Tx Gr: 20.00	43.30	Male %: 100.00 Female %: 0.00 (23)	5760.00	continuous	no	A range of mental disorders	Quality of Life, Depression, Anxiety. Stress, Social Functioning
Bragg (2014)	Multiple natural environments	Wilderness therapy	Warwick Edinburgh Mental Wellbeing Scale, Rosenberg Self-Esteem Scale,	Ctr Gr: 0 Tx Gr: 62.00	38.50	Male %: Female %: 33.30	9360.00	interval	no	A range of mental disorders	Quality of Life, Environmental Connection

			Nature Relatedness Scale, Nature Relatedness Scale, Pro-environmental behavior, Health Screen							
Corazon et al (2018)	arboretum	Nacadia nature based therapy	Binge eating episodes, Rosenberg Self-Esteem Scale, Psychological general well-being Index	Ctr Gr: 7.00 Tx Gr: 8.00	44.00	Male %: Female %: 93.30	2160.00	interval	yes	Binge eating disorder	Mental Health, Quality of Life
Cotton & Butselaar (2013)	YMCA camp	Outdoor adventure program	Rosenberg Self-Esteem Scale, Pearlin Mastery Scale, Social Connectedness Scale Revised, Social Anxiety and Distress Scale, World Health Organization Quality of Life	Ctr Gr: n/a Tx Gr: 108.00	35.50	Male %: Female %: 33.30	1440.00	interval	no	A range of mental disorders	Mental Health, Social Functioning, Self-Regulation
Crawford (2017)	Ocean	Ocean therapy program	PTSD Checklist for DSM-5, Beck Depression Inventory, General Self Efficacy Scale	Ctr Gr: n/a Tx Gr: 95.00	n/a	n/a	1500.00	interval	no	PTSD	Mental Health, Depression
Duvall & Kaplan (2014)	wilderness	Backpacking, canoeing, white water rafting, fly-fishing	Perceived Stress Scale, Attentional Function Index, Positive and Negative Affect Scale, UCLA Loneliness Scale Modified, State Hope Scale & Seeking of Noetic Goals Test	Ctr Gr: n/a Tx Gr: 95.00	n/a	Male %: Female %: 21.60	7920.00	continuous	no	A range of mental disorders	Social Functioning, Cognition, Affect
Eikenæs et al (2006)	wilderness	Integrated wilderness therapy	Symptom Check list 90, Beck Depression Inventory, Inventory of Interpersonal Problems, Phobic Avoidance Rating	Ctr Gr: 37.00 Tx Gr: 16.00	36.50	Male %: Female %: 64.20	12960.00	interval	yes	Avoidant Personality Disorder	Depression, Negative Emotions, Social Functioning

			Scale, Inventory of Interpersonal Problems								
Eko (2015)	urban lakeside	Sitting and viewing	Positive and Negative Affect Scale	Ctr Gr: 24.00 Tx Gr: 24.00	30.38	Male %: Female %: 71.00	30.00	continuous	yes	depression	Affect
Gelkopf et al (2013)	ocean	sailing	Stanford Acute Stress Reaction Questionnaire, Beck Depression Inventory, Functional Problem Scale, Perceived Control over Illness Questionnaire, Human Service Scale, Functioning Domains Questions, Hope Scale	Ctr Gr: 20.00 Tx Gr: 22.00	38.30	Male %: Female %: 0	7200.00	interval	yes	PTSD	General Health, Mental Health, Mood, Quality of Life
Gonzalez et al (2009)	farm	Horticultural therapy	Beck Depression Inventory, Attentional Function Index, Life Regard Index - Revised	Ctr Gr:n/a Tx Gr: 18.00	49.70	Male %: Female %: 83.33	4320.00	interval	no	depression	Cognition, Depression, Quality of Life
Gonzalez et al (2010)	farm	Horticultural therapy	Beck Depression Inventory, Attentional Function Index, The Brooding Scale, Perceived Restorativeness Scale	Ctr Gr:n/a Tx Gr: 28.00	44.10	Male %: Female %: 75.00	3366.00	interval	no	depression	Quality of Life, Depression, Cognition, Negative Emotions
Gonzalez et al (2011)	farm	Horticultural therapy	Beck Depression Inventory, Sense of Coherence-13	Ctr Gr:n/a Tx Gr: 28.00	44.10	Male %: Female %: 83.33	4320.00	interval	no	depression	Quality of Life
Hitter et al (2019)	greenspace	Horticultural therapy	Beck Depression Inventory, Kynurenine, Kynurenic acid	Ctr Gr: n/a Tx Gr: 8.00	21.10	Male %: Female %: 56.50	2400.00	interval	no	depression	Depression
Iwata et al (2016)	forest	Walking and socializing	Positive and Negative Affect Scale	Ctr Gr: n/a Tx Gr: 15.00	47.00	Male %: Female %: 80.00	1560.00	interval	no	A range of mental disorders	Affect
Kelley et al (1997)	wilderness	Hiking, rock climbing	Self-Efficacy Scale, Physical Self-Efficacy	Ctr Gr: 19.00	31.61	n/a	4860.00	continuous	yes	A range of	Quality of Life, Depression, Anxiety

		, rappelling, spelunking and canoeing	Scale, Generalized Self-Efficacy Scale, Rosenberg Self-Esteem Inventory, State-Trait Anxiety inventory, Brief Symptom Inventory, Beck Depression Inventory	Tx Gr: 50.00						mental disorders	
Kim et al (2009)	forest	Cognitive Behavioral Therapy	Hamilton Rating Scales for Depression, Montgomery-Asberg Depression Rating Scales, Beck Depression Inventory, Short Form-36 health survey, heart rate variability, salivary cortisol concentration	Ctr Gr: 21.00 Tx Gr: 23.00	46.08	Male %: Female %: 84.10	720.00	interval	yes	depression	Depression, General Health, Mental Health, Physiological, Quality of Life
Korpela et al (2016)	urban park	walking	Beck Depression Inventory, Short Warwick-Edinburgh Mental Well Being Scale, Restorative Outcome Scale	Ctr Gr: n/a Tx Gr: 13.00	48.00	Male %: Female %: 69.23	480.00	interval	no	1	Quality of Life, Depression
Legrand et al (2018)	mountains	running	Profile of Mood States	Ctr Gr: 18.00 Tx Gr: 18.00	26.30	Male %: Female %: 66.67	20.00	continuous	yes	1	Mood
Lundberg et al (2011)	Multiple nature environments	Adaptive sports and recreation program	World Health Organization Quality of Life-BREF, Profile of Mood States, Perceived Competence Scale	Ctr Gr: n/a Tx Gr: 18.00	n/a	Male %: Female %: 44.00	1800.00	interval	no	6	Mood, Quality of Life
Makizako et al (2019)	garden	Horticultural activity	Geriatric Depression Scale – 15, Wechsler Memory Scale – Revised, Worldist Memory Tasks	Ctr Gr: 28.00 Tx Gr: 26.00	73.05	Male %: Female %: 50.85	1500.00	interval	yes	1	Cognition, Depression

Marchand et al (2018)	reservoir	sailing	State-Trait Anxiety Inventory, Positive and Negative Affect Scale, Acceptance and Action Questionnaire 11, Five Facet Mindfulness Questionnaire	Ctr Gr: n/a Tx Gr: 22.00	45.22	Male %: Female %: 9.10	180.00	interval	yes	7	Affect, Cognition
Marchand et al (2019)	reservoir	Sailing and mindfulness	State-Trait Anxiety Inventory, Acceptance and Action Questionnaire 11, Five Facet Mindfulness Questionnaire	Ctr Gr: n/a Tx Gr: 11.00	55.00	Male %: Female %: 33.30	1440.00	interval	yes	7	Self-Regulation, Anxiety
Maund et al (2019)	wetlands	walking	Warwick-Edinburgh Mental Wellbeing Scale, Generalized Anxiety Disorder – 7, Perceived Stress Scale, Positive and Negative Affect Scale	Ctr Gr: n/a Tx Gr: 16.00	n/a	Male %: Female %: 50.00	720.00	interval	yes	6	Stress, Anxiety, Quality of Life
McCaffrey et al (2010)	Curated garden	walking	Geriatric Depression Scale	Ctr Gr: n/a Tx Gr: 40.00	71.30	n/a	1440.00	interval	No	1	Depression
McCaffrey et al (2011)	greenspace	Horticultural therapy	Geriatric Depression Scale	Ctr Gr: n/a Tx Gr: 8.00	74.10	Male %: Female %: 56.50	2400.00	continuous	No	1	Depression
Oh et al (2018)	farm	Horticultural therapy	Positive and Negative Syndrome Scale, Brief Psychiatric Rating Scale	Ctr Gr: 13.00 Tx Gr: 15.00	37.75	Male %: Female %: 30.25	1200.00	interval	Yes	9	Mental Health
Pawlowski et al (1993)	mountains	Wilderness therapy	Brief Symptom Inventory 53, Levenson Locus of Control Scales	Ctr Gr: n/a Tx Gr: 8.00 (14) Ctr Gr: n/a Tx Gr: 7.00 (14)	30.10 (26)	Male %: Female %: 11.80 (26)	7200.00 (26)	continuous	No	9 (14) 17(14)	Social Functioning, Negative Emotions, Mental Health, Anxiety, Depression, Quality of Life

Stigsdotter et al (2018)	arboretum	Nature-based therapy	Psychological General Well-Being Index	Ctr Gr: 37.00 Tx Gr: 39.00	46.40	Male %: Female %: 81.70	5400.00	interval	Yes	13	Quality of Life
Stowell et al (2018)	garden	Horticultural therapy	Depression Anxiety and Stress Scale-21, Quality of Life Enjoyment and Satisfaction Questionnaire Short Form	Ctr Gr: n/a Tx Gr: 8.00	50.80	Male %: Female %: 44.00	900.00	interval	No	6	Stress, Anxiety, Depression
Thal (2014)	Wooded state park	walking	Backward Digit Span, Stroop Color-Word Test, ADHD Symptom Scale	Ctr Gr: 20.00 Tx Gr: 20.00	20.72	Male %: Female %: 60.00	20.00	continuous	Yes	14	Cognition, Mental Health
Vella et al (2013)	wilderness	Fly-fishing	Brief Symptom Inventory-18, Positive and Negative Affect Scale	Ctr Gr: n/a Tx Gr: 74.00	47.27	Male %: Female %: 6.76	960.00	continuous	No	7	Negative Emotions, Affect
Wilson et al (2011)	Woodland natural area	Walking and a range of activituies	Short Form Health Survey-12 version 2, Warwick-Edinburgh Mental Wellbeing Scale, Scottish Physical Activity Questionnaire	Ctr Gr: n/a Tx Gr: 77.00	41.40	Male %: Female %: 25.97	1800.00	interval	No	6	Physiological, Mental Health
Wirz-Justice et al (1996)	outdoors	walking	Hamilton Depression Rating Scale, sleep duration, number of awakenings	Ctr Gr: 8.00 Tx Gr: 20.00	42.75	Male %: Female %: 92.86	420.00	interval	Yes	18	General Health, Depression, Quality of Life
