# Supplementary material for: A Systematic Review and Meta-Analysis on the Effect of Nature Exposure Dose on Adults with Mental Illness
Source: Behav Sci (Basel). 2025 Jan 31;15(2):153. doi: 10.3390/bs15020153 (PMC11851813; doi:10.3390/bs15020153)
Supplement: Supplementary file 1 [file behavsci-15-00153-s001.zip › Supplementary C for dosage_List of Studies with Sx of MI.pdf]

Supplementary C: List of Studies with Participants with Symptoms of Mental Illness Included in Meta-Analysis and Their Key Variables

Study Author (Year)	Study Setting	Study Activity	Study Measures	Study Sample Size (control group; treatment group)	Mean Age of Study Sample	Study Sample Male & Female	Nature Exposure Dose (in minutes)	Interval or Continuous Nature Dosage	Control Group	Study Measures Categories
Bahaeloo-Horeh & Assari (2008)	mountains	mountaineering	Rosenberg Self-Esteem Scale	Ctrl Gr: 0 Tx Gr: 54.00	23.30	Male %: 26.00 Female %: 74.00	1920.00	Continuous	No	Quality of Life
Bang et al (2017)	Urban forest	walking	Beck Depression Inventory, Parasympathetic nerve activity, LF/HF ratio, Systolic Blood Pressure, Diastolic Blood Pressure, Total Cholesterol, High-density lipoprotein (HDL Cholesterol), Low-density lipoprotein (LDL Cholesterol), Triglyceride (Cholesterol), Bone density (T-score), Body Mass Index, Percent of body fat, MET-mini/week (physical activity level), Health-Promoting Lifestyle Profile II	Ctrl Gr: 48.00 Tx Gr: 51.00,	24.30	Male %: 47.47 Female %: 52.53	480.00, 360.00	Interval	Yes	Depression, Physiological, General Health
Bielinis et al (2018)	Urban forest	Forest bathing	Profile of Mood States, Positive and Negative Affect Scale, Subjective Vitality Scale, Restorative Outcome Scale	Ctr Gr: 31.00 Tx Gr: 31.00	21.45	Male %: 58.06 Female %: 41.94	15.00	Continuous	yes	Quality of Life, Mood, Affect
Bodin & Hartig (2003)	Urban park	running	Exercise Induced Feeling Inventory: Revitalization, Exercise Induced Feeling Inventory: Tranquility, Negative Mood Scale: Anxiety/Depression, Negative Mood Scale: Anger, Symbol Digits Modalities Test, Digit Span Forward & Backward	Ctr Gr: 6.00 Tx Gr: 6.00	39.70 37.00	Male %: 100.00 Female %: 0.00	120.00	Interval	yes	Negative Emotions, Cognition, Quality of Life
Brooks et al (2017)	Urban park	Sitting and viewing	Positive and Negative Affect Scale, Elevating Experience Scale, Depression Anxiety and Stress Scale	Ctr Gr: 26.00 Tx Gr: 21.00	22.30	Male %: 19.15 Female %: 80.85	10.00	Continuous	yes	Stress, Anxiety, Affect,

Brown et al (2014)	Urban nature	walking	Heart Rate, Natural log of high frequency, Systolic Blood Pressure, Diastolic Blood Pressure, Body Mass Index, cardiovascular disease risk, predicted aerobic fitness, heart rate stress response, heart rate stress recovery, natural log of high frequency stress response, natural log of high frequency stress recovery, SF-8 General Health, SF-8 physical health, SF-8 Mental health	Ctr Gr: 29.00 Tx Gr: 32.00	43.25	Male %: 81.97 Female %: 18.03	320.00	Interval	yes	Depression, Quality of Life Physiological, Other Mental Health, General Health
Cervinka et al (2020)	forest	walking	Positive and Negative Affect Scale, Perceived Stress Assessment, Restorative Outcome Scale, Inclusion of Nature in the Self, Forest, Freiburg Mindfulness Inventory	Ctr Gr: n/a Tx Gr: 99.00	43.15	Male %: 36.00 Female %: 64.00	156.64	Continuous	no	Affect, Cognition, Stress
Choi & Ha (2014)	Forest	Forest experie nce interven tion	Zung Self-Rating Depression Scale, Korean Resilience Quotient Test-53: Self Regulation, Korean Resilience Quotient Test-53: Interpersonal, Korean resilience Quotient Test-53: Positivity, Korean Resilience Quotient Test-53: Total	Ctr Gr: 27.00 Tx Gr: 26.00	No data availabl e	Male %: 28.30 Female %: 71.70	480.00	Interval	Yes	Quality of Life, Depression
Chun et al (2017)	forest	Forest therapy	Beck Depression Inventory, Hamilton Depression Rating Scale, State-Trait Anxiety Inventory, Oxidative Stress Assessment, BAPs	Ctr Gr: 29.00 Tx Gr: 30.00	60.80	Male %: 67.80 Female %: 32.20	5760.00	Continuous	yes	Depression, Anxiety, Physiological
Crust et al (2013)	Countr yside, urban park	walking	Rosenberg Self-Esteem Scale, Positive and Negative Affect Scale	Ctr Gr: n/a Tx Gr: 34.00 Ctr Gr: n/a Tx Gr: 49.00	61.30, 64.10	Male %: 38.00 Female %: 62.00 Male %: 29.00 Female %: 71.00	75.00, 34.00, 49.00	Continuous	no	Quality of Life, Affect
De Brito et al (2019)	arboret um	walking	State Trait Anxiety Inventory, Positive and Negative Affect Scale , Backward Digit Span	Ctr Gr: 23.00	49.70	Male %: 17.39	150.00	Interval	yes	Affect, Anxiety, Cognition

				Tx Gr: 23.00		Female %: 82.61				
Furuyashiki et al (2019)	forest	Forest bathing	Systolic Blood Pressure, Diastolic Blood Pressure, Heart Rate, Profile of Mood States, Parasympathetic nerve activity, Sympathetic activity	Ctr Gr: n/a Tx Gr: 155.00	44.00	Male %: 44.50 Female %: 55.50	120.00	Continuous	no	Mood, Physiological
Hayashi et al (2008)	garden	Horticultural activity	Profile of Mood States	Ctr Gr:20.00 Tx Gr: 20.00 Ctr Gr:n/a Tx Gr: 11.00	46.00	Male %: 35.00 Female %: 65.00 Male %: 36.00 Female %: 64.00	120.00, 360.00	Continuous	Yes, no	Mood
Ibes & Forestell (2020)	Grass field	Sitting and viewing	Profile of Mood States	Ctr Gr:15.00 Tx Gr: 17.00 Ctr Gr:22.00 Tx Gr: 32.00 Ctr Gr:37.00 Tx Gr: 35.00 Ctr Gr:37.00 Tx Gr: 39.00	19.22	Male %: 57.40 Female %: 42.60	20.00	Continuous	Yes	Mood
Janeczko et al (2020)	forest	walking	Systolic Blood Pressure, Diastolic Blood Pressure, Heart Rate, Positive and Negative Affect Scale, Restorative Outcome Scale, Subjective Vitality Scale, Profile of Mood States	Ctr Gr: 23.00 Tx Gr: 17.00 Ctr Gr: 22.00 Tx Gr: 13.00	n/a	n/a	30.00	Continuous	yes	Quality of Life, Mood, Affect, Physiological
Kim et al (2020)	forest	Forest therapy	Profile of Mood States, Stress Response Inventory – Modified Form	Ctr Gr: 19.00 Tx Gr: 19.00	22.00	Male %: 63.00 Female %: 37.00	720.00	Interval	yes	Mood, Stress

Kjellgren & Buhrkall (2010)	Woodland natural area	Sitting and viewing	Heart Rate, Systolic Blood Pressure, Diastolic Blood Pressure, Stress-VAS	Ctr Gr: 18.00 Tx Gr: 18.00	36.83	Male %: 22.00 Female %: 78.00	30.00	Continuous	yes	Physiological, Stress, Cognition
Klaperski et al (2019)	Multiple natural environments	Running, football, mountain biking	Multidimensional Mood State Questionnaire Calmness, Multidimensional Mood State Questionnaire Mood, Perceived Stress Scale, State Trait Anxiety Inventory	Ctr Gr: 90.00 Tx Gr: 50.00	24.40	Male %: 51.43 Female %: 48.57	105.00	Continuous	yes	Mood, Stress, Negative Emotions
Lachance & Nisbet (2020)	River bank with natural vegetation	Stationary exercise bike, surfing, group processing	Positive and Negative Affect Scale, Neutral Word Rating Scale	Ctr Gr: n/a Tx Gr: 81.00 Ctr Gr: n/a Tx Gr: 83.00	19.78	Male %: 19.50 Female %: 80.50	40.00	Continuous	no	Cognition, Affect
Lee et al (2011)	Forest	Sitting and viewing	Heart Rate, Somatic Differential Comfortable, Stress Arousal Checklist	Ctr Gr: 12.00 Tx Gr: 12.00	21.20	Male %: 100.00 Female %: 0.00	15.00	Continuous	yes	Cognition
Lee et al (2014)	Forest	Walking	Natural log of high frequency, LF/HF ratio, Profile of Mood States	Ctr Gr: 46.00 Tx Gr: 46.00	21.10	Male %: 100.00Female %: 0.00	28.50	Continuous	yes	Physiological, Mood
Lee et al (2020)	Forest	Art psychotherapy	SCL-90-R: Depression, SCL-90-R Anxiety, SCL-90-R Interpersonal Sensitivity, SCL-90-R Self-Regulation problem, SCL-90-R Aggression	Ctr Gr: 8.00 Tx Gr: 8.00	41.69	Male %: 31.25 Female %: 68.75	1120.00	Interval	yes	Negative Emotions, Social Functioning
Mackay et al (2010)	Multiple natural environments	Green exercise	State Trait Anxiety Inventory	Ctr Gr: n/a Tx Gr: 89.00	43.83	Male %: 59.00 Female %: 41.00	91.56	Continuous	yes	Anxiety
Morita et al (2007)	Forest	Shinrin-yoku	MMS-SF Hostility, MMS-SF Depression, MMS-SF Boredom, MMS-SF Friendliness, MMS-SF Wellbeing, MMS-SF Liveliness, State Trait Anxiety Inventory	Ctr Gr: 498.00 Tx Gr: 498.00	56.20	Male %: 49.00 Female %: 51.00	140.00	Continuous	Yes	Social Functioning, Negative Emotions, Anxiety, Depression,

										Mood, Quality of Life
Ochiai et al (2015)	Forest	Forest therapy	Systolic Blood Pressure, Diastolic Blood Pressure, Urinary Adrenaline, Cortisol, Semantic Differential: Comfortable, Semantic Differential: Relaxed, Semantic Differential: Natural, Profile of Mood States	Ctr Gr: n/a Tx Gr: 9.00	56.00	Male %: 100.00 Female %: 0.00	168.00	Continuous	No	Quality of Life, Mood, Stress, Physiological, Environmental Connection
Olafsdottir et al (2020)	Woodland natural area	Walking	Positive and Negative Affect Scale	Ctr Gr: 20.00 Tx Gr: 24.00	24.39	Male %: 29.55 Female %: 70.45	40.00	Continuous	Yes	Affect
Pretty et al (2007)	Multiple natural environments	Walking, cycling, conservation, horse riding, boating, woodland activities, fishing	Rosenberg Self Esteem Scale, Profile of Mood States	Ctr Gr: n/a Tx Gr: 263.00	47.80	Male %: 51.00 Female %: 49.00	267.00	Continuous	No	Mood, Quality of Life
Richardson (2003)	Urban nature	Walking	Efficacy for coping with anxiety, Efficacy for solving problem in a group, Profile of Mood States Physical Self Efficacy Scale, Rosenberg Self Esteem Scale, State Trait Anxiety Inventory, Adolescent Social Self Efficacy Scale, Beck Depression Inventory, Brief Symptom Inventory – 53, General Self Efficacy Scale	Ctr Gr: 11.00 Tx Gr: 11.00 Ctr Gr: 9.00 Tx Gr: 10.00	No data available	Male %: 36.70 Female %: 63.30, Male %: 42.10 Female %: 57.90	270.00	Continuous	Yes	Quality of Life, Mood, Depression, Anxiety, Negative Emotions
Rogerson et al (2016)	Grass field	Stationary exercise bike	Profile of Mood States, Backward Digit Span	Ctr Gr: 24.00 Tx Gr: 24.00	35.10	Male %: 20.80 Female %: 79.20	15.00	Continuous	Yes	Mood, Cognition
Rogerson et al (2020)	Grass field	Running	Connectedness to Nature Scale, Rosenberg Self-Efficacy Scale, Profile of Mood States	Ctr Gr: n/a Tx Gr: 40.00	36.43	Male %: 50.00 Female %: 50.00	15.48	Continuous	No	Environmental Connection, Quality of Life

Saadi et al (2020)	Urban Park	Sitting and viewing	Positive and Negative Affect Scale, Perceived Restorative Scale Being Away, Perceived Restorative Scale Fascination, Perceived Restorative Scale Compatibility, Perceived Restorative Scale, Social Discomfort, LFHF Ratio, HRV SDNN, HRV RMSSD, Backward Digit Span	Ctr Gr: n/a Tx Gr: 72.00	30.04	Male %: 0.00 Female %: 100.00	45.00	Continuous	No	Social Functioning, Negative Emotions, Physiological, Cognition, Affect, Mood, Quality of Life
Tsunetsugu et al (2011)	Forest	Sitting and viewing	Profile of Mood States	Ctr Gr: 219.00 Tx Gr: 219.00	No data available	Male %: 100.00 Female %: 0.00	15.00	Continuous	Yes	Mood
Tyrvainen et al (2014)	Urban forest	Sitting and viewing	Restorative Outcome Scale, Subjective Vitality Scale, PANAS Positive, PANAS Negative	Ctr Gr: 77.00 Tx Gr: 77.00	47.64	Male %: 7.79 Female %: 92.21	52.00	Continuous	Yes	Affect, Quality of Life
Van Den Berg & Custers (2011)	Countryside	Green exercise	Cortisol, Positive and Negative Affect Scale,	Ctr Gr: 16.00 Tx Gr: 14.00	57.60	Male %: 26.67 Female %: 73.33	30.00	Continuous	Yes	Affect, Stress
Yu & Hsieh (2020)	Forest	Forest therapy	Heart Rate, Systolic Blood Pressure, Diastolic Blood Pressure, Profile of Mood States, Chinese Word Remote Associations Test.	Ctr Gr: n/a Tx Gr: 22.00	52.00	Male %: 34.80 Female %: 65.20	240.00	Interval	No	Physiological, Cognition, Mood
Yu et al (2017)	Forest	Forest Bathing	Heart Rate, Systolic Blood Pressure, Diastolic Blood Pressure, High Frequency, LF/HF Ratio, Profile of Mood States, State Trait Anxiety Inventory	Ctr Gr: n/a Tx Gr: 128.00	60.00	Male %: 22.60 Female %: 66.40	120.00	Continuous	No	Mood, Anxiety, Physiological
Zebrack et al. (2017)	Wilderness	Outdoor Adventure Program	PHQ-4, Cancer Behavior Inventory-Brief, Duke-UNC Functional Social Support Questionnaire	Ctr Gr: n/a Tx Gr: 196.00	No data available	Male %: 18.10 Female %: 81.90	10080.00	Continuous	No	Social Functioning, Negative Emotions, Quality of Life
