# Supplementary material for: A Systematic Review and Meta-Analysis on the Effect of Nature Exposure Dose on Adults with Mental Illness
Source: Behav Sci (Basel). 2025 Jan 31;15(2):153. doi: 10.3390/bs15020153 (PMC11851813; doi:10.3390/bs15020153)
Supplement: Supplementary file 1 [file behavsci-15-00153-s001.zip › Supplementary D for dosage-BibliographyExludedStudiesWithReasons.pdf]

Supplementary D: Bibliography of Excluded Studies with Reasons

1. Case study. Measuring the impact of wilderness therapy. *Joint Commission Benchmark*. 2004;6(6):8-10.
Exclusion reason: No data collected that clearly defined nature dosage.

2. Nature's calming influence. *Health Facil Manage*. 2010;23(7):44.
Exclusion reason: Quantitative data not collected (only qualitative data collected) or no original data collected.

3. Beyond Blue to Green: The benefits of contact with nature for mental health and well-being. *Australasian Parks & Leisure*. 2010;13(3):10-11.
Exclusion reason: Quantitative data not collected (only qualitative data collected) or no original data collected.

4. The Effects of Regular Mountain Hiking on Hopelessness in Chronically Suicidal Patients. Trial registry record; Clinical trial protocol. Source: https://clinicaltrials.gov/show/NCT01152086. Cochrane Central Register of Controlled Trials (CENTRAL) [CN-01530260] https://www.cochranelibrary.com/central/doi/10.1002/central/CN-01530260/full.
Exclusion reason: Quantitative data not collected (only qualitative data collected) or no original data collected.

5. Intervention study of psychotherapy. Trial registry record; Clinical trial protocol. Source: http://www.who.int/trialsearch/Trial2.aspx?TrialID=JPRN-UMIN000006170. Cochrane Central Register of Controlled Trials (CENTRAL) [CN-01835996] https://www.cochranelibrary.com/central/doi/10.1002/central/CN-01835996/full.
Exclusion reason: Quantitative data not collected (only qualitative data collected) or no original data collected.

6. 'Green' Exercise for Good Mental Health. *Hoofbeats: An Australian Riding, Training & Horse Care Magazine*. 2013;35(3):47-47.
Exclusion reason: Quantitative data not collected (only qualitative data collected) or no original data collected.

7. Green-fingered therapy is showing results. *Nurs Stand*. 2013;27(52):5-5.
Exclusion reason: Quantitative data not collected (only qualitative data collected) or no original data collected.

8. Wilderness Recreation Experience Boosts Veterans Mental Well-Being. *J Psychosoc Nurs Ment Health Serv*. 2013;51(11):6-13.
Exclusion reason: Quantitative data not collected (only qualitative data collected) or no original data collected.

9. Nacadia Effect Study (NEST). Trial registry record; Clinical trial protocol. Source: https://clinicaltrials.gov/show/NCT01849718. Cochrane Central Register of Controlled Trials (CENTRAL) [CN-01585340] https://www.cochranelibrary.com/central/doi/10.1002/central/CN-01585340/full.
Exclusion reason: quantitative data not collected (only qualitative data collected); no original data collected; or, data reported NOT usable (i.e. no Means/SD).

10. Effect of Group Hiking in Nature on Physical Health and Quality of Life in Chronic Schizophrenic Patients. Trial registry record; Clinical trial protocol. Source: http://www.who.int/trialsearch/Trial2.aspx?TrialID=IRCT201306222085N12. Cochrane Central Register of Controlled Trials (CENTRAL) [CN-01818208] Accessed 2024. https://www.cochranelibrary.com/central/doi/10.1002/central/CN-01818208/full.
Exclusion reason: quantitative data not collected (only qualitative data collected); no original data collected; or, data reported NOT usable (i.e. no Means/SD).

11. Erratum to Correction: Using nature-based rehabilitation to restart a stalled process of rehabilitation in individuals with stress-related mental illness (Int. J. Environ. Res. Public health, (2015), 12, (1928-1951)). *Int J Environ Res Public Health*. 2015;12(6):6946-6947. doi:10.3390/ijerph120606946.
Exclusion reason: quantitative data not collected (only qualitative data collected); no original data collected; or, data reported NOT usable (i.e. no Means/SD).

12. Care home's tool shed lures male residents outdoors. *Mental Health Practice*. 2015;18(8):7-7. doi:10.7748/mhp.18.8.7.s8.
Exclusion reason: Quantitative data not collected (only qualitative data collected) or no original data collected.

13. A long walk through nature: strolling for mental health. *Aging Today*. 2015;36(5):15-15.
Exclusion reason: Quantitative data not collected (only qualitative data collected) or no original data collected.

14. PHENOTYPE. Therapeutic Study in Barcelona. Trial registry record; Clinical trial protocol. Source: https://clinicaltrials.gov/show/NCT02624921. Cochrane Central Register of Controlled Trials (CENTRAL) [CN-02040257] https://www.cochranelibrary.com/central/doi/10.1002/central/CN-02040257/full.
Exclusion reason: quantitative data not collected (only qualitative data collected); no original data collected; or, data reported NOT usable (i.e. no Means/SD).

15. Effects of Horticultural Therapy on Asian Elderly' Mental Health. Trial registry record; Clinical trial protocol. Source: https://clinicaltrials.gov/show/NCT02495194. Cochrane Central Register of Controlled Trials (CENTRAL) [CN-02031796] https://www.cochranelibrary.com/central/doi/10.1002/central/CN-02031796/full.
Exclusion reason: quantitative data not collected (only qualitative data collected); no original data collected; or, data reported NOT usable (i.e. no Means/SD).

16. Study for Decreasing Depressive Symptoms and Increasing Memory Performance in the Elderly. Trial registry record; Clinical trial protocol. Source: http://www.who.int/trialsearch/Trial2.aspx?TrialID=JPRN-UMIN000018547. Cochrane Central Register of Controlled Trials (CENTRAL) [CN-01880926] https://www.cochranelibrary.com/central/doi/10.1002/central/CN-01880926/full.

Exclusion reason: Quantitative data not collected (only qualitative data collected) or no original data collected.

17. More time outside can lift mood in older people. *Nurs Stand*. 2017;31(46):17. doi:10.7748/ns.31.46.17.s20.
Exclusion reason: Quantitative data not collected (only qualitative data collected) or no original data collected.

18. Poor outdoor access linked to depression. *Nurs Older People*. 2017;29(6):6. doi:10.7748/nop.29.6.6.s3.
Exclusion reason: Quantitative data not collected (only qualitative data collected) or no original data collected.

19. Effect of Sunlight Exposure and Outdoor Activities on Depression, Cognition and Quality of Life in the Elderly. Trial registry record; Clinical trial protocol. Source: https://clinicaltrials.gov/show/NCT03304301. Cochrane Central Register of Controlled Trials (CENTRAL) [CN-01564688] https://www.cochranelibrary.com/central/doi/10.1002/central/CN-01564688/full.
Exclusion reason: quantitative data not collected (only qualitative data collected); no original data collected; or, data reported NOT usable (i.e. no Means/SD).

20. Effect of horticultural therapy on patients with depression. Trial registry record; Clinical trial protocol. Source: http://www.who.int/trialsearch/Trial2.aspx?TrialID=JPRN-UMIN000028234. Cochrane Central Register of Controlled Trials (CENTRAL) [CN-01890042] https://www.cochranelibrary.com/central/doi/10.1002/central/CN-01890042/full.
Exclusion reason: Quantitative data not collected (only qualitative data collected) or no original data collected.

21. Effect verification of reminiscence method in the miniature garden. Trial registry record; Clinical trial protocol. Source: http://www.who.int/trialsearch/Trial2.aspx?TrialID=JPRN-UMIN000029749. Cochrane Central Register of Controlled Trials (CENTRAL) [CN-01891175] https://www.cochranelibrary.com/central/doi/10.1002/central/CN-01891175/full.
Exclusion reason: quantitative data not collected (only qualitative data collected); no original data collected; or, data reported NOT usable (i.e. no Means/SD).

22. The effect of a horticultural activities program for the community elderly. Trial registry record; Clinical trial protocol. Source: http://www.who.int/trialsearch/Trial2.aspx?TrialID=JPRN-UMIN000027071. Cochrane Central Register of Controlled Trials (CENTRAL) [CN-01889199] https://www.cochranelibrary.com/central/doi/10.1002/central/CN-01889199/full.
Exclusion reason: Quantitative data not collected (only qualitative data collected) or no original data collected.

23. Sour mood getting you down? Get back to nature: Research suggests that mood disorders can be lifted by spending more time outdoors. *Harv Mens Health Watch*. 2018;22(12):3-3.
Exclusion reason: Quantitative data not collected (only qualitative data collected) or no original data collected.

24. Effectiveness of Nature Walks in Depressed Adults. Trial registry record; Clinical trial protocol. Source: https://clinicaltrials.gov/show/NCT03996785. Cochrane Central Register of Controlled Trials

(CENTRAL) [CN-01983319] Accessed 2024.
https://www.cochranelibrary.com/central/doi/10.1002/central/CN-01983319/full.
Exclusion reason: No data presented on participants' symptoms of mental illness

25. The Nature Cure. *Consumer Reports on Health*. 2019;31(4):3-3.
Exclusion reason: Quantitative data not collected (only qualitative data collected) or no original data collected.

26. Walking Green: the Effects of Walking in Forested and Urban Areas. Trial registry record; Clinical trial protocol. Source: https://clinicaltrials.gov/show/NCT03950661. Cochrane Central Register of Controlled Trials (CENTRAL) [CN-01944794]
https://www.cochranelibrary.com/central/doi/10.1002/central/CN-01944794/full.
Exclusion reason: quantitative data not collected (only qualitative data collected); no original data collected; or, data reported NOT usable (i.e. no Means/SD).

27. The Sunlight Exposure Therapy on the Improvement of Depression and Quality of Life in Post-stroke Patients. Trial registry record; Clinical trial protocol. Source: https://clinicaltrials.gov/show/NCT04036565. Cochrane Central Register of Controlled Trials (CENTRAL) [CN-01983456] https://www.cochranelibrary.com/central/doi/10.1002/central/CN-01983456/full.
Exclusion reason: quantitative data not collected (only qualitative data collected); no original data collected; or, data reported NOT usable (i.e. no Means/SD).

28. Shinrin-Yoku (Forest Bathing) on Psychological Well-being. Trial registry record; Clinical trial protocol. Source: https://clinicaltrials.gov/show/NCT04139421. Cochrane Central Register of Controlled Trials (CENTRAL) [CN-01994377] https://www.cochranelibrary.com/central/doi/10.1002/central/CN-01994377/full.
Exclusion reason: quantitative data not collected (only qualitative data collected); no original data collected; or, data reported NOT usable (i.e. no Means/SD).

29. Veterans Nature Therapy (Vet Hike). Trial registry record; Clinical trial protocol. Source: https://clinicaltrials.gov/show/NCT03997344. Cochrane Central Register of Controlled Trials (CENTRAL) [CN-01946089] https://www.cochranelibrary.com/central/doi/10.1002/central/CN-01946089/full.
Exclusion reason: quantitative data not collected (only qualitative data collected); no original data collected; or, data reported NOT usable (i.e. no Means/SD).

30. Multi-component cognitive intervention with physical exercise for older adults with Alzheimer's disease: a 6-month randomized controlled trial. Trial registry record; Clinical trial protocol. Source: http://www.who.int/trialsearch/Trial2.aspx?TrialID=JPRN-UMIN000036888. Cochrane Central Register of Controlled Trials (CENTRAL) [CN-01969613]
https://www.cochranelibrary.com/central/doi/10.1002/central/CN-01969613/full.
Exclusion reason: Quantitative data not collected (only qualitative data collected) or no original data collected.

31. In Case You Haven't Heard. *Mental Health Weekly*. 2020;30(21):8-8. doi:10.1002/mhw.32382.
Exclusion reason: Wrong study design.

32. The psychological benefits of gardening. *Therapy Today*. 2020;31(5):17-17.

Exclusion reason: Quantitative data not collected (only qualitative data collected) or no original data collected.

33. Abraham TH, Koenig CJ, Zamora K, et al. Situating mental health work in place: Qualitative findings from interviews with Veterans in Southeastern Louisiana and Northern California. *Health & Place*. 2017;47:63-70. doi:10.1016/j.healthplace.2017.07.001.
Exclusion reason: Quantitative data not collected (only qualitative data collected) or no original data collected.

34. Adams M, Morgan J. Mental health recovery and nature: How social and personal dynamics are important. *Ecopsychology*. 2018;10(1):44-52. doi:10.1089/eco.2017.0032.
Exclusion reason: Wrong study design.

35. Adamsson M, Laike T, Morita T. Seasonal Variation in Bright Daylight Exposure, Mood and Behavior among a Group of Office Workers in Sweden. *Journal of Circadian Rhythms*. 2018;16:2. doi:10.5334/jcr.153.
Exclusion reason: Intervention did not occur outdoors.

36. Adevi AA, Uvnäs-Moberg K, Grahn P. Therapeutic interventions in a rehabilitation garden may induce temporary extrovert and/or introvert behavioural changes in patients, suffering from stress-related disorders. *Urban Forestry and Urban Greening*. 2018;30:182-193. doi:10.1016/j.ufug.2018.02.010.
Exclusion reason: Quantitative data not collected (only qualitative data collected) or no original data collected.

37. Aerts R, Honnay O, Van Nieuwenhuyse A. Biodiversity and human health: mechanisms and evidence of the positive health effects of diversity in nature and green spaces. *Br Med Bull*. 2018;127(1):5-22. doi:10.1093/bmb/ldy021.
Exclusion reason: Quantitative data not collected (only qualitative data collected) or no original data collected.

38. Alaimo K, Beavers AW, Crawford C, Snyder EH, Litt JS. Amplifying Health Through Community Gardens: A Framework for Advancing Multicomponent, Behaviorally Based Neighborhood Interventions. *Current Environmental Health Reports*. 2016;3(3):302-12. doi:10.1007/s40572-016-0105-0.
Exclusion reason: quantitative data not collected (only qualitative data collected); no original data collected; or, data reported NOT usable (i.e. no Means/SD).

39. Alcock I, White MP, Wheeler BW, Fleming LE, Depledge MH. Longitudinal effects on mental health of moving to greener and less green urban areas. *Environ Sci Technol*. 2014;48(2):1247-55. doi:10.1021/es403688w.
Exclusion reason: No data collected that clearly defined nature dosage.

40. Alford S, Perlman D, Sumskis S, et al. What can leisure offer those with a mental illness; diversion, experience or something much richer? *World Leisure Journal*. 2017;59(3):218-226.
Exclusion reason: No data collected that clearly defined nature dosage.

41. Aliyas Z. Physical, mental, and physiological health benefits of green and blue outdoor spaces among elderly people. *Int J Environ Health Res*. 2019:1-12. doi:10.1080/09603123.2019.1681379.

Exclusion reason: Only one time point collected.

42. Aliyas Z, Masoudi Nezhad S. The Role of Historical Persian Gardens as Urban Green Spaces: Psychological, Physical, and Social Aspects. *Environmental Justice*. 2019;12(3):132-139. doi:10.1089/env.2018.0034.
Exclusion reason: No data collected on symptoms of mental illness.

43. Allen D. A breath of fresh air. *Mental Health Practice*. 2008;11(10):8-9.
Exclusion reason: Quantitative data not collected (only qualitative data collected) or no original data collected.

44. Alton C, Colacci N, Gatt K, Hitzig SL. The benefits of a SCRP therapeutic recreation cottage program on psychological well-being in persons with spinal cord injury. *Topics in Spinal Cord Injury Rehabilitation*. 2011;16:67. doi:10.1310/sci16S1-53.
Exclusion reason: No data collected on symptoms of mental illness.

45. Alves SM. *The role of nature -related activities in the psychological well -being of nursing home residents*. The University of Wisconsin, Milwaukee; 2003.
Exclusion reason: Intervention did not occur outdoors or intervention does not meet study's definition of nature.

46. Ambrey CL. An investigation into the synergistic wellbeing benefits of greenspace and physical activity: Moving beyond the mean. *Urban Forestry and Urban Greening*. 2016;19:7-12. doi:10.1016/j.ufug.2016.06.020.
Exclusion reason: No data collected that clearly defined nature dosage.

47. Ambrey CL, Cartlidge N. Do the psychological benefits of greenspace depend on one's personality? *Pers Individ Dif*. 2017;116:233-239. doi:10.1016/j.paid.2017.05.001.
Exclusion reason: No data collected that clearly defined nature dosage.

48. An M, Colarelli SM, O'Brien K, Boyajian ME. Why we need more nature at work: Effects of natural elements and sunlight on employee mental health and work attitudes. *PLoS One*. 2016;11(5).
Exclusion reason: Intervention did not occur outdoors or intervention does not meet study's definition of nature.

49. Andel R, Crowe M, Pedersen NL, Fratiglioni L, Johansson B, Gatz M. Physical exercise at midlife and risk of dementia three decades later: a population-based study of Swedish twins. *Journals of Gerontology Series A-Biological Sciences & Medical Sciences*. 2008;63(1):62-6.
Exclusion reason: Intervention did not occur outdoors or intervention does not meet study's definition of nature.

50. Anderson CL, Monroy M, Keltner D. Awe in nature heals: Evidence from military veterans, at-risk youth, and college students. *Emotion*. 2018;18(8):1195-1202. doi:10.1037/emo0000442.
Exclusion reason: No data presented on participants' symptoms of mental illness.

51. Anderson K, Bird M, Macpherson S, McDonough V, Davis T. Findings from a pilot investigation of the effectiveness of a snoezelen room in residential care: should we be engaging with our residents more? *Geriatric Nursing*. 2011;32(3):166-77. doi:10.1016/j.gerinurse.2010.12.011.

Exclusion reason: Intervention did not occur outdoors or intervention does not meet study's definition of nature.

52. Anderson L, Schleien SJ, McAvoy L, Lais G, Seligmann D. Creating positive change through an integrated outdoor adventure program. *Ther Recreation J*. 1997;31(4):214-229.
Exclusion reason: No data collected on symptoms of mental illness.

53. Anthun KS, Maass REK, Hope S, Espnes GA, Bell R, Khan M, Lillefjell M. Addressing inequity: Evaluation of an intervention to improve accessibility and quality of a green space. *Int J Environ Res Public Health*. 2019;16(24)doi:10.3390/ijerph16245015.
Exclusion reason: Did not separate participant group with mental illness or symptoms.

54. Antonioli C, Reveley MA. Randomised controlled trial of animal facilitated therapy with dolphins in the treatment of depression. *BMJ*. 2005;331(7527):1231.
Exclusion reason: Quantitative data not in article, not provided by author.

55. Araci D. *Mindfulness and virtual environments: the impact of mediated nature on anxiety, affect, worry and levels of mindfulness*. University of Southampton; 2018.
Exclusion reason: Intervention did not occur outdoors or intervention does not meet study's definition of nature.

56. Arai A, Ishida K, Tomimori M, Katsumata Y, Grove JS, Tamashiro H. Association between lifestyle activity and depressed mood among home-dwelling older people: a community-based study in Japan. *Aging Ment Health*. 2007;11(5):547-55.
Exclusion reason: No data collected that clearly defined nature dosage.

57. Araújo D, Brymer E, Brito H, Withagen R, Davids K. The empowering variability of affordances of nature: Why do exercisers feel better after performing the same exercise in natural environments than in indoor environments? *Psychol Sport Exerc*. 2019;42:138-145. doi:10.1016/j.psychsport.2018.12.020.
Exclusion reason: quantitative data not collected (only qualitative data collected); no original data collected; or, data reported NOT usable (i.e. no Means/SD).

58. Archer S. Access to nature associated with less stress and lower weight. *IDEA Fitness Journal*. 2009;6(3):71-71.
Exclusion reason: Quantitative data not collected (only qualitative data collected) or no original data collected.

59. Armstrong DL. A community diabetes education and gardening project to improve diabetes care in a Northwest American Indian tribe. *Diabetes Educ*. 2000;26(1):113-20.
Exclusion reason: Quantitative data not collected (only qualitative data collected) or no original data collected.

60. Arnberger A, Eder R, Allex B, et al. Health-Related Effects of Short Stays at Mountain Meadows, a River and an Urban Site-Results from a Field Experiment. *Int J Environ Res Public Health*. 2018;15(12):26. doi:10.3390/ijerph15122647.
Exclusion reason: Did not separate participant group with mental illness or symptoms.

61. Arnold C. Vegetation Delight? Greenness and Reduced Risk of Nonaccidental Death. *Environ Health Perspect*. 2016;124(9):A169-A169. doi:10.1289/ehp.124-A169.
Exclusion reason: Quantitative data not collected (only qualitative data collected) or no original data collected.

62. Aronstein Z, Olsen R, Schulman E. The nursing assistants use of recreational interventions for behavioral management of residents with Alzheimer's disease. *American Journal of Alzheimer's Disease*. 1996:26-31.
Exclusion reason: Intervention did not occur outdoors.

63. Ashbaugh JW. Down the garden path of self-determination. *Ment Retard*. 2002;40(5):416-7.
Exclusion reason: Quantitative data not collected (only qualitative data collected) or no original data collected.

64. Ashman J. Growing Healing One Garden at a Time. *J Christ Nurs*. 2016;33(1):35-7.
Exclusion reason: Quantitative data not collected (only qualitative data collected) or no original data collected.

65. Ashton J. Plants and green spaces provide more than just aesthetic benefits. *Perspectives in Public Health*. 2015;135(4):178-9. doi:10.1177/1757913915586653.
Exclusion reason: Quantitative data not collected (only qualitative data collected) or no original data collected.

66. Aspinall P, Mavros P, Coyne R, Roe J. The urban brain: analysing outdoor physical activity with mobile EEG. *Br J Sports Med*. 2015;49(4):272-6. doi:10.1136/bjsports-2012-091877.
Exclusion reason: No data collected on symptoms of mental illness.

67. Astell-Burt T. Greener neighbourhoods, healthier lives? Evidence from Britain. *J Sci Med Sport*. 2012;15:S100. doi:10.1016/j.jsams.2012.11.242.
Exclusion reason: No data collected that clearly defined nature dosage.

68. Astell-Burt T, Feng X. Association of Urban Green Space With Mental Health and General Health Among Adults in Australia. *JAMA Network Open*. 2019;2(7):e198209. doi:10.1001/jamanetworkopen.2019.8209.
Exclusion reason: No data collected that clearly defined nature dosage.

69. Astell-Burt T, Feng X, Kolt GS. Mental health benefits of neighbourhood green space are stronger among physically active adults in middle-to-older age: evidence from 260,061 Australians. *Prev Med*. 2013;57(5):601-6. doi:10.1016/j.ypmed.2013.08.017.
Exclusion reason: No data collected that clearly defined nature dosage.

70. Astell-Burt T, Mitchell R, Hartig T. The association between green space and mental health varies across the lifecourse. A longitudinal study. *J Epidemiol Community Health*. 2014;68(6):578-83. doi:10.1136/jech-2013-203767.
Exclusion reason: No data collected that clearly defined nature dosage.

71. Attwell C, Johr J, Pincherle A, et al. Neurosensory stimulation outdoors enhances cognition recovery in cognitive motor dissociation: A prospective crossover study. *NeuroRehabilitation*. 2019;44(4):545-554. doi:10.3233/NRE-192692.
Exclusion reason: Only one time point collected.

72. Austin EN, Johnston YAM, Morgan LL. Community Gardening in a Senior Center: A Therapeutic Intervention to Improve the Health of Older Adults. *Ther Recreation J*. 2006;40(1):48-56.
Exclusion reason: Intervention did not occur outdoors or intervention does not meet study's definition of nature.

73. Bae S, Lee S, Lee S, et al. The effect of a multicomponent intervention to promote community activity on cognitive function in older adults with mild cognitive impairment: A randomized controlled trial. *Complement Ther Med*. 2019;42:164-169. doi:10.1016/j.ctim.2018.11.011.
Exclusion reason: Intervention did not occur outdoors or intervention does not meet study's definition of nature.

74. Bailey AW, Allen G, Herndon J, Demastus C. Cognitive benefits of walking in natural versus built environments. *World Leisure Journal*. 2018;60(4):293-305.
Exclusion reason: No data collected on symptoms of mental illness.

75. Bailey AW, Cox G, Liguori G. Exercising in the outdoors: A win-win for communities and individuals. *ACSM's Health and Fitness Journal*. 2018;22(2):4-7. doi:10.1249/FIT.0000000000000365.
Exclusion reason: Quantitative data not collected (only qualitative data collected) or no original data collected.

76. Baird MR. *A horticultural therapy program for the elderly: effects on cognition, quality of life, and loneliness*. Mississippi State University; 2016.
Exclusion reason: Intervention did not occur outdoors.

77. Baklien B, Ytterhus B, Bongaardt R. When everyday life becomes a storm on the horizon: families' experiences of good mental health while hiking in nature. *Anthropol Med*. 2016;23(1):42-53. doi:10.1080/13648470.2015.1056056.
Exclusion reason: Quantitative data not collected (only qualitative data collected) or no original data collected.

78. Ball K, Bauman A, Leslie E, Owen N. Perceived environmental aesthetics and convenience and company are associated with walking for exercise among Australian adults. *Prev Med*. 2001;33(5):434-40.
Exclusion reason: Intervention did not occur outdoors or intervention does not meet study's definition of nature.

79. Ban R. Rehabilitation effect of gardening therapy to treat chronic schizophrenia patients. *Chinese Nursing Research*. 2001;15(6):327-329.
Exclusion reason: Intervention did not occur outdoors or intervention does not meet study's definition of nature.

80. Banay RF, James P, Hart JE, et al. Greenness and Depression Incidence among Older Women. *Environ Health Perspect*. 2019;127(2):27001. doi:10.1289/EHP1229.

Exclusion reason: No data collected that clearly defined nature dosage.

81. Bang KS, Lee I, Kim SJ, Song MK, Park SE. The Effects of Urban Forest-walking Program on Health Promotion Behavior, Physical Health, Depression, and Quality of Life: A Randomized Controlled Trial of Office-workers *Journal of Korean Academy of Nursing*. 2016;46(1):140-148. doi:10.4040/jkan.2016.46.1.140.
Exclusion reason: quantitative data not collected (only qualitative data collected); no original data collected; or, data reported NOT usable (i.e. no Means/SD).

82. Bang KS, Lee IS, Kim SJ, Song MK, Park SE. [The Effects of Urban Forest-walking Program on Health Promotion Behavior, Physical Health, Depression, and Quality of Life: A Randomized Controlled Trial of Office-workers]. *Journal of Korean Academy of Nursing*. 2016;46(1):140-8. doi:10.4040/jkan.2016.46.1.140.
Exclusion reason: quantitative data not collected (only qualitative data collected); no original data collected; or, data reported NOT usable (i.e. no Means/SD).

83. Barclay R, Salbach N, Brooks D, et al. Getting older adults OUTdoors (GO-OUT). *Qual Life Res*. 2016;25(1):166-167. doi:10.1007/s11136-016-1390-7.
Exclusion reason: No data collected on symptoms of mental illness.

84. Barclay R, Webber S, Ripat J, et al. Safety and feasibility of an interactive workshop and facilitated outdoor walking group compared to a workshop alone in increasing outdoor walking activity among older adults: a pilot randomized controlled trial. *Pilot & Feasibility Studies*. 2018;4:179. doi:10.1186/s40814-018-0367-4.
Exclusion reason: Wrong outcomes.

85. Barclay R, Webber S, Ripat J, Tate R. Factors associated with community ambulation and social participation after stroke-data from the canadian longitudinal study on aging. *Int J Stroke*. 2018;13(2):63. doi:10.1177/1747493018789543.
Exclusion reason: No data collected that clearly defined nature dosage.

86. Bartfay E, Stewart P, Bartfay W, Papaconstantinou E. Is There an Association between Physical Activity and Sleep in Community-Dwelling Persons with Dementia: An Exploratory Study Using Self-Reported Measures? *Healthcare*. 2019;7(1):05. doi:10.3390/healthcare7010006.
Exclusion reason: Intervention did not occur outdoors or intervention does not meet study's definition of nature.

87. Baur JWR. *Urban Natural Parks in Portland: Nature, Networks, and Community Health*. Oregon State University; 2011.
Exclusion reason: only one time point collected.

88. Bay-Richter C, Träskman-Bendz L, Grahn P, Brundin L. Garden rehabilitation stabilises INF-gamma and IL-2 levels but does not relieve depressive-symptoms. *Brain Behav Immun*. 2011;25:S217. doi:10.1016/j.bbi.2011.07.135.
Exclusion reason: No data collected that clearly defined nature dosage.

89. Bay-Richter C, Träskman-Bendz L, Grahn P, Brundin L. Garden rehabilitation stabilises INF-gamma and IL-2 levels but does not relieve depressive-symptoms. *Neurology Psychiatry and Brain Research*. 2012;18(2):37. doi:10.1016/j.npbr.2012.02.002.
Exclusion reason: No data collected that clearly defined nature dosage.

90. Beavers AW, Burr K, Li K, et al. Feasibility of a pilot randomized controlled trial to assess health impacts of community gardening. *FASEB J*. 2017;31(1).
Exclusion reason: quantitative data not collected (only qualitative data collected); no original data collected; or, data reported NOT usable (i.e. no Means/SD).

91. Becker F, Kirmess M, Gunther L, Hvistendahl AK. Intensive aphasia therapy-first results of the Sunnaas constraint induced language therapy program. *Neurorehabil Neural Repair*. 2012;26(4):405. doi:10.1177/1545968312447071.
Exclusion reason: Intervention did not occur outdoors.

92. Benbow B. Maximizing the use of outdoor gardens in dementia care facilities. *Canadian Nursing Home*. 2014;25(1):4-13.
Exclusion reason: Quantitative data not collected (only qualitative data collected) or no original data collected.

93. Bennett JL. *An examination of therapeutic recreation programs for Veterans with combat-related disabilities and their families*. Indiana University; 2014.
Exclusion reason: quantitative data not collected (only qualitative data collected); no original data collected; or, data reported NOT usable (i.e. no Means/SD).

94. Bennett JL, Lundberg NR, Zabriskie R, Eggett D. Addressing Posttraumatic Stress Among Iraq and Afghanistan Veterans and Significant Others: An Intervention Utilizing Sport and Recreation. *Ther Recreation J*. 2014;48(1):74-93.
Exclusion reason: No data collected that clearly defined nature dosage.

95. Bennett LW, Cardone S, Jarczyk J. Effects of a therapeutic camping program on addiction recovery. The Algonquin Haymarket Relapse Prevention Program. *J Subst Abuse Treat*. 1998;15(5):469-74.
Exclusion reason: No data collected that clearly defined nature dosage.

96. Berget B, Ekeberg O, Braastad BO. Animal-assisted therapy with farm animals for persons with psychiatric disorders: effects on self-efficacy, coping ability and quality of life, a randomized controlled trial. *Clinical Practice & Epidemiology in Mental Health [Electronic Resource]: CP & EMH*. 2008;4:9. doi:10.1186/1745-0179-4-9.
Exclusion reason: No data collected that clearly defined nature dosage.

97. Beyer KM, Szabo A, Nattinger AB. Time Spent Outdoors, Depressive Symptoms, and Variation by Race and Ethnicity. *Am J Prev Med*. 2016;51(3):281-90. doi:10.1016/j.amepre.2016.05.004.
Exclusion reason: only one time point collected.

98. Bezyak JL, Chan F, Kaya C, Huck G, Chiu C-Y. Validating a Lifestyle Physical Activity Measure for People With Serious Mental Illness. *Rehabilitation Research, Policy & Education*. 2014;28(3):200-204. doi:10.1891/2168-6653.28.3.200.

Exclusion reason: Intervention did not occur outdoors or intervention does not meet study's definition of nature.

99. Bizub AL, Joy A, Davidson L. 'It's like being in another world': Demonstrating the benefits of therapeutic horseback riding for individuals with psychiatric disability. *Psychiatric Rehabilitation Journal*. 2003;26(4):377-384. doi:10.2975/26.2003.377.384.
Exclusion reason: Quantitative data not collected (only qualitative data collected) or no original data collected.

100. Bonsaksen T, Fung KMT, Tsang HWH. Participation in physical activity among inpatients with severe mental illness: a pilot study...including commentary by Fung KMT and Tsang HWH. *International Journal of Therapy & Rehabilitation*. 2011;18(2):91-99. doi:10.12968/ijtr.2011.18.2.91.
Exclusion reason: Intervention did not occur outdoors.

101. Branas CC, Cheney RA, MacDonald JM, Tam VW, Jackson TD, Ten Have TR. A Difference-in-Differences Analysis of Health, Safety, and Greening Vacant Urban Space. *Am J Epidemiol*. 2011;174(11):1296-1306. doi:aje/kwr273.
Exclusion reason: No data collected on symptoms of mental illness.

102. Brass L. Spend TIME IN NATURE. *Vibrant Life*. 2013;29(3):26-29.
Exclusion reason: Quantitative data not collected (only qualitative data collected) or no original data collected.

103. Brewin W. The role of nature in dementia care. *Nursing & Residential Care*. 2016;18(2):101-102. doi:10.12968/nrec.2016.18.2.101.
Exclusion reason: Quantitative data not collected (only qualitative data collected) or no original data collected.

104. Brown AR. Jordan House: a community alternative to hospitalization. A provider organization offers a "natural" environment to people in psychiatric crisis. *Behavioral Healthcare*. 2007;27(7):39-40.
Exclusion reason: Quantitative data not collected (only qualitative data collected) or no original data collected.

105. Brown B, Dybdal L, Noonan C, Pedersen MG, Parker M, Corcoran M. Group Gardening in a Native American Community: A Collaborative Approach. *Health Promotion Practice*. 2020;21(4):611-623. doi:10.1177/1524839919830930.
Exclusion reason: No data collected that clearly defined nature dosage.

106. Brown C. *Implementation of harm reduction activities among a formerly homeless population with chronic alcoholism*. College of St. Scholastica; 2011.
Exclusion reason: No data collected that clearly defined nature dosage.

107. Brown MA, Goldstein-Shirley J, Robinson J, Casey S. The effects of a multi-modal intervention trial of light, exercise, and vitamins on women's mood. *Women Health*. 2001;34(3):93-112.
Exclusion reason: Intervention did not occur outdoors or intervention does not meet study's definition of nature.

108. Bryson J, Feinstein J, Spavor J, Kidd SA. An Examination of the Feasibility of Adventure-Based Therapy in Outpatient Care for Individuals With Psychosis. *Can J Commun Ment Health*. 2013;32(2):1-11. doi:10.7870/cjcmh-2013-015.
Exclusion reason: No data collected that clearly defined nature dosage.

109. Buckley RC, Brough P. Nature, Eco, and Adventure Therapies for Mental Health and Chronic Disease. *Frontiers in Public Health*. 2017;5:220. doi:10.3389/fpubh.2017.00220.
Exclusion reason: Quantitative data not collected (only qualitative data collected) or no original data collected.

110. Buckley RC, Brough P, Westaway D. Bringing Outdoor Therapies Into Mainstream Mental Health. *Frontiers in Public Health*. 2018;6:119. doi:10.3389/fpubh.2018.00119.
Exclusion reason: Quantitative data not collected (only qualitative data collected) or no original data collected.

111. Buettner L, Fitzsimmons S, Labban JD, Barba BE. Active lifestyle for older adults with dementia: Wheelchair biking versus other recreational therapy interventions. *American Journal of Recreation Therapy*. 2013;12(1):31-40. doi:10.5055/ajrt.2013.0038.
Exclusion reason: Correction, need full article.

112. Buettner LL, Fitzsimmons S. AD-venture program: therapeutic biking for the treatment of depression in long-term care residents with dementia. *Am J Alzheimers Dis Other Demen*. 2002;17(2):121-7.
Exclusion reason: Quantitative data not in article, not provided by author.

113. Buru T, Kallay E, Olar LE, Stefan R, Buta E, Cantor M, Papuc I. The evaluation of therapeutic horticulture effects on urinary tryptophan metabolites by using the spectrofluorimetric analysis in a group of students. *J Biotechnol*. 2019;305:S83. doi:10.1016/j.jbiotec.2019.05.288.
Exclusion reason: Intervention did not occur outdoors.

114. Calkins M, Szmerekovsky JG, Biddle S. Effect of increased time spent outdoors on individuals with dementia residing in nursing homes. *Journal of Housing for the Elderly*. 2007;21(3/4):211-228. doi:10.1300/j081v21n03_11.
Exclusion reason: Intervention did not occur outdoors or intervention does not meet study's definition of nature.

115. Canu W, Gordon M, Kuo FE, Taylor AF. Mother nature as treatment for ADHD: overstating the benefits of Green...Kuo FE, Faber Taylor A. A potential natural treatment for attention-deficit/hyperactivity disorder: evidence from a national study. Am J Public Health. 2004:94:1580-1586. *Am J Public Health*. 2005;95(3):371-372. doi:10.2105/AJPH.2004.055962.
Exclusion reason: quantitative data not collected (only qualitative data collected); no original data collected; or, data reported NOT usable (i.e. no Means/SD).

116. Carta MG, Maggiani F, Pilutzu L, et al. Sailing can improve quality of life of people with severe mental disorders: Results of a cross over randomized controlled trial. *Clin Pract Epidemiol Ment Health*. 2014;10(1):80-86.
Exclusion reason: No data collected that clearly defined nature dosage.

117. Cashel CM, Lane SWL, Montgomery D. Emotional response patterns of participants during a wilderness experience. *Wilderness Environ Med*. 1996;7(1):9-18. doi:10.1580/1080-6032(1996)007[0009:ERPOPD]2.3.CO;2.

Exclusion reason: Did not separate participant group with mental illness or symptoms.

118. Chakravorty D, Trunnell EP, Ellis GD. Ropes course participation and post-activity processing on transient depressed mood of hospitalized adult psychiatric patients. *Ther Recreation J*. 1995;29(2):104-113.

Exclusion reason: No data collected that clearly defined nature dosage.

119. Chang B-H, Rong J-R, Lin K-C, Kuo C-J. The Efficacy of Nature Experience Activities on Psychotic Symptoms in Sufferers of Chronic Schizophrenia. *Journal of Nursing & Healthcare Research*. 2011;7(4):305-316.

Exclusion reason: quantitative data not collected (only qualitative data collected); no original data collected; or, data reported NOT usable (i.e. no Means/SD).

120. Chang HT, Wu CD, Su HJ, Jaxa, Korea Aerospace Research I, Engineering ST. Linkage between green space structures and mental disorder in Taiwan. presented at: 40th Asian Conference on Remote Sensing: Progress of Remote Sensing Technology for Smart Future, ACRS 2019; 2020.

Exclusion reason: No data collected that clearly defined nature dosage.

121. Chang P-J, Lin Y, Song R. Leisure satisfaction mediates the relationships between leisure settings, subjective well-being, and depression among middle-aged adults in urban china. *Applied Research in Quality of Life*. 2018;doi:10.1007/s11482-018-9630-3.

Exclusion reason: Only one time point collected.

122. Chang P-J, Song R, Lin Y. Air pollution as a moderator in the association between leisure activities and well-being in urban china. *Journal of Happiness Studies: An Interdisciplinary Forum on Subjective Well-Being*. 2018;doi:10.1007/s10902-018-0055-3.

Exclusion reason: Only one time point collected.

123. Chang PJ, Bae SY. Positive Emotional Effects of Leisure in Green Spaces in Alleviating Work-Family Spillover in Working Mothers. *Int J Environ Res Public Health*. 2017;14(7):11. doi:10.3390/ijerph14070757.

Exclusion reason: quantitative data not collected (only qualitative data collected); no original data collected; or, data reported NOT usable (i.e. no Means/SD).

124. Chang Y, Ewert A, Kamendulis LM, Hocevar BA. Measuring Biophysical and Psychological Stress Levels Following Visitation to Three Locations with Differing Levels of Nature. *Journal of Visualized Experiments*. 2019;148(06):19. doi:10.3791/59272.

Exclusion reason: No data collected on symptoms of mental illness.

125. Chang Y-C, Tzu-hui AT, Chiu S-C. The Relation between Natural Therapy and Physiological at Diagnosis in Breast Cancer...5th Annual Worldwide Nursing Conference (WNC2017), 24th–25th July 2017, Singapore. *Annual Worldwide Nursing Conference*. 2017:237-239. doi:10.5176/2315-4330_WNC17.93.

Exclusion reason: No data collected that clearly defined nature dosage.

126. Chao C-L. *Off-site benefits of recreation to participants: An assessment of psychophysiological effects*. Clemson University; 2004.

Exclusion reason: No data collected on symptoms of mental illness.

127. Chao SF. Outdoor activities and depressive symptoms in displaced older adults following natural disaster: community cohesion as mediator and moderator. *Aging Ment Health*. 2016;20(9):940-7. doi:10.1080/13607863.2015.1044940.

Exclusion reason: Intervention did not occur outdoors or intervention does not meet study's definition of nature.

128. Chen HT, Yu CP, Lee HY. The effects of forest bathing on stress recovery: Evidence from middle-aged females of Taiwan. *Forests*. 2018;8(2)doi:10.3390/f9070403.

Exclusion reason: Did not separate participant group with mental illness or symptoms.

129. Chen LJ, Fox KR, Sun WJ, Tsai PS, Ku PW, Chu D. Associations between walking parameters and subsequent sleep difficulty in older adults: A 2-year follow-up study. *Journal of Sport & Health Science*. 2018;7(1):95-101. doi:10.1016/j.jshs.2017.01.007.

Exclusion reason: No data presented on participants' symptoms of mental illness.

130. Chen Z, He Y, Yu Y. Enhanced functional connectivity properties of human brains during in-situ nature experience. *PeerJ*. 2016;4:e2210. doi:10.7717/peerj.2210.

Exclusion reason: quantitative data not collected (only qualitative data collected); no original data collected; or, data reported NOT usable (i.e. no Means/SD).

131. Christensen KM, Holt JM, Wilson JF. The relationship between outdoor recreation and depression among older adults. *World Leisure Journal*. 2013;55(1):72-82.

Exclusion reason: only one time point collected.

132. Christians HD, Antick JR, Manulik B, Reimann M, Kemalyan NA. Therapeutic benefits of a wilderness kayaking adventure for adult burn survivors. *Journal of Burn Care and Research*. 2015;36:S250. doi:10.1097/BCR.0000000000000251.

Exclusion reason: Quantitative data not collected (only qualitative data collected) or no original data collected.

133. Chu HY, Chen MF, Tsai CC, Chan HS, Wu TL. Efficacy of a horticultural activity program for reducing depression and loneliness in older residents of nursing homes in Taiwan. *Geriatric Nursing*. 2019;40(4):386-391. doi:10.1016/j.gerinurse.2018.12.012.

Exclusion reason: Intervention did not occur outdoors.

134. Clark C, Goering P, Tomlinson G. Challenging expectations: Client perceptions of white water canoeing. *Psychosocial Rehabilitation Journal*. 1991;14(4):71-76. doi:10.1037/h0099412.

Exclusion reason: Quantitative data not collected (only qualitative data collected) or no original data collected.

135. Clark P. Nature and the outdoors: stimulating those with dementia. *Nursing & Residential Care*. 2014;16(6):336-339.

Exclusion reason: Quantitative data not collected (only qualitative data collected) or no original data collected.

136. Cleary A, Roiko A, Burton NW, Fielding KS, Murray Z, Turrell G. Changes in perceptions of urban green space are related to changes in psychological well-being: Cross-sectional and longitudinal study of mid-aged urban residents. *Health & Place*. 2019;59:102201. doi:10.1016/j.healthplace.2019.102201.
Exclusion reason: No data collected that clearly defined nature dosage.

137. Colbert L, Huserik C, Gerber D. The psychological benefits of open water scuba diving in a CNS-impaired population. *J Spinal Cord Med*. 2016;39(5):612-613. doi:10.1080/10790268.2016.1207963.
Exclusion reason: Intervention did not occur outdoors or intervention does not meet study's definition of nature.

138. Collins H, Van Puymbroeck M, Hawkins BL, Vidotto J. The Impact of a Sensory Garden for People with Dementia. *Ther Recreation J*. 2020;54(1):48-63. doi:10.18666/TRJ-2020-V54-I1-10077.
Exclusion reason: Intervention did not occur outdoors or intervention does not meet study's definition of nature.

139. Collins HE. *The Impact of a Sensory Garden Intervention for Individuals with Dementia*. Clemson University; 2019.
Exclusion reason: Intervention did not occur outdoors or intervention does not meet study's definition of nature.

140. Connell BR, Sanford JA, Lewis D. Therapeutic effects of an outdoor activity program on nursing home residents with dementia. *Journal of Housing for the Elderly*. 2007;21(3/4):195-209. doi:10.1300/j081v21n03_10.
Exclusion reason: No data collected that clearly defined nature dosage.

141. Coolman AA, Niedbalski A, Powell DM, Kozlowski CP, Franklin AD, Deem SL. Changes in human health parameters associated with an immersive exhibit experience at a zoological institution. *PLoS One*. 2020;15(4):e0231383. doi:10.1371/journal.pone.0231383.
Exclusion reason: No data presented on participants' symptoms of mental illness.

142. Corazon SS, Nyed PK, Sidenius U, Poulsen DV, Stigsdotter UK. A Long-Term Follow-Up of the Efficacy of Nature-Based Therapy for Adults Suffering from Stress-Related Illnesses on Levels of Healthcare Consumption and Sick-Leave Absence: A Randomized Controlled Trial. *Int J Environ Res Public Health*. 2018;15(1):15. doi:10.3390/ijerph15010137.
Exclusion reason: quantitative data not collected (only qualitative data collected); no original data collected; or, data reported NOT usable (i.e. no Means/SD).

143. Corcoran S. A dementia-friendly walk in the woods. *Nursing & Residential Care*. 2019;21(6):325-327. doi:10.12968/nrec.2019.21.6.325.
Exclusion reason: Quantitative data not collected (only qualitative data collected) or no original data collected.

144. Cordoza M, Ulrich RS, Gariner SK, Manulik BJ, Hazen TM, Fitzpatrick PS, Perkins RSS. Feelings of emotional exhaustion and depersonalization predict self-report of trouble sleeping for nurses working in hospital environments. *Sleep*. 2019;42:A74. doi:10.1093/sleep/zsz067.179.
Exclusion reason: Wrong intervention.

145. Cotton SM, Butselaar FJ. Adventure camps for young adults and adults with mental illness. *Psychiatr Serv*. 2012;63(11):1154. doi:10.1176/appi.ps.1154.
Exclusion reason: No data collected that clearly defined nature dosage.

146. Cox DT, Shanahan DF, Hudson HL, Fuller RA, Anderson K, Hancock S, Gaston KJ. Doses of Nearby Nature Simultaneously Associated with Multiple Health Benefits. *Int J Environ Res Public Health*. 2017;14(2):09. doi:10.3390/ijerph14020172.
Exclusion reason: Did not separate participant group with mental illness or symptoms.

147. Creedon MA. *Connectedness to nature and its relationship to meaning in life*. Massachusetts School of Professional Psychology; 2013.
Exclusion reason: No data collected on symptoms of mental illness.

148. Cuda A. Feeling Blue? Turn to Nature. *Massage Magazine*. 2007;(137):100-102.
Exclusion reason: Quantitative data not collected (only qualitative data collected) or no original data collected.

149. Cumes D. Nature as medicine: the healing power of the wilderness. *Altern Ther Health Med*. 1998;4(2):79-86.
Exclusion reason: Quantitative data not collected (only qualitative data collected) or no original data collected.

150. da Silva SL, Pereira DA, Veloso F, Satler CE, Arantes A, Guimarães RM. Programa de reabilitação neuropsicológica da memória aplicada à demência: Um estudo não controlado intrasujeitos = Neuropsychological rehabilitation of memory applied to dementia: An intra-subject non-controlled study. *Estudos de Psicologia*. 2011;28(2):229-240. doi:10.1590/S0103-166X2011000200010.
Exclusion reason: Intervention did not occur outdoors or intervention does not meet study's definition of nature.

151. Dadvand P, Gidlow C, Kruize H, et al. Positive health effects of the natural outdoor environment in typical populations in different regions in europe (phenotype) project. *Epidemiology*. 2012;23(5):S576. doi:10.1097/01.ede.0000417174.99903.24.
Exclusion reason: No data presented on participants' symptoms of mental illness.

152. Dang DN, Salamun I, Vancauwenberge H, Vanhaudenhuyse A, Debatty H, Corhay JL, Louis R. 2 days at the sea-side! effects on anxiety and depression scores, health locus of control (HLOC) and patient's global impression of change (PGIC) in COPD patients. *Eur Respir J*. 2018;52doi:10.1183/13993003.congress-2018.PA4122.
Exclusion reason: Intervention did not occur outdoors or intervention does not meet study's definition of nature.

153. Davies J, McKenna M, Bayley J, Denner K, Young H. Using engagement in sustainable construction to improve mental health and social connection in disadvantaged and hard to reach groups: a new green care approach. *Journal of Mental Health*. 2020;29(3):350-357. doi:10.1080/09638237.2020.1714001.
Exclusion reason: Intervention did not occur outdoors or intervention does not meet study's definition of nature.

154. Davis C. HOW NATURE CAN BE USED TO CREATE A THERAPEUTIC OUTDOOR ENVIRONMENT. *Nurs Older People*. 2011;23(3):16-19. doi:10.7748/nop2011.04.23.3.16.c8419.
Exclusion reason: Quantitative data not collected (only qualitative data collected) or no original data collected.

155. Davydenko M, Peetz J. Time grows on trees: The effect of nature settings on time perception. *J Environ Psychol*. 2017;54:20-26. doi:10.1016/j.jenvp.2017.09.003.
Exclusion reason: No data presented on participants' symptoms of mental illness.

156. de Boer B, Hamers JPH, Zwakhalen SMG, Tan FES, Verbeek H. Quality of care and quality of life of people with dementia living at green care farms: a cross-sectional study. *BMC Geriatr*. 2017;17(1):155. doi:10.1186/s12877-017-0550-0.
Exclusion reason: No data collected that clearly defined nature dosage.

157. de Bruin S, de Boer B, Beerens H, Buist Y, Verbeek H. Rethinking Dementia Care: The Value of Green Care Farming. *J Am Med Dir Assoc*. 2017;18(3):200-203. doi:10.1016/j.jamda.2016.11.018.
Exclusion reason: Quantitative data not collected (only qualitative data collected) or no original data collected.

158. de Bruin S, Oosting S, Tobi H, Enders-Slegers M-J, van der Zijpp A, Schols J. Comparing day care at green care farms and at regular day care facilities with regard to their effects on functional performance of community-dwelling older people with dementia. *Dementia: The International Journal of Social Research and Practice*. 2012;11(4):503-519. doi:10.1177/1471301211421074.
Exclusion reason: No data collected that clearly defined nature dosage.

159. de Bruin SR, Oosting SJ, Kuin Y, Hoefnagels ECM, Blauw YH, de Groot LCP, Schols JMG. Green care farms promote activity among elderly people with dementia. *Journal of Housing for the Elderly*. 2009;23(4):368-389. doi:10.1080/02763890903327275.
Exclusion reason: No data collected that clearly defined nature dosage.

160. Delfosse C, Lemelin A, Baki AA, Ouellet-Plamondon C. Adventure therapy: a review and a pilot project. *Early intervention in psychiatry*. 2014;8:153. doi:10.1111/eip.12199.
Exclusion reason: Quantitative data not collected (only qualitative data collected) or no original data collected.

161. Detweiler MB, Murphy PF, Kim KY, Myers LC, Ashai A. Scheduled medications and falls in dementia patients utilizing a wander garden. *Am J Alzheimers Dis Other Demen*. 2009;24(4):322-32. doi:10.1177/1533317509334036.
Exclusion reason: No data collected that clearly defined nature dosage.

162. Detweiler MB, Self JA, Lane S, et al. Horticultural therapy: a pilot study on modulating cortisol levels and indices of substance craving, posttraumatic stress disorder, depression, and quality of life in veterans. *Altern Ther Health Med*. 2015;21(4):36-41.
Exclusion reason: Intervention did not occur outdoors.

163. Dewi NS, Komatsuzaki M, Yamakawa Y, et al. Community gardens as health promoters: Effects on mental and physical stress levels in adults with and without mental disabilities. *Sustainability*. 2017;9(1)doi:10.3390/su9010063.

Exclusion reason: Quantitative data not in article, not provided by author.

164. Diaz N. A healing journey for the older adult on the Camino de Santiago. *Int Psychogeriatr*. 2019;31:111-112. doi:10.1017/S1041610219001339.
Exclusion reason: Quantitative data not collected (only qualitative data collected) or no original data collected.

165. Diessner R, Woodward D, Stacy S, Mobasher S. Ten once-a-week brief beauty walks increase appreciation of natural beauty. *Ecopsychology*. 2015;7(3):126-133. doi:10.1089/eco.2015.0001.
Exclusion reason: Wrong patient population.

166. Dixon C. Therapeutic gardens for people with Alzheimer's disease. *Landscapes*. 2005;7(4):16-20.
Exclusion reason: quantitative data not collected (only qualitative data collected); no original data collected; or, data reported NOT usable (i.e. no Means/SD).

167. Dorwart CE. Views From the Path: Evaluating Physical Activity Use Patterns and Design Preferences of Older Adults on the Bolin Creek Greenway Trail. *J Aging Phys Act*. 2015;23(4):513-523.
Exclusion reason: No data presented on participants' symptoms of mental illness.

168. Dowd SB, Davidhizar R. Can mental and physical activities such as chess and gardening help in the prevention and treatment of Alzheimer's? Healthy aging through stimulation of the mind. *J Pract Nurs*. 2003;53(3):11-3.
Exclusion reason: quantitative data not collected (only qualitative data collected); no original data collected; or, data reported NOT usable (i.e. no Means/SD).

169. Dowdall SA. *Roots of the spirit: Interrelationships among ecological actions and attitudes, nature-related exceptional human experiences, spirituality, and well-being*. Institute of Transpersonal Psychology; 1998.
Exclusion reason: No data collected that clearly defined nature dosage.

170. Duggan S, Blackman T, Martyr A, Schaik PV. The impact of early dementia on outdoor life: 'A shrinking world?'. *Dementia: The International Journal of Social Research and Practice*. 2008;7(2):191-204. doi:10.1177/1471301208091158.
Exclusion reason: Quantitative data not collected (only qualitative data collected) or no original data collected.

171. Dustin D, Bricker N, Arave J, Wall W, Wendt G. The promise of river running as a therapeutic medium for veterans coping with post-traumatic stress disorder. *Ther Recreation J*. 2011;45(4):326-340.
Exclusion reason: Quantitative data not collected (only qualitative data collected) or no original data collected.

172. Dybvik JB, Sundsford S, Wang CEA, Nivison M. Significance of nature in a clinical setting and its perceived therapeutic value from patients' perspective. *European Journal of Psychotherapy & Counselling*. 2018;20(4):429-449. doi:10.1080/13642537.2018.1529690.
Exclusion reason: Quantitative data not collected (only qualitative data collected) or no original data collected.

173. Edwards CA, McDonnell C, Merl H. An evaluation of a therapeutic garden's influence on the quality of life of aged care residents with dementia. *Dementia*. 2013;12(4):494-510. doi:10.1177/1471301211435188.

Exclusion reason: Intervention did not occur outdoors or intervention does not meet study's definition of nature.

174. Elsey H. Erratum: Green fingers and clear minds: Prescribing 'care farming' for mental illness (British Journal of General Practice (2016) DOI: 10.3399/bjgp16X683749). *Br J Gen Pract*. 2016;66(648):352. doi:10.3399/bjgp16X685585.

Exclusion reason: Wrong study design.

175. Esots J. Gardening to improve wellbeing. *Aust Nurs J*. 2012;20(1):26.

Exclusion reason: Quantitative data not collected (only qualitative data collected) or no original data collected.

176. Ewert A. Military veterans and the use of Adventure Education experiences in natural environments for therapeutic outcomes. *Ecopsychology*. 2014;6(3):155-164.

Exclusion reason: No data collected on symptoms of mental illness.

177. Farrier A, Baybutt M, Dooris M. Mental health and wellbeing benefits from a prisons horticultural programme. *International journal of prison health*. 2019;15(1):91-104. doi:10.1108/IJPH-11-2017-0055.

Exclusion reason: No data collected that clearly defined nature dosage.

178. Faulkner J, Westrupp N, Rousseau J, Lark S. A randomized controlled trial to assess the effect of self-paced walking on task-specific anxiety in cardiac rehabilitation patients. *J Cardiopulm Rehabil Prev*. 2013;33(5):292-6. doi:10.1097/HCR.0b013e3182a0295c.

Exclusion reason: Intervention did not occur outdoors or intervention does not meet study's definition of nature.

179. Fitzsimmons S, Buettner LL. Therapeutic recreation interventions for need-driven dementia-compromised behaviors in community-dwelling elders. *American Journal of Recreation Therapy*. 2012;11(1):19-33. doi:10.5055/ajrt.2012.0013.

Exclusion reason: Intervention did not occur outdoors or intervention does not meet study's definition of nature.

180. Forsyth A, Lysaght R, Aiken A, Cramm H. Wilderness Adventure Program May Help Combat Perceptions of Stigma Among Veterans. *Ecopsychology*. 2020;12(1):8-18. doi:10.1089/eco.2019.0031.

Exclusion reason: Did not separate participant group with mental illness or symptoms.

181. Fortes C, Mastroeni S, Sperati A, et al. Walking outdoors four times weekly for at least 15 minutes is associated with longevity in a cohort of very elderly people. *Ann Nutr Metab*. 2013;63:1014-1015. doi:10.1159/000354245.

Exclusion reason: No data presented on participants' symptoms of mental illness.

182. Fortes C, Mastroeni S, Sperati A, et al. Walking four times weekly for at least 15 min is associated with longevity in a cohort of very elderly people. *Maturitas*. 2013;74(3):246-51. doi:10.1016/j.maturitas.2012.12.001.

Exclusion reason: No data collected that clearly defined nature dosage.

183. Frühauf A, Niedermeier M, Elliott LR, Ledochowski L, Marksteiner J, Kopp M. Acute effects of outdoor physical activity on affect and psychological well-being in depressed patients - A preliminary study. *Mental Health and Physical Activity*. 2016;10:4-9. doi:10.1016/j.mhpa.2016.02.002.
Exclusion reason: Intervention did not occur outdoors or intervention does not meet study's definition of nature.

184. Fuegen K, Breitenbecher KH. Walking and being outdoors in nature increase positive affect and energy. *Ecopsychology*. 2018;10(1):14-25. doi:10.1089/eco.2017.0036.
Exclusion reason: No data collected on symptoms of mental illness.

185. Fung WK. A study of the effectiveness of a horticultural therapy group on the emotional health of cancer patients. *Psychooncology*. 2015;24:266. doi:10.1002/pon.3874.
Exclusion reason: No data collected that clearly defined nature dosage.

186. Gang MG. Effect of the horticultural intervention program on cognitive function, emotion, communication and behavior in the elderly with Alzheimer's disease. *Int Psychogeriatr*. 2013;25:S140. doi:10.1017/S1041610213002160.
Exclusion reason: Intervention did not occur outdoors or intervention does not meet study's definition of nature.

187. Gao Q, Luan D, Wang X, Xin S, Liu Y, Li J. Effect of sun exposure on cognitive function among elderly individuals in Northeast China. *Clin Interv Aging*. 2018;13:2075-2082. doi:10.2147/CIA.S179355.
Exclusion reason: only one time point collected.

188. Gaysina D, Cooper R, Kuh D, Mishra G. 069 Longitudinal association between early life adversity and physical activity at age 36 years. *J Epidemiol Community Health*. 2010;64:A27-8.
Exclusion reason: No data collected that clearly defined nature dosage.

189. Gerber MM. *Community Gardening: A Novel Intervention for Bhutanese Refugees Living in the USA*. University of North Texas; 2015.
Exclusion reason: No data collected that clearly defined nature dosage.

190. Gerber MM, Callahan JL, Moyer DN, Connally ML, Holtz PM, Janis BM. Nepali Bhutanese refugees reap support through community gardening. *International Perspectives in Psychology: Research, Practice, Consultation*. 2017;6(1):17-31. doi:10.1037/ipp0000061.
Exclusion reason: No data collected that clearly defined nature dosage.

191. Gidlow CJ, Jones MV, Hurst G, et al. Where to put your best foot forward: Psycho-physiological responses to walking in natural and urban environments. *J Environ Psychol*. 2016;45:22-29. doi:10.1016/j.jenvp.2015.11.003.
Exclusion reason: Quantitative data not in article, not provided by author.

192. Gigliotti C, Love-Norris D, Gigliotti S. THERAPEUTICAL ALTERNATIVES FOR PERSONS WITH DEMENTIA: A COMPARISON BETWEEN MUSIC THERAPY AND HORTICULTURE THERAPY IN INSTITUTIONAL CARE PROGRAMS. *The Gerontologist*. 2004;44(1):658.
Exclusion reason: No data collected that clearly defined nature dosage.

193. Gigliotti CM. *A mixed method assessment of horticulture therapy programming for persons with dementia: An exploration and analysis of processes and outcomes*. Virginia Polytechnic Institute and State University; 2007.
Exclusion reason: No data collected that clearly defined nature dosage.

194. Gigliotti CM, Jarrott SE, Yorgason J. Harvesting health: effects of three types of horticultural therapy activities for persons with dementia. *Dementia*. 2004;3(2):161-180.
Exclusion reason: Intervention did not occur outdoors.

195. Glasgow TE, Le HTK, Scott Geller E, Fan Y, Hankey S. How transport modes, the built and natural environments, and activities influence mood: A GPS smartphone app study. *J Environ Psychol*. 2019;66doi:10.1016/j.jenvp.2019.101345.
Exclusion reason: No data collected that clearly defined nature dosage.

196. Glassman SB. *The experience of women discovering wilderness as psychologically healing*. The Union Institute; 1995.
Exclusion reason: quantitative data not collected (only qualitative data collected); no original data collected; or, data reported NOT usable (i.e. no Means/SD).

197. Gonzalez MT, Hartig T, Patil GG, Martinsen EW, Kirkevold M. A prospective study of group cohesiveness in therapeutic horticulture for clinical depression. *Int J Ment Health Nurs*. 2011;20(2):119-29. doi:10.1111/j.1447-0349.2010.00689.x.
Exclusion reason: No data collected that clearly defined nature dosage.

198. Gorski L. Healing through the arts [corrected] [published erratum appears in NURS SPECTRUM (PHILADELPHIA TRI STATE) 2007 Feb 26;16(5):4]. *Nursing Spectrum, Philadelphia Tri-State Edition*. 2006;15(25):10-11.
Exclusion reason: Quantitative data not collected (only qualitative data collected) or no original data collected.

199. Gotink RA, Hermans KS, Geschwind N, De Nooij R, De Groot WT, Speckens AE. Mindfulness and mood stimulate each other in an upward spiral: a mindful walking intervention using experience sampling. *Mindfulness*. 2016;7(5):1114-1122.
Exclusion reason: No data collected that clearly defined nature dosage.

200. Goto S, Gianfagia TJ, Munafo JP, et al. The Power of Traditional Design Techniques: The Effects of Viewing a Japanese Garden on Individuals With Cognitive Impairment. *HERD: Health Environments Research & Design Journal*. 2017;10(4):74-86. doi:10.1177/1937586716680064.
Exclusion reason: Intervention did not occur outdoors.

201. Govea J. *Exercise as treatment for mental illness: A quantitative study*. California State University, Long Beach; 2016.
Exclusion reason: Only one time point collected.

202. Grahn P, Palsdottir AM, Ottosson J, Jonsdottir IH. Longer Nature-Based Rehabilitation May Contribute to a Faster Return to Work in Patients with Reactions to Severe Stress and/or Depression. *Int J Environ Res Public Health*. 2017;14(11):27. doi:10.3390/ijerph14111310.

Exclusion reason: Quantitative data not in article, not provided by author.

203. Granger KV. *Grow Where You Are Planted: The Use of Gardening as Offender Rehabilitation in Prison*. Eastern Kentucky University; 2017.
Exclusion reason: Intervention did not occur outdoors or intervention does not meet study's definition of nature.

204. Granziera S, Alessandri A, Lazzaro A, Zara D, Scarpa A. Nordic Walking and Walking in Parkinson's disease: a randomized single-blind controlled trial. *Aging Clin Exp Res*. 2020;11:11. doi:10.1007/s40520-020-01617-w.
Exclusion reason: Intervention did not occur outdoors or intervention does not meet study's definition of nature.

205. Guan H, Wei H, He X, Ren Z, An B. The tree-species-specific effect of forest bathing on perceived anxiety alleviation of young-adults in urban forests. *Annals of Forest Research*. 2017;60(2):327-341. doi:10.15287/afr.2017.897.
Exclusion reason: No data collected on symptoms of mental illness.

206. Gueib C, Pop A, Bannay A, et al. Impact of a Healing Garden on Self-Consciousness in Patients with Advanced Alzheimer's Disease: An Exploratory Study1. *J Alzheimers Dis*. 2020;75(4):1283-1300. doi:10.3233/JAD-190748.
Exclusion reason: Intervention did not occur outdoors or intervention does not meet study's definition of nature.

207. Hadley JL. *The effects of an outdoor adventure on mood and self-concept*. University of Utah; 1994.
Exclusion reason: Quantitative data not collected (only qualitative data collected) or no original data collected.

208. Hall J, Mitchell G, Webber C, Johnson K. Effect of horticultural therapy on wellbeing among dementia day care programme participants: A mixed-methods study (Innovative Practice). *Dementia*. 2018;17(5):611-620. doi:10.1177/1471301216643847.
Exclusion reason: Quantitative data not collected (only qualitative data collected) or no original data collected.

209. Han AR, Park SA, Ahn BE. Reduced stress and improved physical functional ability in elderly with mental health problems following a horticultural therapy program. *Complement Ther Med*. 2018;38:19-23. doi:10.1016/j.ctim.2018.03.011.
Exclusion reason: No data collected that clearly defined nature dosage.

210. Han JW, Choi H, Jeon YH, Yoon CH, Woo JM, Kim W. The Effects of Forest Therapy on Coping with Chronic Widespread Pain: Physiological and Psychological Differences between Participants in a Forest Therapy Program and a Control Group. *Int J Environ Res Public Health*. 2016;13(3):24. doi:10.3390/ijerph13030255.
Exclusion reason: Correction, need full article.

211. Han KH, Lee SM, Kim HS, Sun JK, th IJ-I, Japanese Society of People-Plant R. A study on interpersonal relations of demented elders through therapeutic horticultural activities of buddy system.

presented at: 8th International People-Plant Symposium on Exploring Therapeutic Powers of Flowers, Greenery and Nature; 2008; Awaji.
Exclusion reason: No data collected that clearly defined nature dosage.

212. Harada K, Lee S, Lee S, Bae S, Harada K, Suzuki T, Shimada H. Objectively-measured outdoor time and physical and psychological function among older adults. *Geriatrics & gerontology international*. 2017;17(10):1455-1462. doi:10.1111/ggi.12895.
Exclusion reason: Intervention did not occur outdoors or intervention does not meet study's definition of nature.

213. Harper NJ. *A mixed methods examination of family involvement in adolescent wilderness therapy*. University of Minnesota; 2007.
Exclusion reason: Wrong patient population.

214. Hart J. Prescribing Nature Therapy for Improved Mental Health. *Alternative and Complementary Therapies*. 2016;22(4):161-163. doi:10.1089/act.2016.29067.jha.
Exclusion reason: Wrong study design.

215. Hartig T. Green space, psychological restoration, and health inequality. *Lancet*. 2008;372(9650):1614-5. doi:10.1016/S0140-6736(08)61669-4.
Exclusion reason: Quantitative data not collected (only qualitative data collected) or no original data collected.

216. Hartwig KA, Mason M. Community Gardens for Refugee and Immigrant Communities as a Means of Health Promotion. *J Community Health*. 2016;41(6):1153-1159.
Exclusion reason: Did not separate participant group with mental illness or symptoms.

217. Hassan A, Qibing C, Tao J. Physiological and psychological effects of gardening activity in older adults. *Geriatrics & gerontology international*. 2018;18(8):1147-1152. doi:10.1111/ggi.13327.
Exclusion reason: Intervention did not occur outdoors or intervention does not meet study's definition of nature.

218. Hassan A, Tao J, Li G, et al. Effects of Walking in Bamboo Forest and City Environments on Brainwave Activity in Young Adults. *Evidence-Based Complementary & Alternative Medicine: eCAM*. 2018;2018:9653857. doi:10.1155/2018/9653857.
Exclusion reason: Wrong patient population.

219. Hawkins JL, Thirlaway KJ, Backx K, Clayton DA. Allotment gardening and other leisure activities for stress reduction and healthy aging. *HortTechnology*. 2011;21(5):577-585.
Exclusion reason: Only one time point collected.

220. Hayes RL, Halford WK, Varghese FN. Generalization of the effects of activity therapy and social skills training on the social behavior of low functioning schizophrenic patients. *Occupational Therapy in Mental Health*. 1991;11(4):3-20. doi:10.1300/J004v11n04_02.
Exclusion reason: Intervention did not occur outdoors or intervention does not meet study's definition of nature.

221. Heaslip V, Vahdaninia M, Hind M, et al. Locating oneself in the past to influence the present: Impacts of Neolithic landscapes on mental health well-being. *Health & Place*. 2020;62:102273. doi:10.1016/j.healthplace.2019.102273.
Exclusion reason: No data collected that clearly defined nature dosage.

222. Heath Y. Evaluating the effect of therapeutic gardens. *Am J Alzheimers Dis Other Demen*. 2004;19(4):239-42.
Exclusion reason: No data collected on symptoms of mental illness.

223. Heilmayr DP. *Cultivating Healthy Trajectories: An Experimental Study of Community Gardening, Personality, and Health Behaviors*. University of California, Riverside; 2017.
Exclusion reason: Wrong patient population.

224. Hewitt P, Watts C, Hussey J, Power K, Williams T. Does a structured gardening programme improve well-being in young-onset dementia? A preliminary study. *The British Journal of Occupational Therapy*. 2013;76(8):355-361. doi:10.4276/030802213X13757040168270.
Exclusion reason: Intervention did not occur outdoors.

225. Hickey B. Lothlorien community: A holistic approach to recovery from mental health problems. *Therapeutic Communities*. 2008;29(3):261-272.
Exclusion reason: Quantitative data not collected (only qualitative data collected) or no original data collected.

226. Hine R, Peacock J, Pretty J. Care farming in the UK: Contexts, benefits and links with therapeutic communities. *Therapeutic Communities*. 2008;29(3):245-260.
Exclusion reason: No data collected that clearly defined nature dosage.

227. Ho RT, Fong TC, Wan AH, et al. A randomized controlled trial on the psychophysiological effects of physical exercise and Tai-chi in patients with chronic schizophrenia. *Schizophr Res*. 2016;171(1-3):42-9. doi:10.1016/j.schres.2016.01.038.
Exclusion reason: Intervention did not occur outdoors or intervention does not meet study's definition of nature.

228. Honeychurch K. *Ecotherapy Adventure and REBGT for Returning Veterans with Disabilities*. Saint Mary's College of California; 2016.
Exclusion reason: Quantitative data not collected (only qualitative data collected) or no original data collected.

229. Hoschlova K, Berguet D. Are trips to high altitude beneficial for psychiatric patients? *High Altitude Medicine and Biology*. 2018;19(4):A408-A409. doi:10.1089/ham.2018.29015.abstracts.
Exclusion reason: No data collected that clearly defined nature dosage.

230. Houlden V, João de Albuquerque P, Weich S, Jarvis S. Does nature make us happier? A spatial error model of greenspace types and mental wellbeing. *Environment and Planning B: Urban Analytics and City Science*. 2019;doi:10.1177/2399808319887395.
Exclusion reason: No data collected on symptoms of mental illness.

231. Howarth M, Rogers M, Withnell N, McQuarrie C. Growing spaces: An evaluation of the mental health recovery programme using mixed methods. *J Res Nurs*. 2018;23(6):476-489. doi:10.1177/1744987118766207.
Exclusion reason: No data collected that clearly defined nature dosage.

232. Huang DZ. *Research of outward bound affecting mental health of university poor freshman : base on the case of poor freshman in gxut*. East China Normal University; 2010.
Exclusion reason: No full text available.

233. Huang WZ, Liu L, Wang ZJ, Mai GH, Cui SY, Yan W. Effect of horticultural therapy on psychological factors of patients with hand functional rehabilitation. *Basic and Clinical Pharmacology and Toxicology*. 2018;122:50. doi:10.1111/bcpt.12968.
Exclusion reason: No data collected that clearly defined nature dosage.

234. Hull RB, Michael SE. Nature-based recreation, mood change, and stress restoration. *Leisure Sciences*. 1995;17(1):1-14. doi:10.1080/01490409509513239.
Exclusion reason: No data collected on symptoms of mental illness.

235. Hurst A. *An exploration of relationships between greenspace, mindfulness, and perseverative thinking in relation to depression*. The University of Liverpool; 2018.
Exclusion reason: only one time point collected.

236. Hutcheson C, Ferguson H, Nish G, Gill L. Promoting mental wellbeing through activity in a mental health hospital. *Br J Occup Ther*. 2010;73(3):121-128. doi:10.4276/030802210X12682330090497.
Exclusion reason: Intervention did not occur outdoors or intervention does not meet study's definition of nature.

237. Huynh T, Torquati JC. Examining connection to nature and mindfulness at promoting psychological well-being. *J Environ Psychol*. 2019;66doi:10.1016/j.jenvp.2019.101370.
Exclusion reason: No data collected that clearly defined nature dosage.

238. Ibsen TL, Eriksen S, Patil GG. Farm-based day care in Norway - a complementary service for people with dementia. *Journal of multidisciplinary healthcare*. 2018;11:349-358. doi:10.2147/JMDH.S167135.
Exclusion reason: Only one time point collected.

239. Ibsen TL, Kirkevold O, Patil GG, Eriksen S. People with dementia attending farm-based day care in Norway - Individual and farm characteristics associated with participants' quality of life. *Health & Social Care in the Community*. 2020;28(3):1038-1048. doi:10.1111/hsc.12937.
Exclusion reason: No data collected that clearly defined nature dosage.

240. Ikei H, Koizumi H, Song C, Kouzuki M, Teratani S, Sakuma T, Miyazaki Y. [Psychological Effects of Forest Therapy Program on Workers]. *Nippon Eiseigaku Zasshi - Japanese Journal of Hygiene*. 2015;70(2):161-6.
Exclusion reason: No Translation Available.

241. Ikei H, Song C, Kagawa T, Miyazaki Y. Physiological and psychological effects of viewing forest landscapes in a seated position in one-day forest therapy experimental model. *Nippon Eiseigaku Zasshi - Japanese Journal of Hygiene*. 2014;69(2):104-10.
Exclusion reason: No translation available.

242. Irandoust K, Taheri M. The Effect of Vitamin D supplement and Indoor Vs Outdoor Physical Activity on Depression of Obese Depressed Women. *Asian J Sports Med*. 2017;8(3):1-7. doi:10.5812/asjsm.13311.
Exclusion reason: Intervention did not occur outdoors or intervention does not meet study's definition of nature.

243. Isaacs PM. *Aloha`Āina : A Hawaiian garden intervention designed to plant the seeds of recovery in persons with severe and persistent mental illness*. University of Hawai'i at Manoa; 2009.
Exclusion reason: Intervention did not occur outdoors or intervention does not meet study's definition of nature.

244. Ismail M, McGovern R, Harmon D. The benefit of green exercise and attention restoration in chronic pain. *Ir J Med Sci*. 2017;186(2):S105. doi:10.1007/s11845-017-1553-8.
Exclusion reason: Quantitative data not collected (only qualitative data collected) or no original data collected.

245. Ito E. *The Effects of Culture on Conceptualizations of Leisure, Control, and Positive Affect Between Japanese and Canadian Undergraduate Students*. University of Alberta; 2014.
Exclusion reason: Intervention did not occur outdoors or intervention does not meet study's definition of nature.

246. Ito E, Walker GJ. Cultural Commonality and Specificity in Japanese and Euro-Canadian Undergraduate Students' Leisure Experiences: An Exploratory Study on Control and Positive Affect. *Leisure Sciences*. 2016;38(3):249-267. doi:10.1080/01490400.2015.1092403.
Exclusion reason: No data collected on symptoms of mental illness.

247. Ito E, Walker GJ. Relationships among self-construal, control, and positive affect in Japanese undergraduate students' leisure experience. *World Leisure Journal*. 2018;60(1):14-28. doi:10.1080/16078055.2017.1278716.
Exclusion reason: Did not separate participant group with mental illness or symptoms.

248. J S, C V. Assisting the transition of ex-military veterans into civilian life...RCOT (Royal College of Occupational Therapist) Annual Conference 2017. *Br J Occup Ther*. 2017;80:56-56.
Exclusion reason: Quantitative data not collected (only qualitative data collected) or no original data collected.

249. Jakubec SL, Carruthers Den Hoed D, Ray H, Krishnamurthy A. Mental well-being and quality-of-life benefits of inclusion in nature for adults with disabilities and their caregivers. *Landscape Research*. 2016;41(6):616-627. doi:10.1080/01426397.2016.1197190.
Exclusion reason: Quantitative data not collected (only qualitative data collected) or no original data collected.

250. Jarrott SE, Gigliotti C. From the garden to the table: Evaluation of a dementia-specific HT program. *Acta Horticulturae*. 2004;639:139-144. doi:10.17660/ActaHortic.2004.639.16.

Exclusion reason: No data collected that clearly defined nature dosage.

251. Jennings V, Osborne Jelks NT, Dills J. Parks and Health Equity: An avenue to support health and wellness for all. *Parks & Recreation*. 2018;53(11):44-45.

Exclusion reason: Quantitative data not collected (only qualitative data collected) or no original data collected.

252. Jessup H, Testad I, Buettner L. Raising memories: Horticulture therapy in Stokka Teaching Nursing Home for residents with dementia. *Activities Directors' Quarterly for Alzheimer's & Other Dementia Patients*. 2011;12(3):41-46.

Exclusion reason: Quantitative data not collected (only qualitative data collected) or no original data collected.

253. Jia B, Yang Z, Mao G, et al. Beneficial effect of forest bathing on elderly patients with chronic obstructive pulmonary disease. *Acta Cytol*. 2016;60:197-198. doi:10.1159/000446388.

Exclusion reason: No data collected on symptoms of mental illness.

254. Jia BB, Yang ZX, Mao GX, et al. Beneficial effect of forest bathing for elderly patients with stable chronic obstructive pulmonary disease. *J Am Geriatr Soc*. 2014;62:S369. doi:10.1111/jgs.13075.

Exclusion reason: No data collected on symptoms of mental illness.

255. Jiang S. *Encouraging engagement with therapeutic landscapes: Using transparent spaces to optimize stress reduction in urban health facilities*. Clemson University; 2016.

Exclusion reason: No data collected that clearly defined nature dosage.

256. Johnsen SAK. Exploring the use of nature for emotion regulation: Associations with personality, perceived stress, and restorative outcomes. *Nordic Psychology*. 2013;65(4):306-321. doi:10.1080/19012276.2013.851445.

Exclusion reason: No data presented on participants' symptoms of mental illness.

257. Johnson EG, Davis EB, Johnson J, Pressley JD, Sawyer S, Spinazzola J. The effectiveness of trauma-informed wilderness therapy with adolescents: A pilot study. *Psychol Trauma*. 2020;04:04. doi:10.1037/tra0000595.

Exclusion reason: Wrong patient population.

258. Johnson U, Ivarsson A, Parker J, Andersen MB, Svetoft I. Connection in the fresh air: A study on the benefits of participation in an electronic tracking outdoor gym exercise programme. *Montenegrin Journal of Sports Science and Medicine*. 2019;8(1):61-67. doi:10.26773/mjssm.190309.

Exclusion reason: Intervention did not occur outdoors or intervention does not meet study's definition of nature.

259. Jordan M. Back to nature. *Therapy Today*. 2009;20(3):26-28.

Exclusion reason: Quantitative data not collected (only qualitative data collected) or no original data collected.

260. Joshi M, Goldman JZ. Endure, evolve, achieve: Stakeholder perspectives on the effectiveness of the Swamp Apes program in restoring biopsychosocial functioning of American veterans. *Cogent Psychology*. 2019;6(1)doi:10.1080/23311908.2019.1584081.

Exclusion reason: Quantitative data not collected (only qualitative data collected) or no original data collected.

261. Joy YS, Lee AY, Park SA. A Horticultural Therapy Program Focused on Succulent Cultivation for the Vocational Rehabilitation Training of Individuals with Intellectual Disabilities. *Int J Environ Res Public Health*. 2020;17(4):18. doi:10.3390/ijerph17041303.

Exclusion reason: Intervention did not occur outdoors or intervention does not meet study's definition of nature.

262. Jung S, Lee S, Bae S, Lee S, Makino K, Shinkai Y, Shimada H. Effectiveness of the KENKOJISEICHI local revitalization system on cognitive function change in older adults with mild cognitive impairment: study protocol for a randomized controlled trial. *Trials*. 2018;19(1):276. doi:10.1186/s13063-018-2642-3.

Exclusion reason: Wrong study design.

263. Kaiseler M, Kay C, McKenna J. The Impact of an Outdoor and Adventure Sports Course on the Wellbeing of Recovering UK Military Personnel: An Exploratory Study. *Sports*. 2019;7(5):15. doi:10.3390/sports7050112.

Exclusion reason: No data collected that clearly defined nature dosage.

264. Kam MCY, Siu AMH. Evaluation of a horticultural activity programme for persons with psychiatric illness. *Hong Kong Journal of Occupational Therapy*. 2010;20(2):80-86. doi:10.1016/S1569-1861(11)70007-9.

Exclusion reason: Quantitative data not in article, not provided by author.

265. Kamitsis I, Francis AJP. Spirituality mediates the relationship between engagement with nature and psychological wellbeing. *J Environ Psychol*. 2013;36:136-143. doi:10.1016/j.jenvp.2013.07.013.

Exclusion reason: No data collected on symptoms of mental illness.

266. Kardjono J, Danuwijaya AA, Abdullah AG, Nandiyanto ABD. Gender Anxiety Control Through the Outdoor Education Program. presented at: 1st Annual Applied Science and Engineering Conference, AASEC 2016, in conjunction with the International Conference on Sport Science, Health, and Physical Education, ICSSHPE 2016; 2017.

Exclusion reason: No data collected that clearly defined nature dosage.

267. Kennedy T. *The effect of exercise environment on trait anxiety and anxiety sensitivity in college students*. Hofstra University; 2016.

Exclusion reason: Intervention did not occur outdoors or intervention does not meet study's definition of nature.

268. Kerr J, Marshall S, Godbole S, et al. The relationship between outdoor activity and health in older adults using GPS. *Int J Environ Res Public Health*. 2012;9(12):4615-25.

Exclusion reason: Intervention did not occur outdoors or intervention does not meet study's definition of nature.

269. Kerr JH, Fujiyama H, Sugano A, Okamura T, Chang M, Onouha F. Psychological responses to exercising in laboratory and natural environments. *Psychol Sport Exerc*. 2006;7(4):345-359. doi:10.1016/j.psychsport.2005.09.002.
Exclusion reason: No data collected on symptoms of mental illness.

270. Kessell MJ. Women's adventure group: Experiential therapy in an HMO setting. *Women Ther*. 1994;15(3-4):185-203. doi:10.1300/J015v15n03_15.
Exclusion reason: quantitative data not collected (only qualitative data collected); no original data collected; or, data reported NOT usable (i.e. no Means/SD).

271. Kidd JL, Brascamp W. Benefits of gardening to the well-being of New Zealand gardeners. *Acta Horticulturae*. 2004;639:103-112. doi:10.17660/ActaHortic.2004.639.12.
Exclusion reason: No data presented on participants' symptoms of mental illness.

272. Kim J, Kim H. Demographic and Environmental Factors Associated with Mental Health: A Cross-Sectional Study. *Int J Environ Res Public Health*. 2017;14(4):17. doi:10.3390/ijerph14040431.
Exclusion reason: No data collected that clearly defined nature dosage.

273. Kim JH, Cho MK, Park HS, Joo SH, Son KC. Effects of horticultural therapy based on social skill on the improvement of interpersonal relationship and sociality of women with mental retardation. *Korean Journal of Horticultural Science and Technology*. 2008;26(1):81-89.
Exclusion reason: No Translation Available.

274. Kim KH, Park SA. Horticultural therapy program for middle-aged women's depression, anxiety, and self-identify. *Complement Ther Med*. 2018;39:154-159. doi:10.1016/j.ctim.2018.06.008.
Exclusion reason: Intervention did not occur outdoors or intervention does not meet study's definition of nature.

275. King JD. Environmental preference might mediate the benefits of nature-based therapies. *Br J Psychiatry*. 2019;214(2):115-116. doi:10.1192/bjp.2018.280.
Exclusion reason: Quantitative data not collected (only qualitative data collected) or no original data collected.

276. Kishore TM, Nagar RK. Mountaineering expedition by persons with intellectual disability: impact on behavior and temperament. *Journal of Intellectual Disabilities*. 2008;12(3):183-189.
Exclusion reason: No data collected on symptoms of mental illness.

277. Kolanowski AM, Buettner L, Costa PT, Jr., Litaker MS. Capturing interests: Therapeutic recreation activities for persons with dementia. *Ther Recreation J*. 2001;35(3):220-235.
Exclusion reason: Intervention did not occur outdoors.

278. Kondo MC, Triguero-Mas M, Donaire-Gonzalez D, et al. Momentary mood response to natural outdoor environments in four European cities. *Environ Int*. 2020;134:105237. doi:10.1016/j.envint.2019.105237.
Exclusion reason: Intervention did not occur outdoors or intervention does not meet study's definition of nature.

279. Kondo T, Takeda A. Physiological and Psychological Effects of Shinrin-yoku in Elderly Persons. *Japanese Journal of Clinical Sports Medicine*. 2009;17(1):122-128.
Exclusion reason: No Translation Available.

280. Kondo T, Takeda A, Takeda N, Shimomura Y, Yatagai M, Kobayashi I. A physio-psychological research on Shinrin-yoku. *Journal of the Japanese Society of Balneology, Climatology & Physical Medicine*. 2008;71(2):131-138.
Exclusion reason: quantitative data not collected (only qualitative data collected); no original data collected; or, data reported NOT usable (i.e. no Means/SD).

281. Korpela K, Savonen EM, Anttila S, Pasanen T, Ratcliffe E. Enhancing wellbeing with psychological tasks along forest trails. *Urban Forestry and Urban Greening*. 2017;26:25-30. doi:10.1016/j.ufug.2017.06.004.
Exclusion reason: No data collected on symptoms of mental illness.

282. Korpela KM, Ylen M, Tyrvainen L, Silvennoinen H. Determinants of restorative experiences in everyday favorite places. *Health & Place*. 2008;14(4):636-52.
Exclusion reason: No data collected on symptoms of mental illness.

283. Koselka EPD, Weidner LC, Minasov A, et al. Walking Green: Developing an Evidence Base for Nature Prescriptions. *Int J Environ Res Public Health*. 2019;16(22):07. doi:10.3390/ijerph16224338.
Exclusion reason: quantitative data not collected (only qualitative data collected); no original data collected; or, data reported NOT usable (i.e. no Means/SD).

284. Koura S, Ikeda A, Rappe E, Park SA. Effects of horticultural therapeutic garden on autonomic nervous system among elderly people with dementia and the value of people-plants relationships. *Acta Horticulturae*. 2016;1121:27-32. doi:10.17660/ActaHortic.2016.1121.5.
Exclusion reason: Quantitative data not collected (only qualitative data collected) or no original data collected.

285. Koura S, Okawa H, Oshikawa T, et al. Dementia protective efficacy by the combination of active and passive horticultural therapy for all persons concerned. *Acta Horticulturae*. 2018;1215:223-231. doi:10.17660/ActaHortic.2018.1215.41.
Exclusion reason: Intervention did not occur outdoors.

286. Kunstler R, Sokoloff S. Clinical effectiveness of intensive therapeutic recreation: A multiple case study of private practice intervention. *Loss, Grief & Care*. 1993;6(4):23-30. doi:10.1300/J132v06n04_04.
Exclusion reason: Quantitative data not collected (only qualitative data collected) or no original data collected.

287. LaChance A. *Effect of Body Size and Exercise on Mood State*. University of Arkansas; 2012.
Exclusion reason: Intervention did not occur outdoors or intervention does not meet study's definition of nature.

288. Laget PL. [Gardens and gardening as curative instrument with regard to mental disorders]. *Revue de la Societe Francaise D'Histoire des Hopitaux*. 2009;(133-134):28-34.
Exclusion reason: quantitative data not collected (only qualitative data collected); no original data collected; or, data reported NOT usable (i.e. no Means/SD).

289. Lammel JA. *Relationship between dimensions of leisure activity experience, sense of coherence, and psychological well -being for traumatic brain injury survivors*. The Pennsylvania State University; 2003.

Exclusion reason: No data collected that clearly defined nature dosage.

290. Largo-Wight E, Chen WW, Dodd V, Weiler R. Healthy workplaces: the effects of nature contact at work on employee stress and health. *Public Health Rep*. 2011;126 Suppl 1:124-30.

Exclusion reason: Intervention did not occur outdoors or intervention does not meet study's definition of nature.

291. Larner AJ. Gardening and dementia. *Int J Geriatr Psychiatry*. 2005;20(8):796-7.

Exclusion reason: Quantitative data not collected (only qualitative data collected) or no original data collected.

292. Lauer CS. Growing evidence: studies show the therapeutic value of healing gardens. *Mod Healthc*. 2008;38(43):24-24.

Exclusion reason: Quantitative data not collected (only qualitative data collected) or no original data collected.

293. Lawton E, Brymer E, Clough P, Denovan A. The Relationship between the Physical Activity Environment, Nature Relatedness, Anxiety, and the Psychological Well-being Benefits of Regular Exercisers. *Front Psychol*. 2017;8:1058. doi:10.3389/fpsyg.2017.01058.

Exclusion reason: only one time point collected.

294. Le Lay B, Breton V, Griner-Abraham V. [When the garden is therapy...]. *Soins*. 2008;Gerontologie.(69):14-7.

Exclusion reason: quantitative data not collected (only qualitative data collected); no original data collected; or, data reported NOT usable (i.e. no Means/SD).

295. Lee KJ, Hur J, Yang K-S, Lee M-K, Lee S-J. Acute biophysical responses and psychological effects of different types of forests in patients with metabolic syndrome. *Environ Behav*. 2018;50(3):298-323. doi:10.1177/0013916517700957.

Exclusion reason: Did not separate participant group with mental illness or symptoms.

296. Lee P-L, Lan W, Lee CCL. Physical activity related to depression and predicted mortality risk: Results from the Americans changing lives study. *Educational Gerontology*. 2012;38(10):678-690. doi:10.1080/03601277.2011.598410.

Exclusion reason: No data collected that clearly defined nature dosage.

297. Lee S, Kim MS, Suh JK, th IJ-I, Japanese Society of People-Plant R. Effects of horticultural therapy of self-esteem and depression of battered women at a Shelter in Korea. presented at: 8th International People-Plant Symposium on Exploring Therapeutic Powers of Flowers, Greenery and Nature; 2008; Awaji.

Exclusion reason: Intervention did not occur outdoors or intervention does not meet study's definition of nature.

298. Lesser IA, Nienhuis CP. The Impact of COVID-19 on Physical Activity Behavior and Well-Being of Canadians. *Int J Environ Res Public Health*. 2020;17(11):31. doi:10.3390/ijerph17113899.
Exclusion reason: Only one time point collected.

299. Levin BJ. *Depression, anxiety, and coping in surfers*. The Wright Institute; 2009.
Exclusion reason: Only one time point collected.

300. Leyland LA, Spencer B, Beale N, Jones T, van Reekum CM. The effect of cycling on cognitive function and well-being in older adults. *PLoS One*. 2019;14(2):e0211779. doi:10.1371/journal.pone.0211779.
Exclusion reason: Intervention did not occur outdoors or intervention does not meet study's definition of nature.

301. Li D, Zhai Y, Xiao Y, Newman G, Wang. Subtypes of Park Use and Self-Reported Psychological Benefits Among Older Adults: A Multilevel Latent Class Analysis Approach. *Landscape and Urban Planning*. 2019;190doi:10.1016/j.landurbplan.2019.103605.
Exclusion reason: Quantitative data not in article, not provided by author.

302. Li LF. *Research on the Effects of Interpersonal Group Counseling for the Depressive University Students*. Huazhong Normal University; 2005.
Exclusion reason: No Translation Available.

303. Li Q. 10-1 Effect of forest environments on human health: An introduction to Forest Medicine. *Journal of the Japanese Society of Balneology, Climatology & Physical Medicine*. 2014;77(5):495-497.
Exclusion reason: Quantitative data not collected (only qualitative data collected) or no original data collected.

304. Li ZH, Huang YB, Chu YH. Forest therapy for schizophrenia. *Chinese journal of physical therapy*. 1998;21(5):305-306.
Exclusion reason: No Translation Available.

305. Liao ML, Ou SJ, Heng Hsieh C, Li Z, Ko CC. Effects of garden visits on people with dementia: A pilot study. *Dementia*. 2020;19(4):1009-1028. doi:10.1177/1471301218793319.
Exclusion reason: Intervention did not occur outdoors or intervention does not meet study's definition of nature.

306. Liden E, Alstersjo K, Gurne FL, Fransson S, Bergbom I. Combining garden therapy and supported employment - a method for preparing women on long-term sick leave for working life. *Scand J Caring Sci*. 2016;30(2):411-8. doi:10.1111/scs.12263.
Exclusion reason: Intervention did not occur outdoors.

307. Lin K. Healthy garden and healthy mind. *Annals of Global Health*. 2016;82(3):533-534.
Exclusion reason: Intervention did not occur outdoors or intervention does not meet study's definition of nature.

308. Lin W, Chen Q, Jiang M, et al. The effect of green space behaviour and per capita area in small urban green spaces on psychophysiological responses. *Landscape and Urban Planning*. 2019;192doi:10.1016/j.landurbplan.2019.103637.

Exclusion reason: No data presented on participants' symptoms of mental illness.

309. Liu YI, Chu FY. Effect of a horticultural therapy program on the emotional well-being of older adults with dementia. *Journal of Research in Education Sciences*. 2018;63(3):257-289. doi:10.6209/JORIES.201809_63(3).0009.
Exclusion reason: Intervention did not occur outdoors or intervention does not meet study's definition of nature.

310. Logan PA, Leighton MP, Walker MF, et al. A multi-centre randomised controlled trial of rehabilitation aimed at improving outdoor mobility for people after stroke: study protocol for a randomised controlled trial. *Trials*. 2012;13:86. doi:10.1186/1745-6215-13-86.
Exclusion reason: quantitative data not collected (only qualitative data collected); no original data collected; or, data reported NOT usable (i.e. no Means/SD).

311. Logue G, Logue D. Euphoria's hiccups gardening our way out of anxiety. *Landscapes*. 2014;16(2):31.
Exclusion reason: Quantitative data not collected (only qualitative data collected) or no original data collected.

312. London AE. *Natural environment associations with mental health and obesity status*. Walden University; 2020.
Exclusion reason: Only one time point collected.

313. Lord E, Coffey M. Identifying and resisting the technological drift: green space, blue space and ecotherapy. *Social Theory and Health*. 2019;doi:10.1057/s41285-019-00099-9.
Exclusion reason: Quantitative data not collected (only qualitative data collected) or no original data collected.

314. Lowden A, Kecklund G, Åkerstedt T, Magnusson Hanson L, Westerlund H. Longitudinal daylight exposure reduces moderate depressive symptoms. *J Sleep Res*. 2016;25:296-297. doi:10.1111/jsr.12446.
Exclusion reason: No data collected on symptoms of mental illness.

315. Lücke C, Braumandl S, Becker B, Moeller S, Custal C, Philipsen A, Müller HHO. Effects of nature-based mindfulness training on resilience/symptom load in professionals with high work-related stress-levels: Findings from the WIN-Study. *Mental Illness*. 2019;11(2):20-24. doi:10.1108/MIJ-10-2019-0001.
Exclusion reason: Intervention did not occur outdoors or intervention does not meet study's definition of nature.

316. Luk KY, Lai KY, Li CC, et al. The effect of horticultural activities on agitation in nursing home residents with dementia. *Int J Geriatr Psychiatry*. 2011;26(4):435-6. doi:10.1002/gps.2493.
Exclusion reason: Intervention did not occur outdoors or intervention does not meet study's definition of nature.

317. Lyu B, Zeng C, Deng S, et al. Bamboo forest therapy contributes to the regulation of psychological responses. *Journal of Forest Research*. 2019;24(1):61-70. doi:10.1080/13416979.2018.1538492.
Exclusion reason: No data presented on participants' symptoms of mental illness.

318. Lyu B, Zeng C, Xie S, et al. Benefits of A Three-Day Bamboo Forest Therapy Session on the Psychophysiology and Immune System Responses of Male College Students. *Int J Environ Res Public Health*. 2019;16(24):08. doi:10.3390/ijerph16244991.

Exclusion reason: quantitative data not collected (only qualitative data collected); no original data collected; or, data reported NOT usable (i.e. no Means/SD).

319. Ma B, Zhou T, Lei S, Wen Y, Htun TT. Effects of urban green spaces on residents' well-being. *Environment, Development and Sustainability*. 2019;21(6):2793-2809. doi:10.1007/s10668-018-0161-8.

Exclusion reason: No data collected that clearly defined nature dosage.

320. Mantler A, Logan AC. Natural environments and mental health. *Advances in Integrative Medicine*. 2015;2(1):5-12. doi:10.1016/j.aimed.2015.03.002.

Exclusion reason: Quantitative data not collected (only qualitative data collected) or no original data collected.

321. Mapes N. Have you been down to the woods today? *Working with Older People: Community Care Policy & Practice*. 2012;16(1):7-16. doi:10.1108/13663661211215105.

Exclusion reason: Quantitative data not collected (only qualitative data collected) or no original data collected.

322. Marselle M. *Growing Resilience through Interaction with Nature (GRIN)*. De Montfort University; 2013.

Exclusion reason: Intervention did not occur outdoors or intervention does not meet study's definition of nature.

323. Marselle M, Warber S, Irvine K. Natural health service: Enhancing wellbeing with group walks in green spaces. *BMC Complement Altern Med*. 2012;12.

Exclusion reason: Intervention did not occur outdoors.

324. Marselle MR, Irvine KN, Warber SL. Walking for well-being: are group walks in certain types of natural environments better for well-being than group walks in urban environments? *Int J Environ Res Public Health*. 2013;10(11):5603-28. doi:10.3390/ijerph10115603.

Exclusion reason: No data collected that clearly defined nature dosage.

325. Marselle MR, Irvine KN, Warber SL. Examining group walks in nature and multiple aspects of well-being: A large-scale study. *Ecopsychology*. 2014;6(3):134-147. doi:10.1089/eco.2014.0027.

Exclusion reason: only one time point collected.

326. Marselle MR, Warber SL, Irvine KN. Growing Resilience through Interaction with Nature: Can Group Walks in Nature Buffer the Effects of Stressful Life Events on Mental Health? *Int J Environ Res Public Health*. 2019;16(6):19. doi:10.3390/ijerph16060986.

Exclusion reason: No data collected that clearly defined nature dosage.

327. Marsh MM. *Present moment enhancement of nursing home residents at Brethren Care Village, Ashland, Ohio with utilization of a garden*. United Theological Seminary; 2006.

Exclusion reason: Intervention did not occur outdoors or intervention does not meet study's definition of nature.

328. Martin L, White MP, Hunt A, Richardson M, Pahl S, Burt J. Nature contact, nature connectedness and associations with health, wellbeing and pro-environmental behaviours. *J Environ Psychol*. 2020;68doi:10.1016/j.jenvp.2020.101389.

Exclusion reason: No data collected on symptoms of mental illness.

329. Martyn P, Brymer E. The relationship between nature relatedness and anxiety. *J Health Psychol*. 2016;21(7):1436-45. doi:10.1177/1359105314555169.

Exclusion reason: only one time point collected.

330. Mather JA, Nemecek D, Oliver K. The effect of a walled garden on behavior of individuals with Alzheimer's. *American Journal of Alzheimer's Disease*. 1997;12(6):252-257. doi:10.1177/153331759701200603.

Exclusion reason: No data collected that clearly defined nature dosage.

331. Mathis JE. *Comparing Treatment Outcomes of Young Adult Males and Females in Wilderness Therapy Programs through a Gender Schema Lens*. Northcentral University; 2018.

Exclusion reason: quantitative data not collected (only qualitative data collected); no original data collected; or, data reported NOT usable (i.e. no Means/SD).

332. Matsouka O, Bebetsos E, Trigonis I, Simakis S. The effects of an outdoor exercise program on mood states among the elderly. *World Leisure Journal*. 2010;52(1):34-40.

Exclusion reason: No data collected on symptoms of mental illness.

333. Matsunaga K, Park B, Ohno N, et al. Effects of rooftop forest-like field on elderly people requiring care -- using sensory evaluation. *Journal of the Japanese Society of Balneology, Climatology & Physical Medicine*. 2009;72(4):256-264.

Exclusion reason: Intervention did not occur outdoors or intervention does not meet study's definition of nature.

334. Matsunaga K, Park B-J, Miyazaki Y. Determination of subjective relaxation effects of a hospital rooftop forest on healthcare workers -- using POMS and STAI-formJYZ. *Journal of the Japanese Society of Balneology, Climatology & Physical Medicine*. 2011;74(3):186-199.

Exclusion reason: Not within time-frame.

335. McAuley E, Mihalko SL. Acute Exercise and Anxiety Reduction: Does the Environment Matter? *J Sport Exerc Psychol*. 1996;18(4):408-419.

Exclusion reason: No data collected that clearly defined nature dosage.

336. McAvoy L, Smith JG, Rynders JE. Outdoor Adventure Programming for Individuals With Cognitive Disabilities Who Present Serious Accommodation Challenges. *Ther Recreation J*. 2006;40(3):182-199.

Exclusion reason: quantitative data not collected (only qualitative data collected); no original data collected; or, data reported NOT usable (i.e. no Means/SD).

337. McClellan J. Benefits of a gardening project for people with dementia in nursing homes. *Nurs Times*. 2018;114(2):8-8.

Exclusion reason: Quantitative data not collected (only qualitative data collected) or no original data collected.

338. McEwan K, Richardson M, Sheffield D, Ferguson FJ, Brindley P. A Smartphone App for Improving Mental Health through Connecting with Urban Nature. *Int J Environ Res Public Health*. 2019;16(18):12. doi:10.3390/ijerph16183373.

Exclusion reason: No data collected that clearly defined nature dosage.

339. McGough G. People in Care Homes Need Fresh Air Like Everyone Else. *Nurs Stand*. 2012;27(7):32-32.

Exclusion reason: Quantitative data not collected (only qualitative data collected) or no original data collected.

340. McManus M. Time spent outdoors is shown to be therapeutic for patients. *Nurs Stand*. 2009;24(4):33-33. doi:10.7748/ns.24.4.33.s45.

Exclusion reason: Quantitative data not collected (only qualitative data collected) or no original data collected.

341. McQuarrie C. WITH NATURE IN MIND: THE ECOTHERAPY MANUAL FOR MENTAL HEALTH PROFESSIONALS. *Ment Health Nurs*. 2017;37(5):22-22.

Exclusion reason: Quantitative data not collected (only qualitative data collected) or no original data collected.

342. Mejia R. Green exercise may be good for your head. *Environmental Science and Technology*. 2010;44(10):3649. doi:10.1021/es101129n.

Exclusion reason: Quantitative data not collected (only qualitative data collected) or no original data collected.

343. Mendes A, Palmer SJ. Therapeutic use of gardening and horticulture in dementia care. *Nursing & Residential Care*. 2016;18(10):546-548. doi:10.12968/nrec.2016.18.10.546.

Exclusion reason: Quantitative data not collected (only qualitative data collected) or no original data collected.

344. Merayo Rodríguez J, Serrano Fuentes N, Marqués Sánchez P. Influencia de los espacios verdes urbanos en la salud mental. *Metas de Enfermería*. 2016;19(9):20-26.

Exclusion reason: quantitative data not collected (only qualitative data collected); no original data collected; or, data reported NOT usable (i.e. no Means/SD).

345. Midden KS, Barnicle T. Evaluating the effects of a horticulture program on the psychological well-being of older persons in a long-term care facility. *Acta Horticulturae*. 2004;639:167-170. doi:10.17660/ActaHortic.2004.639.20.

Exclusion reason: Intervention did not occur outdoors or intervention does not meet study's definition of nature.

346. Mikuniya M, Nakamura K, Okudera K, et al. Time spent outdoors in patients undergoing long-term oxygen therapy and the correlation between dementiaand depressive state. *Hirosaki Medical Journal*. 2011;62(2-4):129-137.

Exclusion reason: No data collected that clearly defined nature dosage.

347. Miller MM. *Impact of Moderate Intensity Physical Activity on Symptom Severity of PTSD among Military Veterans*. Oklahoma State University; 2018.

Exclusion reason: Intervention did not occur outdoors or intervention does not meet study's definition of nature.

348. Miller TA, McCool SF. Coping with stress in outdoor recreational settings: An application of transactional stress theory. *Leisure Sciences*. 2003;25(2-3):257-275. doi:10.1080/01490400306562.
Exclusion reason: No data collected that clearly defined nature dosage.

349. Millet P. Integrating horticulture into the vocational rehabilitation process of individuals with exhaustion syndrome (burnout): A pilot study. *The International Journal of Disability Management Research*. 2009;3(2):39-53. doi:10.1375/jdmr.3.2.39.
Exclusion reason: Intervention did not occur outdoors or intervention does not meet study's definition of nature.

350. Mitchell K, Van Puymbroeck M. Recreational Therapy for Dementia-Related Symptoms in a Long-Term Care Setting: A Case Study. *Ther Recreation J*. 2019;53(2):165-174. doi:10.18666/TRJ-2019-V53-I2-8907.
Exclusion reason: Intervention did not occur outdoors or intervention does not meet study's definition of nature.

351. Mitchell R. Is physical activity in natural environments better for mental health than physical activity in other environments? *Soc Sci Med*. 2013;91:130-4. doi:10.1016/j.socscimed.2012.04.012.
Exclusion reason: Only one time point collected.

352. Miyeon J, Jonides J, Northouse L, Berman MG, Koelling TM, Pressler SJ. Randomized Crossover Study of the Natural Restorative Environment Intervention to Improve Attention and Mood in Heart Failure. *J Cardiovasc Nurs*. 2017;32(5):464-479. doi:10.1097/JCN.0000000000000368.
Exclusion reason: Intervention did not occur outdoors or intervention does not meet study's definition of nature.

353. Mokhtar D, Abdul Aziz NA, Mariapan M. Physiological and psychological health benefits of urban green space in Kuala Lumpur: A comparison between Taman Botani Perdana and Jalan Bukit Bintang. *Pertanika Journal of Social Sciences and Humanities*. 2018;26(3):2101-2114.
Exclusion reason: Quantitative data not in article, not provided by author.

354. Morilla Portela P, Rebollo Rico S, Baena Extremera A, Miranda León MT, Martínez Marín MA. Análisis del perfil sociodemográfico, deportivo y psicológico en una práctica de escalada deportiva de estudiantes universitarios. / Analysis of sociodemographic, sport and psychological profile in a rock-climbing experience on university students. *Retos: Nuevas Perspectivas de Educación Física, Deporte y Recreación*. 2013;(24):9-15.
Exclusion reason: quantitative data not collected (only qualitative data collected); no original data collected; or, data reported NOT usable (i.e. no Means/SD).

355. Moxham L, Liersch-Sumskis S, Taylor E, Patterson C, Brighton R. Preliminary Outcomes of a Pilot Therapeutic Recreation Camp for People with a Mental Illness: Links to Recovery. *Ther Recreation J*. 2015;49(1):61-75.
Exclusion reason: No data collected that clearly defined nature dosage.

356. Muller-Riemenschneider F, Petrunoff N, Sia A, et al. Prescribing Physical Activity in Parks to Improve Health and Wellbeing: Protocol of the Park Prescription Randomized Controlled Trial. *Int J Environ Res Public Health*. 2018;15(6):01. doi:10.3390/ijerph15061154.
Exclusion reason: Correction, need full article.

357. Murphy PF, Miyazaki Y, Detweiler MB, Kim KY. Longitudinal analysis of differential effects on agitation of a therapeutic wander garden for dementia patients based on ambulation ability. *Dementia: The International Journal of Social Research and Practice*. 2010;9(3):355-373. doi:10.1177/1471301210375336.
Exclusion reason: Intervention did not occur outdoors or intervention does not meet study's definition of nature.

358. Nagaya M, Endo H, Kachi T, Abe Y, Ota T. Recreational Rehabilitation Improved Cognitive Function in Vascular Dementia. *J Am Geriatr Soc*. 2005;53(5):911-912. doi:10.1111/j.1532-5415.2005.53278_3.x.
Exclusion reason: Intervention did not occur outdoors or intervention does not meet study's definition of nature.

359. Najjar AH, Foroozandeh E, Gharneh HAA. Horticulture therapy effects on memory and psychological symptoms of depressed male outpatients. *Iranian Rehabilitation Journal*. 2018;16(2):147-154. doi:10.32598/irj.16.2.147.
Exclusion reason: Intervention did not occur outdoors or intervention does not meet study's definition of nature.

360. Nakau M, Imanishi J, Imanishi J, et al. Spiritual care of cancer patients by integrated medicine in urban green space: a pilot study. *Explore: The Journal of Science & Healing*. 2013;9(2):87-90. doi:10.1016/j.explore.2012.12.002.
Exclusion reason: No data collected that clearly defined nature dosage.

361. Negley SK. Recreation therapy as an outpatient intervention. *Ther Recreation J*. 1994;28(1):35-40.
Exclusion reason: Quantitative data not collected (only qualitative data collected) or no original data collected.

362. Neukam S, Kerr J, Crist K, Wasilenko K. The relationship between time spent outdoors and health in older adults. *J Am Geriatr Soc*. 2012;60:S126. doi:10.1111/j.1532-5415.2012.04000.x.
Exclusion reason: No data presented on participants' symptoms of mental illness.

363. Ng KST, Sia A, Ng MKW, et al. Effects of Horticultural Therapy on Asian Older Adults: A Randomized Controlled Trial. *Int J Environ Res Public Health*. 2018;15(8):09. doi:10.3390/ijerph15081705.
Exclusion reason: Intervention did not occur outdoors.

364. O'Connor PJ, Carda RD, Graf BK. Anxiety and intense running exercise in the presence and absence of interpersonal competition. *Int J Sports Med*. 1991;12(4):423-6.
Exclusion reason: No data collected that clearly defined nature dosage.

365. Ochiai H, Ikei H, Song C, et al. Physiological and Psychological Effects of a Forest Therapy Program on Middle-Aged Females. *Int J Environ Res Public Health*. 2015;12(12):15222-32. doi:10.3390/ijerph121214984.
Exclusion reason: No data presented on participants' symptoms of mental illness.

366. Ogryzlo TW. *The outcomes of a wilderness experience: Perception and trip intensity on stress reduction*. Laurentian University; 1998.
Exclusion reason: No data collected on symptoms of mental illness.

367. Osborne M. Improving military personnel resiliency through recreational trail rides. *J Equine Vet Sci*. 2019;76:121. doi:10.1016/j.jevs.2019.03.189.
Exclusion reason: Quantitative data not in article, not provided by author.

368. Osborne TL. OCD Camp: Combining Exposure and Response Prevention and Adventure Therapy for Youth and Young Adults With OCD. *J Am Acad Child Adolesc Psychiatry*. 2018;57(10):S15. doi:10.1016/j.jaac.2018.07.061.
Exclusion reason: quantitative data not collected (only qualitative data collected); no original data collected; or, data reported NOT usable (i.e. no Means/SD).

369. Ouellet-Plamondon C, Girard C, Abdel-Baki A. Adventure therapy for early psychosis: 3-year results. *Early intervention in psychiatry*. 2018;12:202. doi:10.1111/eip.12724.
Exclusion reason: No data collected on symptoms of mental illness.

370. Ouellet-Plamondon C, Nguyen A, Leblanc JP, Abdel-Baki A. Therapeutic adventure: A new tool in the recovery from first episode psychosis. Preliminary findings of a pilot study. *Early intervention in psychiatry*. 2014;8:115. doi:10.1111/eip.12199.
Exclusion reason: Quantitative data not collected (only qualitative data collected) or no original data collected.

371. Ower C, Kemmler G, Vill T, Martini C, Schmitt A, Sperner-Unterweger B, Hufner K. The effect of physical activity in an alpine environment on quality of life is mediated by resilience in patients with psychosomatic disorders and healthy controls. *Eur Arch Psychiatry Clin Neurosci*. 2019;269(5):543-553. doi:10.1007/s00406-018-0930-2.
Exclusion reason: Only one time point collected.

372. Packer S. Taming Trichotillomania While Weeding the Garden. *Psychiatric Times*. 2017;34(7):1-3.
Exclusion reason: Quantitative data not collected (only qualitative data collected) or no original data collected.

373. Pálsdóttir A, Andersson G, Grahn P, Norrving B, Kyrö Wissler S, Petersson IF, Pessah-Rasmussen H. A randomized controlled trial of nature-based post-stroke fatigue rehabilitation (“the nature stroke study” (NASTRU)): Study design and progress report. *Int J Stroke*. 2015;10:430. doi:10.1111/ijs.12479.
Exclusion reason: quantitative data not collected (only qualitative data collected); no original data collected; or, data reported NOT usable (i.e. no Means/SD).

374. Pálsdóttir AM, Stigmar K, Aström M, Grahn P, Norrving B, Pessah-Rasmussen H. “the nature stroke study” (NASTRU), nature-based rehabilitation of post stroke fatigue - A randomized controlled trial. *European Stroke Journal*. 2016;1(1):353. doi:10.1177/2396987316642909.

Exclusion reason: quantitative data not collected (only qualitative data collected); no original data collected; or, data reported NOT usable (i.e. no Means/SD).

375. Palsdottir AM, Stigmar K, Norrving B, Petersson IF, Astrom M, Pessah-Rasmussen H. The nature stroke study; NASTRU: A randomized controlled trial of nature-based post-stroke fatigue rehabilitation. *J Rehabil Med*. 2020;52(2):jrm00020. doi:10.2340/16501977-2652.
Exclusion reason: quantitative data not collected (only qualitative data collected); no original data collected; or, data reported NOT usable (i.e. no Means/SD).

376. Pálsdóttir AM, Stigmar K, Norrving B, Petersson IF, Åström M, Pessah-Rasmussen H. Nature-based rehabilitation to reduce post-stroke fatigue is not effective: A randomized controlled trial. *J Rehabil Med*. 2020;doi:10.2340/16501977-2652.
Exclusion reason: Intervention did not occur outdoors or intervention does not meet study's definition of nature.

377. Pálsdóttir AM, Wissler SK, Nilsson K, Petersson IF, Grahn P, Shoemaker C, Park SA. Nature-based rehabilitation in peri-Urban areas for people with stress-related Illnesses -A controlled prospective study. *Acta Horticulturae*. 2015;1093:31-35. doi:10.17660/ActaHortic.2015.1093.2.
Exclusion reason: quantitative data not collected (only qualitative data collected); no original data collected; or, data reported NOT usable (i.e. no Means/SD).

378. Park BJ, Tsunetsugu Y, Morikawa T, et al. Physiological and psychological effects of walking in stay-in forest therapy. *Nippon Eiseigaku Zasshi - Japanese Journal of Hygiene*. 2014;69(2):98-103.
Exclusion reason: No translation available.

379. Park SA, Lee AY, Son KC, Lee WL, Kim DS. Gardening intervention for physical and psychological health benefits in elderly women at community centers. *HortTechnology*. 2016;26(4):474-483. doi:10.21273/horttech.26.4.474.
Exclusion reason: Intervention did not occur outdoors or intervention does not meet study's definition of nature.

380. Park SA, Shoemaker C, Haub M. Can older gardeners meet the physical activity recommendation through gardening? *HortTechnology*. 2008;18(4):639-643. doi:10.21273/horttech.18.4.639.
Exclusion reason: Intervention did not occur outdoors or intervention does not meet study's definition of nature.

381. Parker CJ, Gladman JR, Drummond AE, et al. A multicentre randomized controlled trial of leisure therapy and conventional occupational therapy after stroke. TOTAL Study Group. Trial of Occupational Therapy and Leisure. *Clin Rehabil*. 2001;15(1):42-52.
Exclusion reason: Intervention did not occur outdoors or intervention does not meet study's definition of nature.

382. Parker CJ, Gladman JRF, Drummond AER, et al. A multicentre randomized controlled trial of leisure therapy and conventional occupational therapy after stroke. *Clin Rehabil*. 2001;15(1):42-52. doi:10.1191/026921501666968247.
Exclusion reason: Intervention did not occur outdoors or intervention does not meet study's definition of nature.

383. Parkinson S, Lowe C, Vecsey T. The therapeutic benefits of horticulture in a mental health service. *The British Journal of Occupational Therapy*. 2011;74(11):525-534. doi:10.4276/030802211X13204135680901.
Exclusion reason: No data collected on symptoms of mental illness.

384. Passmore T, Lane S. Exercise as treatment for depression: a therapeutic recreation intervention. *American Journal of Recreation Therapy*. 2006;5(3):31-41.
Exclusion reason: Intervention did not occur outdoors or intervention does not meet study's definition of nature.

385. Patten SB, Williams JA, Lavorato DH, Bulloch AGM. Recreational physical activity ameliorates some of the negative impact of major depression on health-related quality of life. *Frontiers in Psychiatry*. 2013;4(APR)doi:10.3389/fpsyt.2013.00022.
Exclusion reason: No data collected that clearly defined nature dosage.

386. Peacock SM, McKenna J, Carless D, Cooke C. Outcomes from a One-Week Adapted Sport and Adapted Adventure Recovery Programme for Military Personnel. *Sports*. 2019;7(6):31. doi:10.3390/sports7060135.
Exclusion reason: Intervention did not occur outdoors or intervention does not meet study's definition of nature.

387. Pearson DG, Craig T. The great outdoors? Exploring the mental health benefits of natural environments. *Front Psychol*. 2014;5:1178. doi:10.3389/fpsyg.2014.01178.
Exclusion reason: Wrong study design.

388. Pedersen I, Martinsen EW, Berget B, Braastad BO. Farm animal-assisted intervention for people with clinical depression: A randomized controlled study. *Anthrozoös*. 2012;25(2):149-160. doi:10.2752/175303712X13316289505260.
Exclusion reason: Intervention did not occur outdoors or intervention does not meet study's definition of nature.

389. Pedersen I, Nordaunet T, Martinsen EW, Berget B, Braastad BO. Farm Animal-Assisted Intervention: Relationship between Work and Contact with Farm Animals and Change in Depression, Anxiety, and Self-Efficacy Among Persons with Clinical Depression. *Issues Ment Health Nurs*. 2011;32(8):493-500. doi:10.3109/01612840.2011.566982.
Exclusion reason: Intervention did not occur outdoors or intervention does not meet study's definition of nature.

390. Pegg S. Improving recreation therapy outcomes for community-based consumers of a regional mental health service. *American Journal of Recreation Therapy*. 2003;2(2):35-45.
Exclusion reason: Intervention did not occur outdoors or intervention does not meet study's definition of nature.

391. Pelham TW, Campagna PD, Ritvo PG, Birnie WA. The effects of exercise therapy on clients in a psychiatric rehabilitation program. *Psychosocial Rehabilitation Journal*. 1993;16(4):75-84. doi:10.1037/h0095650.
Exclusion reason: Intervention did not occur outdoors or intervention does not meet study's definition of nature.

392. Perkins S, Searight HR, Ratwik S. Walking in a natural winter setting to relieve attention fatigue: A pilot study. *Psychology*. 2011;2(8):777-780. doi:10.4236/psych.2011.28119.
Exclusion reason: quantitative data not collected (only qualitative data collected); no original data collected; or, data reported NOT usable (i.e. no Means/SD).

393. Pieters HC, Ayala L, Schneider A, Wicks N, Levine-Dickman A, Clinton S. Gardening on a psychiatric inpatient unit: Cultivating recovery. *Arch Psychiatr Nurs*. 2019;33(1):57-64. doi:10.1016/j.apnu.2018.10.001.
Exclusion reason: Quantitative data not collected (only qualitative data collected) or no original data collected.

394. Pillemer K, Fuller-Rowell TE, Reid MC, Wells NM. Environmental volunteering and health outcomes over a 20-year period. *Gerontologist*. 2010;50(5):594-602. doi:10.1093/geront/gnq007.
Exclusion reason: No data collected that clearly defined nature dosage.

395. Pithouse M. Treating PTSD using the natural environment. *Nurs Times*. 2016;112(5):16-8.
Exclusion reason: quantitative data not collected (only qualitative data collected); no original data collected; or, data reported NOT usable (i.e. no Means/SD).

396. Po-Ju C, Song R, Lin Y. Air Pollution as a Moderator in the Association Between Leisure Activities and Well-Being in Urban China. *Journal of Happiness Studies*. 2019;20(8):2401-2430. doi:10.1007/s10902-018-0055-3.
Exclusion reason: Did not separate participant group with mental illness or symptoms.

397. Pollock A. Going outside is essential for health and well-being. *Journal of Dementia Care*. 2012;20(3):34-37.
Exclusion reason: Quantitative data not collected (only qualitative data collected) or no original data collected.

398. Pope ZC, Brito JN, Mitchell NR, Schneider IE, Horton TH, Pereira MA. Heart rate variability differences between green and suburban walking: A pilot crossover study. *Circulation*. 2019;139doi:10.1161/circ.139.suppl_1.P099.
Exclusion reason: No data presented on participants' symptoms of mental illness.

399. Pranikoff JR. *Words of recovery: Finding meaning in illness and injury*. City University of New York; 2010.
Exclusion reason: Intervention did not occur outdoors or intervention does not meet study's definition of nature.

400. Quick JS, Robishaw SB, Baylor KA, Snyder DI, Han JJ. Vegetable Garden as Therapeutic Horticulture for Patients with Chronic Pain. *Pain Med*. 2017;18(11):2252-2254. doi:10.1093/pm/pnx065.
Exclusion reason: Quantitative data not collected (only qualitative data collected) or no original data collected.

401. Rabinowitz E. Recreation interventions for individuals with attention deficit hyperactivity disorder. *American Journal of Recreation Therapy*. 2004;3(3):31-35.

Exclusion reason: Quantitative data not collected (only qualitative data collected) or no original data collected.

402. Rader SS. *Ecopsychology revealed: An empirical look at the benefits of nature experience for human beings and the world*. Alliant International University, Los Angeles; 2009.
Exclusion reason: No data presented on participants' symptoms of mental illness.

403. Rantakokko M, Pakkala I, Ayravainen I, Rantanen T. The effect of out-of-home activity intervention delivered by volunteers on depressive symptoms among older people with severe mobility limitations: a randomized controlled trial. *Aging Ment Health*. 2015;19(3):231-8. doi:10.1080/13607863.2014.924092.
Exclusion reason: Intervention did not occur outdoors or intervention does not meet study's definition of nature.

404. Rappe E, Kivelä SL. Effects of garden visits on long-term care residents as related to depression. *HortTechnology*. 2005;15(2):298-303.
Exclusion reason: Intervention did not occur outdoors or intervention does not meet study's definition of nature.

405. Rappe E, Koivunen T, Korpela E. Group gardening in mental outpatient care. *Therapeutic Communities*. 2008;29(3):273-284.
Exclusion reason: quantitative data not collected (only qualitative data collected); no original data collected; or, data reported NOT usable (i.e. no Means/SD).

406. Rappe E, Topo P. Contact with outdoor greenery can support competence among people with dementia. *Journal of Housing for the Elderly*. 2007;21(3/4):229-248. doi:10.1300/j081v21n03_12.
Exclusion reason: quantitative data not collected (only qualitative data collected); no original data collected; or, data reported NOT usable (i.e. no Means/SD).

407. Regan CL, Horn SA. To nature or not to nature: Associations between environmental preferences, mood states and demographic factors. *J Environ Psychol*. 2005;25(1):57-66. doi:10.1016/j.jenvp.2005.01.001.
Exclusion reason: Only one time point collected.

408. Reklaitiene R, Grazuleviciene R, Dedele A, et al. The relationship of green space, depressive symptoms and perceived general health in urban population. *Scandinavian Journal of Public Health*. 2014;42(7):669-76. doi:10.1177/1403494814544494.
Exclusion reason: Wrong study design.

409. Rendell M, Carroll D. 'Why don't we go into the garden?'. *Journal of Dementia Care*. 2015;23(2):16-18.
Exclusion reason: Quantitative data not collected (only qualitative data collected) or no original data collected.

410. Repke MA, Berry MS, Conway LG, 3rd, Metcalf A, Hensen RM, Phelan C. How does nature exposure make people healthier?: Evidence for the role of impulsivity and expanded space perception. *PLoS One*. 2018;13(8):e0202246. doi:10.1371/journal.pone.0202246.
Exclusion reason: No data collected that clearly defined nature dosage.

411. Richards HJ, Kafami DM. Impact of Horticultural Therapy on Vulnerability and Resistance to Substance Abuse among Incarcerated Offenders. *Journal of Offender Rehabilitation*. 1999;29(3-4):183-193.

Exclusion reason: No data collected that clearly defined nature dosage.

412. Richards KE. *A feminist analysis of developing an adventure therapy intervention for the treatment of eating disorders in women*. Liverpool John Moores University; 2008.

Exclusion reason: No data collected that clearly defined nature dosage.

413. Richardson M, McEwan K, Garip G. 30 days wild: Who benefits most? *Journal of Public Mental Health*. 2018;17(3):95-104. doi:10.1108/JPMH-02-2018-0018.

Exclusion reason: No data collected on symptoms of mental illness.

414. Rogers CM, Mallinson T, Peppers D. High-intensity sports for posttraumatic stress disorder and depression: feasibility study of ocean therapy with veterans of Operation Enduring Freedom and Operation Iraqi Freedom. *Am J Occup Ther*. 2014;68(4):395-404. doi:10.5014/ajot.2014.011221.

Exclusion reason: Quantitative data not in article, not provided by author.

415. Rogerson M. *Green exercise: combined influence of environment and exercise to promote wellbeing*. University of Essex; 2016.

Exclusion reason: Quantitative data not in article, not provided by author.

416. Rogerson M, Brown DK, Sandercock G, Wooller JJ, Barton J. A comparison of four typical green exercise environments and prediction of psychological health outcomes. *Perspectives in Public Health*. 2016;136(3):171-80. doi:10.1177/1757913915589845.

Exclusion reason: Did not separate participant group with mental illness or symptoms.

417. Roijen Pv. A bird's-eye view of the back garden. *Nursing & Residential Care*. 2019;21(1):5-5. doi:10.12968/nrec.2019.21.1.5.

Exclusion reason: Quantitative data not collected (only qualitative data collected) or no original data collected.

418. Rones N. into the WOODS. *Yoga Journal*. 2015;(276):25-27.

Exclusion reason: Quantitative data not collected (only qualitative data collected) or no original data collected.

419. Rosenberg RS, Lange W, Zebrack B, Moulton S, Kosslyn SM. An outdoor adventure program for young adults with cancer: positive effects on body image and psychosocial functioning. *J Psychosoc Oncol*. 2014;32(5):622-36. doi:10.1080/07347332.2014.936652.

Exclusion reason: Quantitative data not in article, not provided by author.

420. Rowden-Racette K. Introducing the Great Outdoors. *ASHA Leader*. 2014;19(1):20-21.

Exclusion reason: Quantitative data not collected (only qualitative data collected) or no original data collected.

421. Russell KC, Gillis HLL, Kivlighan DM. Process factors explaining psycho-social outcomes in adventure therapy. *Psychotherapy: Theory, Research, Practice, Training*. 2017;54(3):273-280. doi:10.1037/pst0000131.
Exclusion reason: No data collected that clearly defined nature dosage.

422. Russoniello C. The effectiveness of prescribed recreational activity in decreasing biochemical stress and improving mood in alcoholic patients. *American Journal of Recreation Therapy*. 2008;7(3):23-33.
Exclusion reason: Intervention did not occur outdoors or intervention does not meet study's definition of nature.

423. Ryu J, Jung JH, Kim J, et al. Outdoor cycling improves clinical symptoms, cognition and objectively measured physical activity in patients with schizophrenia: A randomized controlled trial. *J Psychiatr Res*. 2020;120:144-153. doi:10.1016/j.jpsychires.2019.10.015.
Exclusion reason: Intervention did not occur outdoors or intervention does not meet study's definition of nature.

424. Sachs JJ. *The impact of a modified wilderness camping program on the social interactions and social expectations of behavior disordered adolescents*. Southern Illinois University at Carbondale; 1982.
Exclusion reason: Not within time-frame.

425. Sahlin E, Ahlborg G, Jr., Tenenbaum A, Grahn P. Using nature-based rehabilitation to restart a stalled process of rehabilitation in individuals with stress-related mental illness. *Int J Environ Res Public Health*. 2015;12(2):1928-51. doi:10.3390/ijerph120201928.
Exclusion reason: No data collected that clearly defined nature dosage.

426. Sahlin E, Ahlborg G, Tenenbaum A, Grahn P. Correction: Sahlin, E., et al. Using Nature-Based Rehabilitation to Restart a Stalled Process of Rehabilitation in Individuals with Stress-Related Mental Illness. Int. J. Environ. Res. Public Health 2015, 12, 1928-1951. *Int J Environ Res Public Health*. 2015;12(6):6946-7. doi:10.3390/ijerph120606946.
Exclusion reason: quantitative data not collected (only qualitative data collected); no original data collected; or, data reported NOT usable (i.e. no Means/SD).

427. Sahlin E, Johansson B, Karlsson PO, Loberg J, Niklasson M, Grahn P. Improved Wellbeing for Both Caretakers and Users from A Zoo-Related Nature Based Intervention-A Study at Nordens Ark Zoo, Sweden. *Int J Environ Res Public Health*. 2019;16(24):05. doi:10.3390/ijerph16244929.
Exclusion reason: No data collected on symptoms of mental illness.

428. Salvi GP, Quarenghi A, Manzoni L, et al. Ecological test of San Pellegrino Terme. *Cerebrovasc Dis*. 2014;37:307.
Exclusion reason: No data collected that clearly defined nature dosage.

429. Sarno MT, Chambers N. A horticultural therapy program for individuals with acquired aphasia. *Act Adapt Aging*. 1997;22(1-2):81-91. doi:10.1300/J016v22n01_07.
Exclusion reason: Wrong study design.

430. Scheidhacker M, Bender W, Vaitl P. Die Wirksamkeit des therapeutischen Reitens bei der Behandlung chronisch schizophrener Patienten: Experimentelle Ergebnisse und klinische Erfahrungen =

The efficacy of horse-riding in the treatment of chronic schizophrenic patients: Experimental results and clinical experiences. *Der Nervenarzt*. 1991;62(5):283-287.
Exclusion reason: No Translation Available.

431. Scheinfeld DE, Rochlen AB, Russell ML. The impact of outward bound programming on psychosocial functioning for male military veterans. *Psychol Men Masc*. 2017;18(4):400-408. doi:10.1037/men0000066.
Exclusion reason: Did not separate participant group with mental illness or symptoms.

432. Schmalzel D, Chapleau A, Wagenfeld A, Chase C. Measuring Mental Health Outcomes of a Pilot Therapeutic Gardening Program. *SIS Quarterly Practice Connections*. 2019;4(2):18-20.
Exclusion reason: Quantitative data not collected (only qualitative data collected) or no original data collected.

433. Schols JM, van der Schriek-van Meel C. Day care for demented elderly in a dairy farm setting: positive first impressions. *J Am Med Dir Assoc*. 2006;7(7):456-9.
Exclusion reason: No data collected that clearly defined nature dosage.

434. Schumacher JM, Navar N. Promoting physical health and psychosocial well-being through the Sociable Tandem Cycle Recreation Program with adults with mood disorder. *Annual in Therapeutic Recreation*. 2010;18:114-130.
Exclusion reason: Intervention did not occur outdoors or intervention does not meet study's definition of nature.

435. Schuz B, Czerniawski A, Davie N, et al. Leisure Time Activities and Mental Health in Informal Dementia Caregivers. *Applied Psychology Health and Well-being*. 2015;7(2):230-48. doi:10.1111/aphw.12046.
Exclusion reason: Intervention did not occur outdoors.

436. Schwarzmuller-Erber G, Stummer H, Maier M, Kundi M. Nature Relatedness of Recreational Horseback Riders and Its Association with Mood and Wellbeing. *Int J Environ Res Public Health*. 2020;17(11):10. doi:10.3390/ijerph17114136.
Exclusion reason: No data collected that clearly defined nature dosage.

437. Scorcu M, Palazzolo G, Ibba G, et al. From sardinia, Italy, to the top of the world: Trekking as therapy. Modification of physiological parameters and perception of health status in a group of patients with severe psychiatric disorder. *Clin J Sport Med*. 2014;24(3):e45-e46. doi:10.1097/JSM.0000000000000099.
Exclusion reason: No data collected that clearly defined nature dosage.

438. Sempik J, Rickhuss C, Beeston A. The effects of social and therapeutic horticulture on aspects of social behaviour. *The British Journal of Occupational Therapy*. 2014;77(6):313-319. doi:10.4276/030802214X14018723138110.
Exclusion reason: Intervention did not occur outdoors or intervention does not meet study's definition of nature.

439. Shanahan DF, Bush R, Gaston KJ, Lin BB, Dean J, Barber E, Fuller RA. Health Benefits from Nature Experiences Depend on Dose. *Sci Rep*. 2016;6:28551. doi:10.1038/srep28551.

Exclusion reason: Only one time point collected.

440. Shaw PA. Access to Nature Improves Well-Being. *Caring for the Ages*. 2016;17(5):12-12. doi:10.1016/j.carage.2016.04.014.
Exclusion reason: Quantitative data not collected (only qualitative data collected) or no original data collected.

441. Shellman A, Hill E. Flourishing through Resilience: The Impact of a College Outdoor Education Program. *Journal of Park & Recreation Administration*. 2017;35(4):59-68.
Exclusion reason: No data presented on participants' symptoms of mental illness.

442. Shin WS, Shin CS, Yeoun PS. The influence of forest therapy camp on depression in alcoholics. *Environ Health Prev Med*. 2012;17(1):73-6. doi:10.1007/s12199-011-0215-0.
Exclusion reason: Quantitative data not in article, not provided by author.

443. Singley K, Griner C, DeDominic T, Wagenfeld A. Therapy in the Natural Environment. *OT Practice*. 2016;21(11):21-24.
Exclusion reason: Quantitative data not collected (only qualitative data collected) or no original data collected.

444. Siu AMH, Kam M, Mok I. Horticultural Therapy Program for People with Mental Illness: A Mixed-Method Evaluation. *Int J Environ Res Public Health*. 2020;17(3):22. doi:10.3390/ijerph17030711.
Exclusion reason: Intervention did not occur outdoors.

445. Smethurst T. *Well-Being and Ethnic Identity Promotion for Aboriginal Youth: A Community Based Mixed Methods Study of Tribal Journeys*. University of Victoria; 2013.
Exclusion reason: No data collected on symptoms of mental illness.

446. Smidl S, Mitchell DM, Creighton CL. Outcomes of a Therapeutic Gardening Program in a Mental Health Recovery Center. *Occupational Therapy in Mental Health*. 2017;33(4):374-385. doi:10.1080/0164212X.2017.1314207.
Exclusion reason: No data collected that clearly defined nature dosage.

447. Smith DJ, McCallion P. Alleviating Stress for Family Caregivers of Frail Elders Using Horticultural Therapy. *Activities, Adaptation and Aging*. 1997;22(1-2):93-105.
Exclusion reason: Quantitative data not collected (only qualitative data collected) or no original data collected.

448. Smith NT. *Effectiveness of a guided wilderness experience on improved client self-esteem, self-efficacy, anxiety, and depression*. The University of Utah; 2010.
Exclusion reason: No data collected that clearly defined nature dosage.

449. Son KC, Um SJ, Kim SY, Song JE, Kwack HR. Effect of horticultural therapy on the changes of self-esteem and sociality of individuals with chronic schizophrenia. *Acta Horticulturae*. 2004;639:185-191. doi:10.17660/ActaHortic.2004.639.23.
Exclusion reason: No data collected that clearly defined nature dosage.

450. Song C, Ikei H, Igarashi M, Miwa M, Takagaki M, Miyazaki Y. Physiological and psychological responses of young males during spring-time walks in urban parks. *J Physiol Anthropol*. 2014;33:8. doi:10.1186/1880-6805-33-8.

Exclusion reason: Quantitative data not in article, not provided by author.

451. Song C, Ikei H, Igarashi M, Takagaki M, Miyazaki Y. Physiological and Psychological Effects of a Walk in Urban Parks in Fall. *Int J Environ Res Public Health*. 2015;12(11):14216-28. doi:10.3390/ijerph121114216.

Exclusion reason: Quantitative data not in article, not provided by author.

452. Song C, Ikei H, Kagawa T, Miyazaki Y. Effects of Walking in a Forest on Young Women. *Int J Environ Res Public Health*. 2019;16(2):15. doi:10.3390/ijerph16020229.

Exclusion reason: No data presented on participants' symptoms of mental illness.

453. Song C, Ikei H, Kobayashi M, et al. Effect of forest walking on autonomic nervous system activity in middle-aged hypertensive individuals: a pilot study. *Int J Environ Res Public Health*. 2015;12(3):2687-99. doi:10.3390/ijerph120302687.

Exclusion reason: Quantitative data not in article, not provided by author.

454. Song C, Ikei H, Park BJ, Lee J, Kagawa T, Miyazaki Y. Psychological Benefits of Walking through Forest Areas. *Int J Environ Res Public Health*. 2018;15(12):10. doi:10.3390/ijerph15122804.

Exclusion reason: Quantitative data not in article, not provided by author.

455. Song C, Ikei H, Park BJ, Lee J, Kagawa T, Miyazaki Y. Correction: Song, C.; Ikei, H.; Park, B.J.; Lee, J.; Kagawa, T.; Miyazaki, Y. Psychological benefits of walking through forest areas (int. j. environ. res. public health (2018), 15, 2804, 10.3390/ijerph15122804). *Int J Environ Res Public Health*. 2020;17(4)doi:10.3390/ijerph17041316.

Exclusion reason: quantitative data not collected (only qualitative data collected); no original data collected; or, data reported NOT usable (i.e. no Means/SD).

456. Song C, Joung D, Ikei H, et al. Physiological and psychological effects of walking on young males in urban parks in winter. *J Physiol Anthropol*. 2013;32:18. doi:10.1186/1880-6805-32-18.

Exclusion reason: Did not separate participant group with mental illness or symptoms.

457. Spencer-Cavaliere N, Bowtell D, Langager ML. Informing Therapeutic Practice through the Walking Program Experiences of Rehabilitation Clients With Stroke and Traumatic Brain Injury. *Ther Recreation J*. 2014;48(3):247-261.

Exclusion reason: Quantitative data not collected (only qualitative data collected) or no original data collected.

458. Stauber S. Subsistence gillnet salmon fishing: A community normalization experience for persons with long-term mental illness. *Psychosocial Rehabilitation Journal*. 1993;16(4):125-129. doi:10.1037/h0095644.

Exclusion reason: Quantitative data not collected (only qualitative data collected) or no original data collected.

459. Stigsdotter UK, Ekholm O, Schipperijn J, Toftager M, Kamper-Jorgensen F, Randrup TB. Health promoting outdoor environments--associations between green space, and health, health-related quality

of life and stress based on a Danish national representative survey. *Scandinavian Journal of Public Health*. 2010;38(4):411-7. doi:10.1177/1403494810367468.
Exclusion reason: No data collected that clearly defined nature dosage.

460. Sudmann TT. Communitas and Friluftsliv: Equine-facilitated activities for drug users. *Community Development Journal*. 2018;53(3):556-573. doi:10.1093/cdj/bsy026.
Exclusion reason: Quantitative data not collected (only qualitative data collected) or no original data collected.

461. Suemaru S, Maeba Y, Suemaru K, Nishioka T, Takao T, Hashimoto K. Effects of a long term trial of Fukuyama Yuai-Outdoor Activity Care Programme (FY-OACP) on behavioural disorders, hypothalamic-pitutary-adrenal axis and immune activity in demented elderly patients. *Alzheimer's disease: biology, diagnosis and therapeutics*. 1997:805-813.
Exclusion reason: quantitative data not collected (only qualitative data collected); no original data collected; or, data reported NOT usable (i.e. no Means/SD).

462. Suija K, Pechter Ü, Kalda R, Tähepõld H, Maaroos J, Maaroos H. Physical activity of depressed patients and their motivation to exercise: Nordic walking in family practice. *Int J Rehabil Res*. 2009;32(2):132-138. doi:10.1097/MRR.0b013e32831e44ef.
Exclusion reason: Intervention did not occur outdoors.

463. Suzuki T, Murase S. Influence of outdoor activity and indoor activity on cognition decline: use of an infrared sensor to measure activity. *Telemed J E Health*. 2010;16(6):686-90. doi:10.1089/tmj.2009.0175.
Exclusion reason: No data collected that clearly defined nature dosage.

464. Takabayashi K, Iwatsu K, Ichinohe T, et al. Decline in activities of daily living by acute heart failure as a predictor of major adverse cardiac and cerebrovascular event in patients with heart failure in community-based registry. *Eur Heart J*. 2018;39:88. doi:10.1093/eurheartj/ehy564.P647.
Exclusion reason: No data collected that clearly defined nature dosage.

465. Tan Z, Lau KK, Roberts AC, Chao ST, Ng E. Designing Urban Green Spaces for Older Adults in Asian Cities. *Int J Environ Res Public Health*. 2019;16(22):12. doi:10.3390/ijerph16224423.
Exclusion reason: No data collected on symptoms of mental illness.

466. Tanaka M, Yamada H, Nakamura T, Ishii A, Watanabe Y. Fatigue-recovering effect of a house designed with open space. *Explore: The Journal of Science & Healing*. 2013;9(2):82-6. doi:10.1016/j.explore.2012.12.006.
Exclusion reason: Wrong intervention.

467. Tateuchi Y, Shimada R, Urano Y, et al. Psychological effects of occupational therapy with gardening on psychiatric patients. *IRYO - Japanese Journal of National Medical Services*. 2004;58(4):211-215.
Exclusion reason: quantitative data not collected (only qualitative data collected); no original data collected; or, data reported NOT usable (i.e. no Means/SD).

468. Tauber PG. *An exploration of the relationships among connectedness to nature, quality of life, and mental health*. Utah State University; 2012.

Exclusion reason: only one time point collected.

469. Tessneer S. *Psychophysiological Responses to Social Stress in Indoor and Outdoor, Natural Environments*. Indiana University; 2019.
Exclusion reason: No data presented on participants' symptoms of mental illness.

470. Thelander VB, Wahlin TBR, Olofsson L, Heikkilä K, Sonde L. Gardening activities for nursing home residents with dementia. *Adv Physiother*. 2008;10(1):53-56. doi:10.1080/14038190701256469.
Exclusion reason: quantitative data not collected (only qualitative data collected); no original data collected; or, data reported NOT usable (i.e. no Means/SD).

471. Thomas EJ. *Exploring the Benefites of Urban Forestry and Recreation for Individuals Dealing with Low to Moderate Depression and Anxiety*. Southern University and Agricultural and Mechanical College; 2017.
Exclusion reason: Intervention did not occur outdoors or intervention does not meet study's definition of nature.

472. Thomas J, Al-Anouti F. Sun Exposure and Behavioral Activation for Hypovitaminosis D and Depression: A Controlled Pilot Study. *Community Ment Health J*. 2018;54(6):860-865. doi:10.1007/s10597-017-0209-5.
Exclusion reason: Intervention did not occur outdoors or intervention does not meet study's definition of nature.

473. Thomas SP. Therapeutic horticulture deserves wider implementation. *Issues Ment Health Nurs*. 2014;35(3):155. doi:10.3109/01612840.2014.883790.
Exclusion reason: Quantitative data not collected (only qualitative data collected) or no original data collected.

474. Thompson CW, Elizalde A, Cummins S, et al. Enhancing Health Through Access to Nature: How Effective are Interventions in Woodlands in Deprived Urban Communities? A Quasi-experimental Study in Scotland, UK. *Sustainability*. 2019;11(12):3317. doi:10.3390/su11123317.
Exclusion reason: No data collected that clearly defined nature dosage.

475. Thompson JES. *The impact of an 8-week green-exercise programme on systemic health, and on markers associated with cardiovascular disease risk*. Cardiff Metropolitan University; 2014.
Exclusion reason: Quantitative data not in article, not provided by author.

476. Thomson LJ, Morse N, Elsden E, Chatterjee HJ. Art, nature and mental health: assessing the biopsychosocial effects of a 'creative green prescription' museum programme involving horticulture, artmaking and collections. *Perspectives in Public Health*. 2020:1757913920910443. doi:10.1177/1757913920910443.
Exclusion reason: No data collected on symptoms of mental illness.

477. Tockwell-Villandry A. Nature's way. *Mental Health Practice*. 2002;5(7):22-23.
Exclusion reason: Quantitative data not collected (only qualitative data collected) or no original data collected.

478. Torres ER, Sampselle CM, Ronis DL, Neighbors HW, Gretebeck KA. Gardening/Yard Work and Depressive Symptoms in African Americans. *Arch Psychiatr Nurs*. 2016;30(2):155-61. doi:10.1016/j.apnu.2015.08.004.
Exclusion reason: quantitative data not collected (only qualitative data collected); no original data collected; or, data reported NOT usable (i.e. no Means/SD).

479. Tri Suraning W, Muhamad Thohar A, Suhartini I. Increasing Attention and Mood of Post-Stroke Clients using Natural Restorative Environment. *Disability, CBR & Inclusive Development*. 2019;30(4):39-48. doi:10.5463/DCID.v30i4.856.
Exclusion reason: Intervention did not occur outdoors or intervention does not meet study's definition of nature.

480. Triguero-Mas M, Donaire-Gonzalez D, Seto E, et al. Natural outdoor environments and mental health: Stress as a possible mechanism. *Environ Res*. 2017;159:629-638. doi:10.1016/j.envres.2017.08.048.
Exclusion reason: No data collected on symptoms of mental illness.

481. Triguero-Mas M, Gidlow CJ, Martinez D, et al. The effect of randomised exposure to different types of natural outdoor environments compared to exposure to an urban environment on people with indications of psychological distress in Catalonia. *PLoS One*. 2017;12(3):e0172200. doi:10.1371/journal.pone.0172200.
Exclusion reason: Quantitative data not in article, not provided by author.

482. Trudeau RP. Nature: The Ultimate Antidepressant. *Natural Solutions*. 2013;(152):61-62.
Exclusion reason: Quantitative data not collected (only qualitative data collected) or no original data collected.

483. Tseng TA. The Effect of Nature therapy for Stress, Anxiety, Depression and Demoralization on Breast Cancer Patients. *GSTF Journal of Nursing & Health Care*. 2017;5(1):1-11. doi:10.5176/2345-718X_5.1.155.
Exclusion reason: No data presented on participants' symptoms of mental illness.

484. Tyrväinen L, Ojala A, Neuvonen M, Borodulin K, Lanki T. Health and well-being from forests – experience from finnish research. *Sante Publique (Bucur)*. 2019;31:249-256. doi:10.3917/spub.190.0249.
Exclusion reason: quantitative data not collected (only qualitative data collected); no original data collected; or, data reported NOT usable (i.e. no Means/SD).

485. Ura C, Okamura T, Yamazaki S, et al. [The development of care farming for elderly people with cognitive impairment to enhance social inclusion: A feasibility study of rice-farming care for elderly people with cognitive impairment]. *Nippon Ronen Igakkai Zasshi - Japanese Journal of Geriatrics*. 2018;55(1):106-116. doi:10.3143/geriatrics.55.106.
Exclusion reason: Intervention did not occur outdoors or intervention does not meet study's definition of nature.

486. van den Berg M, van Poppel M, Smith G, et al. Does time spent on visits to green space mediate the associations between the level of residential greenness and mental health? *Urban Forestry and Urban Greening*. 2017;25:94-102. doi:10.1016/j.ufug.2017.04.010.
Exclusion reason: only one time point collected.

487. van den Berg M, van Poppel M, van Kamp I, et al. Visiting green space is associated with mental health and vitality: A cross-sectional study in four european cities. *Health & Place*. 2016;38:8-15. doi:10.1016/j.healthplace.2016.01.003.
Exclusion reason: No data collected that clearly defined nature dosage.

488. van den Berg MM, van Poppel M, van Kamp I, et al. Do Physical Activity, Social Cohesion, and Loneliness Mediate the Association Between Time Spent Visiting Green Space and Mental Health? *Environ Behav*. 2019;51(2):144-166. doi:10.1177/0013916517738563.
Exclusion reason: only one time point collected.

489. Vargo BD. Can You Dig It? *Arthritis Today*. 2013;27(3):66-66.
Exclusion reason: Quantitative data not collected (only qualitative data collected) or no original data collected.

490. Vauter A. *Relationship between frequencies and satisfaction with recreation therapy interventions and self-efficacy, loneliness, cooperation and trust among persons who abuse substances*. Temple University; 1998.
Exclusion reason: Intervention did not occur outdoors.

491. Vázquez-Ortiz J, Vázquez-Ramos MJ, Álvarez-Serrano M, Reyes-Alcaide M, Martínez-Domingo R, Nadal-Delgado M, Cruzado JA. Intensive group psychotherapy intervention in a natural environment for the treatment of burnout in an oncology nursing team. *Psicooncologia*. 2012;9(1):161-182. doi:10.5209/rev_PSIC.2012.v9.n1.39145.
Exclusion reason: Intervention did not occur outdoors or intervention does not meet study's definition of nature.

492. Verra ML, Angst F, Beck T, Lehmann S, Brioschi R, Schneiter R, Aeschlimann A. Horticultural therapy for patients with chronic musculoskeletal pain: results of a pilot study. *Altern Ther Health Med*. 2012;18(2):44-50.
Exclusion reason: Intervention did not occur outdoors.

493. Vordal AS. Outdoor activity group. *Nordic Journal of Psychiatry, Supplement*. 1998;52(41):186.
Exclusion reason: quantitative data not collected (only qualitative data collected); no original data collected; or, data reported NOT usable (i.e. no Means/SD).

494. Vujcic M, Tomicevic-Dubljevic J, Grbic M, Lecic-Tosevski D, Vukovic O, Toskovic O. Nature based solution for improving mental health and well-being in urban areas. *Environ Res*. 2017;158:385-392. doi:10.1016/j.envres.2017.06.030.
Exclusion reason: No data collected that clearly defined nature dosage.

495. Wahrborg P, Petersson IF, Grahn P. Nature-assisted rehabilitation for reactions to severe stress and/or depression in a rehabilitation garden: long-term follow-up including comparisons with a matched population-based reference cohort. *J Rehabil Med*. 2014;46(3):271-6. doi:10.2340/16501977-1259.
Exclusion reason: No data collected that clearly defined nature dosage.

496. Wallis V. *A mixed method evaluation of a wilderness therapy programme for "youth at risk"*. University of Essex; 2012.

Exclusion reason: quantitative data not collected (only qualitative data collected); no original data collected; or, data reported NOT usable (i.e. no Means/SD).

497. Walter KH, Otis NP, Glassman LH, Ray TN, Michalewicz-Kragh B, Kobayashi Elliott KT, Thomsen CJ. Comparison of surf and hike therapy for active duty service members with major depressive disorder: Study protocol for a randomized controlled trial of novel interventions in a naturalistic setting. *Contemporary Clinical Trials Communications*. 2019;16:100435. doi:10.1016/j.conctc.2019.100435.
Exclusion reason: Quantitative data not collected (only qualitative data collected) or no original data collected.

498. Wang Q-j. Promotion of outward bound teaching for college students' mental health and cohesion of class. *Journal of Wuhan Institute of Physical Education*. 2011;45(8):49-53.
Exclusion reason: No Translation Available.

499. Warber S, Bialko M, Dehudy A, Irvine K. Addressing nature deficit disorder: A quantitative survey study of multidimensional aspects of well-being among young adults at a wilderness camp. *BMC Complement Altern Med*. 2012;12.
Exclusion reason: No data collected on symptoms of mental illness.

500. Whall AL. Disruptive behavior in late stage dementia: using natural environments to decrease distress. *J Gerontol Nurs*. 1995;21(10):56-7.
Exclusion reason: quantitative data not collected (only qualitative data collected); no original data collected; or, data reported NOT usable (i.e. no Means/SD).

501. Whall AL. Geropsychiatry. Disruptive behavior in late stage dementia: using natural environments to decrease distress. *J Gerontol Nurs*. 1995:56-57.
Exclusion reason: Quantitative data not collected (only qualitative data collected) or no original data collected.

502. Wight EL. *The influence of a healthy work environment on perceived stress and stress-related health and behavioral outcomes: Panel analysis of office staff*. University of Florida; 2007.
Exclusion reason: Intervention did not occur outdoors or intervention does not meet study's definition of nature.

503. Wiley ER, Seabrook JA, Gilliland JA, Anderson KK, Stranges S. Green space and substance use and addiction: A new frontier. *Addict Behav*. 2020;100doi:10.1016/j.addbeh.2019.106155.
Exclusion reason: Quantitative data not collected (only qualitative data collected) or no original data collected.

504. Wilkinson N, Lanz C, Dundas C, et al. Adventure therapy: Peak experiences in early psychosis intervention. *Early intervention in psychiatry*. 2012;6:71. doi:10.1111/j.1751-7893.2012.00394.x.
Exclusion reason: No data collected that clearly defined nature dosage.

505. Wilson JF. *The relationship of outdoor recreation and gardening with depression among individuals with disabilities*. Utah State University; 2012.
Exclusion reason: only one time point collected.

506. Wilson JF, Christensen KM. The relationship between outdoor recreation and depression among individuals with disabilities. *Journal of Leisure Research*. 2012;44(4):486-506.
Exclusion reason: only one time point collected.

507. Witman JP, Preskenis K. Adventure programming with an individual who has multiple personality disorder: A case history. *Ther Recreation J*. 1996;30(4):289-296.
Exclusion reason: Quantitative data not collected (only qualitative data collected) or no original data collected.

508. Wolf M, Mehl K. Experiential learning in psychotherapy: ropes course exposures as an adjunct to inpatient treatment. *Clin Psychol Psychother*. 2011;18(1):60-74. doi:10.1002/cpp.692.
Exclusion reason: No data collected that clearly defined nature dosage.

509. Wollerman DM. *A leisure/recreational intervention to promote lifestyle change and prevent relapse in an alcoholic population*. Fairleigh Dickinson University; 1992.
Exclusion reason: Intervention did not occur outdoors or intervention does not meet study's definition of nature.

510. Woo JM, Kim W. Synergistic effect of forest environment and therapeutic program for the treatment of depression. *Asia-Pacific Psychiatry*. 2012;4:120. doi:10.1111/appy.12005.
Exclusion reason: No data collected that clearly defined nature dosage.

511. Wood CJ, Pretty J, Griffin M. A case-control study of the health and well-being benefits of allotment gardening. *Journal of Public Health*. 2016;38(3):e336-e344.
Exclusion reason: Intervention did not occur outdoors or intervention does not meet study's definition of nature.

512. Woodford KM, Fenton L, Connors J. A Change of Scenery: Wilderness Therapy Treatment for Inpatients in Acute Care. *Ther Recreation J*. 2017;51(4):258-273. doi:10.18666/TRJ-2017-V51-I4-7374.
Exclusion reason: Quantitative data not in article, not provided by author.

513. Wren CG, Hollenbaugh M. *Ammo for Education: An Eight-Week Program for Student Veteran Transition and Mental Health*. Texas A&M University, Corpus Christi; 2018.
Exclusion reason: Quantitative data not in article, not provided by author.

514. Wright A, Cattan M. Physical activity and the management of depression. *Working with Older People: Community Care Policy & Practice*. 2009;13(1):15-18. doi:10.1108/13663666200900004.
Exclusion reason: Quantitative data not collected (only qualitative data collected) or no original data collected.

515. Wüstemann H, Kolbe J, Krekel C. Health effects of urban green spaces: An empirical analysis. *Natur und Landschaft*. 2017;92(1):31-37. doi:10.17433/1.2017.50153433.31-37.
Exclusion reason: No Translation Available.

516. Xu Y, Qi J, Yang Y, Wen X. The contribution of lifestyle factors to depressive symptoms: A cross-sectional study in Chinese college students. *Psychiatry Res*. 2016;245:243-249. doi:10.1016/j.psychres.2016.03.009.
Exclusion reason: Only one time point collected.

517. Yamada S, Toba K. [A prospective comparison of day care and freely chosen occupational therapy for elderly patients with dementia]. *Nippon Ronen Igakkai Zasshi - Japanese Journal of Geriatrics*. 2005;42(1):83-9.
Exclusion reason: Intervention did not occur outdoors or intervention does not meet study's definition of nature.

518. Yamazaki S, Ura C, Okamura T, Shimmei M, Ishiguro T, Torishima K, Kawamuro Y. Long-term effects of rice-farming care on cognitive function and mental health of elderly people with cognitive impairment: a follow-up study. *Psychogeriatrics*. 2019;19(5):513-515. doi:10.1111/psyg.12409.
Exclusion reason: Intervention did not occur outdoors or intervention does not meet study's definition of nature.

519. Yavne Y, Kabaha A, Rosen T, Avisar I, Orbach H, Amital D, Amital H. The powers of flowers: Evaluating the impact of floral therapy on pain and psychiatric symptoms in fibromyalgia. *Isr Med Assoc J*. 2019;21(7):449-453.
Exclusion reason: Intervention did not occur outdoors or intervention does not meet study's definition of nature.

520. Yu YM, Lee YJ, Kim JY, Yoon SB, Shin CS. Effects of forest therapy camp on quality of life and stress in postmenopausal women. *Forest Science and Technology*. 2016;12(3):125-129. doi:10.1080/21580103.2015.1108248.
Exclusion reason: No data presented on participants' symptoms of mental illness.

521. Yuna L, Jiyoung L, Tulo NB. Korean Forest Taegyo for Reducing Stress and Emotional Instability during Pregnancy. *International Journal of Childbirth Education*. 2016;31(4):24-27.
Exclusion reason: Quantitative data not collected (only qualitative data collected) or no original data collected.

522. Yzoard M, Batt M, Trognon A, et al. Jardin thérapeutique et démences : Le dialogue comme unification des fonctions psychologiques = Therapeutic garden and dementia: Dialogue to assist in the unification of psychological functions. *NPG Neurologie - Psychiatrie - Gériatrie*. 2017;17(100):253-263. doi:10.1016/j.npg.2016.07.002.
Exclusion reason: Intervention did not occur outdoors or intervention does not meet study's definition of nature.

523. Zabag R, Deri O, Gilboa-Schechtman E, Richter-Levin G, Levy-Gigi E. Cognitive flexibility in PTSD individuals following nature adventure intervention: is it really that good? *Stress*. 2020;23(1):97-104. doi:10.1080/10253890.2019.1645113.
Exclusion reason: Only one time point collected.

524. Zerwig LM. *The Effects of Green Space Utilization among Nursing Students in Regards to Stress Level and Blood Pressure*. Brandman University; 2017.
Exclusion reason: No data collected on symptoms of mental illness.

525. Zhang Z. Outdoor group activity, depression, and subjective well-being among retirees of China: The mediating role of meaning in life. *J Health Psychol*. 2019;24(9):1245-1256. doi:10.1177/1359105317695428.

Exclusion reason: Only one time point collected.

526. Zhou C, Yan L, Yu L, et al. Effect of Short-term Forest Bathing in Urban Parks on Perceived Anxiety of Young-adults: A Pilot Study in Guiyang, Southwest China. *Chinese Geographical Science*. 2019;29(1):139-150. doi:10.1007/s11769-018-0987-x.
Exclusion reason: No data presented on participants' symptoms of mental illness.

527. Zhu S, Wan H, Lu Z, Wu H, Zhang Q, Qian X, Ye C. Treatment Effect of Antipsychotics in Combination with Horticultural Therapy on Patients with Schizophrenia: A Randomized, Double-blind, Placebo-controlled Study. *Shanghai Jingshen Yixue*. 2016;28(4):195-203. doi:10.11919/j.issn.1002-0829.216034.
Exclusion reason: Intervention did not occur outdoors or intervention does not meet study's definition of nature.

528. Zhu S-H, Lu Z-D, Wan H-J, Ye C-Y. Effect of horticultural therapy on metabolism indices in in-patients with chronic schizophrenia. *Chinese Mental Health Journal*. 2017;31(6):447-453.
Exclusion reason: Intervention did not occur outdoors or intervention does not meet study's definition of nature.

529. Zijlema WL, Avila-Palencia I, Triguero-Mas M, et al. Active commuting through natural environments is associated with better mental health: Results from the PHENOTYPE project. *Environ Int*. 2018;121(Pt 1):721-727. doi:10.1016/j.envint.2018.10.002.
Exclusion reason: No data collected that clearly defined nature dosage.

530. Zivkovic R, Lambic LJS, Zdravkovic M. The effect of mountain rehabilitation on heart rate, rate-pressure product, anxiety and depression in hypertensive women. *J Hypertens*. 2012;30:e154. doi:10.1097/01.hjh.0000420801.90161.29.
Exclusion reason: No data collected that clearly defined nature dosage.
