# Supplementary material for: A Systematic Review and Meta-Analysis on the Effect of Nature Exposure Dose on Adults with Mental Illness
Source: Behav Sci (Basel). 2025 Jan 31;15(2):153. doi: 10.3390/bs15020153 (PMC11851813; doi:10.3390/bs15020153)
Supplement: Supplementary file 1 [file behavsci-15-00153-s001.zip › Supplementary E for dosage-BibliographyInludedStudiesWithReasons.pdf]

Supplementary E: Bibliography of Included Studies

1. Antonioli C. *Biophilia: the therapeutic value of animals in the treatment of depression*. University of Leicester; 2010.

2. Bahaeloo-Horeh S, Assari S. Students experience self-esteem improvement during mountaineering. *Wilderness Environ Med*. 2008;19(3):181-5. doi:10.1580/07-WEME-OR-130.1.

3. Bang KS, Lee I, Kim S, Lim CS, Joh HK, Park BJ, Song MK. The Effects of a Campus Forest-Walking Program on Undergraduate and Graduate Students' Physical and Psychological Health. *Int J Environ Res Public Health*. 2017;14(7):05. doi:10.3390/ijerph14070728.

4. Barton J. *The effects of green exercise on psychological health and well-being*. University of Essex; 2009.

5. Barton J, Griffin M, Pretty J. Exercise-, nature- and socially interactive-based initiatives improve mood and self-esteem in the clinical population. *Perspectives in Public Health*. 2012;132(2):89-96.

6. Bennett JL, Piatt JA, Van Puymbroeck M. Outcomes of a Therapeutic Fly-Fishing Program for Veterans with Combat-Related Disabilities: A Community-Based Rehabilitation Initiative. *Community Ment Health J*. 2017;53(7):756-765. doi:10.1007/s10597-017-0124-9.

7. Berman MG, Kross E, Krpan KM, et al. Interacting with nature improves cognition and affect for individuals with depression. *J Affect Disord*. 2012;140(3):300-5. doi:10.1016/j.jad.2012.03.012.

8. Bettmann J, Tucker A, Behrens E, Vanderloo M. Changes in Late Adolescents and Young Adults' Attachment, Separation, and Mental Health During Wilderness Therapy. *Journal of Child & Family Studies*. 2017;26(2):511-522. doi:10.1007/s10826-016-0577-4.

9. Bettmann JE, Scheinfeld DE, Prince KC, Garland EL, Ovrom KV. Changes in psychiatric symptoms and psychological processes among veterans participating in a therapeutic adventure program. *Psychol Serv*. 2019;16(4):525-534. doi:10.1037/ser0000213.

10. Bielinis E, Jaroszewska A, Lukowski A, Takayama N. The Effects of a Forest Therapy Programme on Mental Hospital Patients with Affective and Psychotic Disorders. *Int J Environ Res Public Health*. 2019;17(1):23. doi:10.3390/ijerph17010118.

11. Bielinis E, Takayama N, Boiko S, Omelan A, Bielinis L. The effect of winter forest bathing on psychological relaxation of young Polish adults. *Urban Forestry and Urban Greening*. 2018;29:276-283. doi:10.1016/j.ufug.2017.12.006.

12. Bird K. Research evaluation of an Australian peer outdoor support therapy program for contemporary veterans’ wellbeing. *Int J Ment Health*. 2015;44(1-2):46-79. doi:10.1080/00207411.2015.1009752.

13. Bodin M, Hartig T. Does the outdoor environment matter for psychological restoration gained through running? *Psychol Sport Exerc*. 2003;4(2):141-153. doi:10.1016/S1469-0292(01)00038-3.

14. Bragg R. *Nature-based interventions for mental wellbeing and sustainable behaviour: the potential for green care in the UK*. University of Essex; 2014.

15. Brooks AM, Ottley KM, Arbuthnott KD, Sevigny P. Nature-related mood effects: Season and type of nature contact. *J Environ Psychol*. 2017;54:91-102. doi:10.1016/j.jenvp.2017.10.004.

16. Brown DK, Barton JL, Pretty J, Gladwell VF. Walks4Work: assessing the role of the natural environment in a workplace physical activity intervention. *Scand J Work Environ Health*. 2014;40(4):390-9. doi:10.5271/sjweh.3421.

17. Cervinka R, Schwab M, Haluza D. Investigating the Qualities of a Recreational Forest: Findings from the Cross-Sectional Hallerwald Case Study. *Int J Environ Res Public Health*. 2020;17(5):04. doi:10.3390/ijerph17051676.

18. Chun MH, Chang MC, Lee SJ. The effects of forest therapy on depression and anxiety in patients with chronic stroke. *Int J Neurosci*. 2017;127(3):199-203. doi:10.3109/00207454.2016.1170015.

19. Corazon SS, Sidenius U, Vammen KS, Klinker SE, Stigsdotter UK, Poulsen DV. The Tree Is My Anchor: A Pilot Study on the Treatment of BED through Nature-Based Therapy. *Int J Environ Res Public Health*. 2018;15(11):08. doi:10.3390/ijerph15112486.

20. Cotton S, Butselaar F. Outdoor adventure camps for people with mental illness. *Australasian Psychiatry*. 2013;21(4):352-8. doi:10.1177/1039856213492351.

21. Crawford RT. *The impact of ocean therapy on veterans with posttraumatic stress disorder*. Grand Canyon University; 2017.

22. Crust L, Henderson H, Middleton G. The acute effects of urban green and countryside walking on psychological health: A field-based study of green exercise. *Int J Sport Psychol*. 2013;44(2):160-177.

23. de Brito JN, Pope ZC, Mitchell NR, Schneider IE, Larson JM, Horton TH, Pereira MA. Changes in Psychological and Cognitive Outcomes after Green versus Suburban Walking: A Pilot Crossover Study. *Int J Environ Res Public Health*. 2019;16(16):13. doi:10.3390/ijerph16162894.

24. Duvall J, Kaplan R. Enhancing the well-being of veterans using extended group-based nature recreation experiences. *J Rehabil Res Dev*. 2014;51(5):685-96.

25. Eikenaes I, Gude T, Hoffart A. Integrated wilderness therapy for avoidant personality disorder. *Nordic Journal of Psychiatry*. 2006;60(4):275-81.

26. Eko M. *A portfolio of academic, therapeutic practice and research work: including an investigation of spending time in nature: restorative effects of mood amongst depressed individuals*. University of Surrey; 2015.

27. Furuyashiki A, Tabuchi K, Norikoshi K, Kobayashi T, Oriyama S. A comparative study of the physiological and psychological effects of forest bathing (Shinrin-yoku) on working age people with and without depressive tendencies. *Environ Health Prev Med*. 2019;24(1):46. doi:10.1186/s12199-019-0800-1.

28. Gelkopf M, Hasson-Ohayon I, Bikman M, Kravetz S. Nature adventure rehabilitation for combat-related posttraumatic chronic stress disorder: a randomized control trial. *Psychiatry Res*. 2013;209(3):485-93. doi:10.1016/j.psychres.2013.01.026.

29. Gonzalez MT, Hartig T, Patil GG, Martinsen EW, Kirkevold M. Therapeutic horticulture in clinical depression: a prospective study. *Res Theory Nurs Pract*. 2009;23(4):312-28.

30. Gonzalez MT, Hartig T, Patil GG, Martinsen EW, Kirkevold M. Therapeutic horticulture in clinical depression: a prospective study of active components. *J Adv Nurs*. 2010;66(9):2002-13. doi:10.1111/j.1365-2648.2010.05383.x.

31. Gonzalez MT, Hartig T, Patil GG, Martinsen EW, Kirkevold M. A prospective study of existential issues in therapeutic horticulture for clinical depression. *Issues Ment Health Nurs*. 2011;32(1):73-81. doi:10.3109/01612840.2010.528168.

32. Hayashi N, Wada T, Hirai H, et al. The effects of horticultural activity in a community garden on mood changes. *Environmental Control in Biology*. 2008;46(4):233-240. doi:10.2525/ecb.46.233.

33. Hitter T, Kállay É, Olar LE, et al. The effect of therapeutic horticulture activities on people in depression and kynurenine pathways. *Notulae Botanicae Horti Agrobotanici Cluj-Napoca*. 2019;47(3):804-812. doi:10.15835/nbha47311544.

34. Ibes DC, Forestell CA. The role of campus greenspace and meditation on college students' mood disturbance. *J Am Coll Health*. 2020:1-8. doi:10.1080/07448481.2020.1726926.

35. Iwata Y, Dhubháin AN, Brophy J, Roddy D, Burke C, Murphy B. Benefits of Group Walking in Forests for People with Significant Mental Ill-Health. *Ecopsychology*. 2016;8(1):16-26. doi:10.1089/eco.2015.0045.

36. Janeczko E, Bielinis E, Wójcik R, et al. When urban environment is restorative: The effect of walking in suburbs and forests on psychological and physiological relaxation of young polish adults. *Forests*. 2020;11(5)doi:10.3390/F11050591.

37. Kelley MP, Coursey RD, Selby PM. Therapeutic adventures outdoors: A demonstration of benefits for people with mental illness. *Psychiatric Rehabilitation Journal*. 1997;20(4):61-73.

38. Kim JG, Khil TG, Lim Y, Park K, Shin M, Shin WS. The Psychological Effects of a Campus Forest Therapy Program. *Int J Environ Res Public Health*. 2020;17(10):14. doi:10.3390/ijerph17103409.

39. Kim W, Lim SK, Chung EJ, Woo JM. The effect of cognitive behavior therapy-based psychotherapy applied in a forest environment on physiological changes and remission of major depressive disorder. *Psychiatry Investig*. 2009;6(4):245-54. doi:10.4306/pi.2009.6.4.245.

40. Kjellgren A, Buhrkall H. A comparison of the restorative effect of a natural environment with that of a simulated natural environment. *J Environ Psychol*. 2010;30(4):464-472. doi:10.1016/j.jenvp.2010.01.011.

41. Klaperski S, Koch E, Hewel D, Schempp A, Müller J. Optimizing mental health benefits of exercise: The influence of the exercise environment on acute stress levels and wellbeing. *Mental Health and Prevention*. 2019;15doi:10.1016/j.mhp.2019.200173.

42. Korpela KM, Stengård E, Jussila P. Nature walks as a part of therapeutic intervention for depression. *Ecopsychology*. 2016;8(1):8-15. doi:10.1089/eco.2015.0070.

43. Lachance D. *What Nature Is Best?: Testing the Effects of Green and Blue Space on Mood and Environmental Concern*. Trent University; 2020.

44. Lee J, Park BJ, Tsunetsugu Y, Ohira T, Kagawa T, Miyazaki Y. Effect of forest bathing on physiological and psychological responses in young Japanese male subjects. *Public Health*. 2011;125(2):93-100. doi:10.1016/j.puhe.2010.09.005.

45. Lee J, Tsunetsugu Y, Takayama N, et al. Influence of forest therapy on cardiovascular relaxation in young adults. *Evidence-Based Complementary & Alternative Medicine: eCAM*. 2014;2014:834360. doi:10.1155/2014/834360.

46. Lee JH, Park JS, Choi S. Environmental influence in the forested area toward human health: incorporating the ecological environment into art psychotherapy. *Journal of Mountain Science*. 2020;17(4):992-1000. doi:10.1007/s11629-019-5774-3.

47. Legrand FD, Race M, Herring MP. Acute effects of outdoor and indoor exercise on feelings of energy and fatigue in people with depressive symptoms. *J Environ Psychol*. 2018;56:91-96. doi:10.1016/j.jenvp.2018.03.005.

48. Lundberg N, Bennett J, Smith S. Outcomes of Adaptive Sports and Recreation Participation among Veterans Returning from Combat with Acquired Disability. *Ther Recreation J*. 2011;45(2):105-120.

49. Mackay GJ, Neill JT. The effect of 'green exercise' on state anxiety and the role of exercise duration, intensity, and greenness: A quasi-experimental study. *Psychol Sport Exerc*. 2010;11(3):238-245. doi:10.1016/j.psychsport.2010.01.002.

50. Makizako H, Tsutsumimoto K, Doi T, Makino K, Nakakubo S, Liu-Ambrose T, Shimada H. Exercise and Horticultural Programs for Older Adults with Depressive Symptoms and Memory Problems: A Randomized Controlled Trial. *Journal of Clinical Medicine*. 2019;9(1):30. doi:10.3390/jcm9010099.

51. Marchand WR, Klinger W, Block K, et al. Mindfulness Training plus Nature Exposure for Veterans with Psychiatric and Substance Use Disorders: A Model Intervention. *Int J Environ Res Public Health*. 2019;16(23):27. doi:10.3390/ijerph16234726.

52. Marchand WR, Klinger W, Block K, et al. Safety and psychological impact of sailing adventure therapy among Veterans with substance use disorders. *Complement Ther Med*. 2018;40:42-47. doi:10.1016/j.ctim.2018.07.013.

53. Maund PR, Irvine KN, Reeves J, Strong E, Cromie R, Dallimer M, Davies ZG. Wetlands for Wellbeing: Piloting a Nature-Based Health Intervention for the Management of Anxiety and Depression. *Int J Environ Res Public Health*. 2019;16(22):11. doi:10.3390/ijerph16224413.

54. McCaffrey R, Hanson C, McCaffrey W. Garden walking for depression: a research report. *Holist Nurs Pract*. 2010;24(5):252-9. doi:10.1097/HNP.0b013e3181f1acd7.

55. McCaffrey R, Liehr P, Gregersen T, Nishioka R. Garden walking and art therapy for depression in older adults: a pilot study. *Res Gerontol Nurs*. 2011;4(4):237-42. doi:10.3928/19404921-20110201-01.

56. Morita E, Fukuda S, Nagano J, et al. Psychological effects of forest environments on healthy adults: Shinrin-yoku (forest-air bathing, walking) as a possible method of stress reduction. *Public Health*. 2007;121(1):54-63.

57. Ochiai H, Ikei H, Song C, et al. Physiological and psychological effects of forest therapy on middle-aged males with high-normal blood pressure. *Int J Environ Res Public Health*. 2015;12(3):2532-42. doi:10.3390/ijerph120302532.

58. Oh YA, Park SA, Ahn BE. Assessment of the psychopathological effects of a horticultural therapy program in patients with schizophrenia. *Complement Ther Med*. 2018;36:54-58. doi:10.1016/j.ctim.2017.11.019.

59. Olafsdottir G, Cloke P, Schulz A, van Dyck Z, Eysteinsson T, Thorleifsdottir B, Vögele C. Health benefits of walking in nature: A randomized controlled study under conditions of real-life stress. *Environ Behav*. 2020;52(3):248-274. doi:10.1177/0013916518800798.

60. Pawlowski M, Holme G, Hafner RJ. Wilderness therapy for psychiatric disorder. *Mental Health in Australia*. 1993;5(1):8-14.

61. Pretty J, Peacock J, Hine R, Sellens M, South N, Griffin M. Green exercise in the UK countryside: Effects on health and psychological well-being, and implications for policy and planning. *Journal of Environmental Planning and Management*. 2007;50(2):211-231. doi:10.1080/09640560601156466.

62. Richardson ED. *Adventure-based therapy and self-efficacy theory: Test of a treatment model for late adolescents with depressive symptomatology*. Virginia Polytechnic Institute and State University; 2003.

63. Rogerson M, Colbeck I, Bragg R, Dosumu A, Griffin M. Affective Outcomes of Group versus Lone Green Exercise Participation. *Int J Environ Res Public Health*. 2020;17(2):18. doi:10.3390/ijerph17020624.

64. Rogerson M, Gladwell VF, Gallagher DJ, Barton JL. Influences of Green Outdoors versus Indoors Environmental Settings on Psychological and Social Outcomes of Controlled Exercise. *Int J Environ Res Public Health*. 2016;13(4):363. doi:10.3390/ijerph13040363.

65. Saadi D, Schnell I, Tirosh E, Basagana X, Agay-Shay K. There's no place like home? The psychological, physiological, and cognitive effects of short visits to outdoor urban environments compared to staying in the indoor home environment, a field experiment on women from two ethnic groups. *Environ Res*. 2020;187:109687. doi:10.1016/j.envres.2020.109687.

66. Stigsdotter UK, Corazon SS, Sidenius U, Nyed PK, Larsen HB, Fjorback LO. Efficacy of nature-based therapy for individuals with stress-related illnesses: randomised controlled trial. *Br J Psychiatry*. 2018;213(1):404-411. doi:10.1192/bjp.2018.2.

67. Stowell DR, Owens GP, Burnett A. A pilot horticultural therapy program serving veterans with mental health issues: Feasibility and outcomes. *Complement Ther Clin Pract*. 2018;32:74-78. doi:10.1016/j.ctcp.2018.05.007.

68. Thal L. *Attention-deficit hyperactivity disorder and exposure to nature in college students*. University of Missouri, Columbia; 2014.

69. Tsunetsugu Y, Park BJ, Lee J, Kagawa T, Miyazaki Y. [Psychological relaxation effect of forest therapy: results of field experiments in 19 forests in Japan involving 228 participants]. *Nippon Eiseigaku Zasshi - Japanese Journal of Hygiene*. 2011;66(4):670-6.

70. Tyrväinen L, Ojala A, Korpela K, Lanki T, Tsunetsugu Y, Kagawa T. The influence of urban green environments on stress relief measures: A field experiment. *J Environ Psychol*. 2014;38:1-9. doi:10.1016/j.jenvp.2013.12.005.

71. Van Den Berg AE, Custers MH. Gardening promotes neuroendocrine and affective restoration from stress. *J Health Psychol*. 2011;16(1):3-11. doi:10.1177/1359105310365577.

72. Vella EJ, Milligan B, Bennett JL. Participation in outdoor recreation program predicts improved psychosocial well-being among veterans with post-traumatic stress disorder: a pilot study. *Mil Med*. 2013;178(3):254-60. doi:10.7205/MILMED-D-12-00308.

73. Wilson NW, Jones R, Fleming S, Lafferty K, Knifton L, Cathrine K, McNish H. Branching Out: The impact of a mental health ecotherapy program. *Ecopsychology*. 2011;3(1):1-7. doi:10.1089/eco.2010.0049.

74. Wirz-Justice A, Graw P, Krauchi K, Sarrafzadeh A, English J, Arendt J, Sand L. 'Natural' light treatment of seasonal affective disorder. *J Affect Disord*. 1996;37(2-3):109-20.

75. Yeon Hee C, Young Sun H. The Effectiveness of a Forest-experience-integration Intervention for Community Dwelling Cancer Patients' Depression and Resilience. *Journal of Korean Academy of Community Health Nursing / Jiyeog Sahoe Ganho Hakoeji*. 2014;25(2):109-118. doi:10.12799/jkachn.2014.25.2.109.

76. Yu CP, Lin CM, Tsai MJ, Tsai YC, Chen CY. Effects of Short Forest Bathing Program on Autonomic Nervous System Activity and Mood States in Middle-Aged and Elderly Individuals. *Int J Environ Res Public Health*. 2017;14(8):09. doi:10.3390/ijerph14080897.

77. Yu CPS, Hsieh H. Beyond restorative benefits: Evaluating the effect of forest therapy on creativity. *Urban Forestry and Urban Greening*. 2020;51doi:10.1016/j.ufug.2020.126670.

78. Zebrack B, Kwak M, Sundstrom L. First Descents, an adventure program for young adults with cancer: who benefits? *Support Care Cancer*. 2017;25(12):3665-3673. doi:10.1007/s00520-017-3792-7.
